# Proteomic profiling identifies the inorganic pyrophosphatase (PPA1) protein as a potential biomarker of metastasis in laryngeal squamous cell carcinoma

**DOI:** 10.1007/s00726-016-2201-8

**Published:** 2016-03-07

**Authors:** Magdalena Bodnar, Magdalena Luczak, Kinga Bednarek, Lukasz Szylberg, Andrzej Marszalek, Reidar Grenman, Krzysztof Szyfter, Malgorzata Jarmuz-Szymczak, Maciej Giefing

**Affiliations:** Department of Clinical Pathomorphology, Collegium Medicum in Bydgoszcz, Nicolaus Copernicus University in Torun, Bydgoszcz, Poland; Institute of Bioorganic Chemistry, Polish Academy of Sciences, Poznan, Poland; Department of Cancer Genetics, Institute of Human Genetics, Polish Academy of Sciences, Poznan, Poland; Department of Otorhinolaryngology, Head and Neck Surgery, Turku University Hospital and University of Turku, Turku, Finland; Department of Medical Biochemistry, Turku University Hospital and University of Turku, Turku, Finland; Institute of Chemical Technology and Engineering, Poznan University of Technology, Poznan, Poland; Chair of Oncologic Pathology and Epidemiology, Poznan University of Medical Sciences and Greater Poland Cancer Center, Poznan, Poland; Department of Audiology and Phoniatrics, Poznan University of Medical Sciences, Poznan, Poland; Department of Hematology and Bone Marrow Transplantation, Poznan University of Medical Sciences, Poznan, Poland; Department of Otolaryngology and Laryngeal Oncology, Poznan University of Medical Sciences, Poznan, Poland

**Keywords:** LSCC, Laryngeal cancer, PPA1, Metastasis, Biomarker

## Abstract

Relapse and metastasis are the main causes of unfavorable outcome in head and neck cancers. Whereas, understanding of the molecular background of these processes is far from being complete. Therefore, in this study we aimed to identify potential biomarker candidates of relapse and metastasis in laryngeal squamous cell carcinoma (LSCC) by combining the 2D electrophoresis based protein screen and immunohistochemical analysis of candidate proteins. We screened three groups of LSCC cell lines derived from primary tumors, recurrent tumors and metastases and identified seven proteins that differed significantly in relative abundance between the analyzed groups. Among the identified proteins were the heat shock proteins HSP60 and HSP70 that were significantly downregulated both in recurrences- and metastases-derived cell lines but not in primary tumor-derived cell lines. Moreover, we identified significant upregulation of the annexin V, calreticulin and the inorganic pyrophosphatase (PPA1) exclusively in the metastases-derived cell lines. As these upregulated proteins could potentially become novel biomarkers of metastasis, we have compared their abundance in primary tumor LSCC N(0) cases, primary tumor LSCC N(+) cases as well as in LSCC metastases N(+). Our results show an intense increase of cytoplasmic PPA1 abundance in the N(+) (*p* = 0.000042) compared to the N(0) group. In summary, we show a group of proteins deregulated in recurrences and metastases of LSCC. Moreover, we suggest the PPA1 protein as a potential new biomarker for metastasis in this cancer.

**Electronic supplementary material:**

The online version of this article (doi:10.1007/s00726-016-2201-8) contains supplementary material, which is available to authorized users.

## Introduction

Laryngeal squamous cell carcinoma (LSCC) is one of the most common cancers of the head and neck in males in European countries. Although the age standardized incidence for men decreased in the last two decades still 8.8 per 100,000 are diagnosed yearly with the disease (Ferlay et al. 2013). LSCC patients’ survival rates are unsatisfactory low with the 5-year survival of 67 % (De Angelis et al. 2014) and decrease dramatically when the tumor is diagnosed in advanced stage, relapses or metastasizes.

Several proteins have been reported so far to correlate with progression and/or metastases of LSCC tumors including the up-regulation of matrix metalloproteinases (MMPs) (Görögh et al. 2006; Uloza et al. 2011; Bodnar et al. 2014); cyclin D1 (CCND1) (Zhang et al. 2013; Jarmuz-Szymczak et al. 2013) or the downregulation of tumor suppressive proteins TSLC1 (Lu et al. 2012) or PCDH17 (Haruki et al. 2010; Giefing et al. 2011). Moreover, Cai et al. (2012) investigated transcription regulation networks to identify regulators for lymph node metastasis in LSCC and showed SP1 and c-MYC transcription factors, which may affect LSCC metastasis by promoting the epithelialmesenchymal transition (Cai et al. 2012).

Despite, recent advances in identifying proteins related to the metastatic potential of LSCC the understanding of the molecular background of the metastatic process is far from being complete. This necessitates the identification of proteins associated with recurrence or metastasis that could possibly become biomarkers of metastasis and targets of novel personalized therapies.

In this study, by comparing the 2D proteome profiles of LSCC cell lines derived from primary tumors (*n* = 11), recurrent tumors (*n* = 9) and metastases (*n* = 6), we identified seven proteins that were significantly differentially represented in these groups. Moreover, we compared the abundance of the best protein marker candidates by immunohistochemistry in primary LSCC tumor sections (20 N(0) tumors, 20 N(+) tumors and 20 lymph node SCC metastases) and validated the findings from cell lines.

## Material and methods

### LSCC cell lines and culture conditions

Twenty-six cell lines established at the University of Turku, Finland, from patients diagnosed with LSCC were analyzed. The characteristics of the original material taken to establish the cell lines are shown in the supplementary (Table S1). Most of the cell lines were characterized in earlier studies (Jarmuz et al. 2002, 2005; Järvinen et al. 2006; Giefing et al. 2008). We have confirmed the stability of the used cell lines in earlier experiments by karyotyping on different passages. Moreover, array-CGH profiles as well as mRNA expression profiles of these cell lines present typical LSCC characteristics including copy number changes of specific genomic regions and transcriptional deregulation of tumor related genes (Kostrzewska-Poczekaj et al.; Giefing et al. 2011; Jarmuz et al. 2002, 2005; Järvinen et al. 2006). The cells were grown in 25-cm^2^ flasks in Dulbecco’s modified medium supplemented with 10 % fetal bovine serum at 37 °C under 5 % CO_2_. For protein extraction, the cell lines were cultured to 80 % confluence, and then harvested with 0.1 % trypsin and 0.2 % EDTA.

### LSCC tumor sections

#### Patients

The studies were performed on a group of 40 patients, 35 men (age 44–77) and 5 women (age 50–69), who underwent total laryngectomy in the Department of Otolaryngology and Clinical Oncology Chair and Clinic of Otolaryngology Collegium Medicum in Bydgoszcz, Nicolaus Copernicus University in Torun, Poland. According to histopathological examination, performed by two independent pathologists, squamous cell carcinoma (SCC) was diagnosed in all patients. The tumor stage was determined according to the current TNM classification of the International Union Against Cancer (IUAC) as follows: pT2—1 case, pT3—27 cases, pT4—12 cases; pN0—20 cases, pN(+)—20 cases. No distant metastases (beyond loco-regional lymph nodes) were found in the analyzed groups.

#### Material

The immunohistochemical studies were performed on selected archival formalin fixed paraffin embedded tissue sections, which contain a representative area of squamous cell carcinoma, derived from Department of Clinical Pathology, Collegium Medicum in Bydgoszcz, Nicolaus Copernicus University in Torun (Poland). All cases were revised and selected, according to HE stained tissue sections, by two independent pathologists. In each sample, cancer cells occupied approximately 80 % of tissue area, and the control sections contained a disease free normal mucosa, which were taken at least 2 cm away from the tumor resection margins.

The analyzed tissue material was divided into three groups: first group contained 20 tissue sections of primary LSCC from patients without lymph node metastases N(0), the second group contained 20 representative tissue sections of primary LSCC from patients with lymph node metastases N(+) and the third group contained 20 representative tissue sections of lymph nodes containing SCC metastases.

### Total protein isolation form LSCC cell lines

Cells were washed with PBS and then lysed in IEF buffer (7 M urea, 2 M thiourea, 2 % CHAPS 55 mM DTT and 0.5 % v/v IPG buffer). Cell lysis was facilitated by sonication in three cycles. Insoluble material was removed by centrifugation at 16 000*g* for 20 min. Protein concentration was estimated using a commercial 2-D Quant kit (GE Healthcare).

### 2D protein electrophoresis

Protein separations and image analysis were carried out as previously described (Luczak et al. 2012). 24 cm IPG strips (pH 4-7, GE Healthcare) were actively rehydrated overnight in IEF buffer containing cell samples. Each strip was loaded with the same amount of protein (650 µg). The strips were subjected to IEF on IPGphor III (GE Healthcare) using a ramping voltage (50–8000 V) to final 75,000 Vh. Second dimension was performed on 11 % polyacrylamide gels using the Ettan DALT six system (GE Healthcare). For each sample, a 2D analysis was repeated three times. After electrophoresis, gels were stained with Blue Silver overnight (Candiano et al. 2004) and scanned with the Umax scanner (GE Healthcare) using LabScan program.

The images were analyzed using the Image Master Platinum software version 6.0 (GE Healthcare) and Progenesis SameSpots (Nonlinear Dynamics). The relative abundance of each spot (% vol) was calculated as its volume divided by the total volume of matched spots. To find differently accumulated proteins, gap and ratio measures were taken into account. For differentiating proteins threshold 1.5 was selected.

Analysis of variance between all groups was performed using the ANOVA test to check significant differences between the % volume of each spot in all groups of samples. The Mann–Whitney *U* test was performed to compare between two particular groups. *P* values <0.05 were considered statistically significant. All statistical analyses were performed using Statistica ver. 8.0 software.

### In-gel digestion

Individual protein spots were cut into small pieces, rinsed twice in 100 μL of washing buffer (50 mM NH_4_HCO_3_/100 % CH_3_CN (vol. 1:1)) for 15 min and dehydrated in 100 % CH_3_CN. The dried gel spots were rehydrated by the addition of 10 μL of digestion buffer [25 mM ammonium bicarbonate and 0.2 μg of sequencing-grade trypsin (Promega)]. Digestion was performed overnight at 37 °C. Peptides were extracted with 10 % CH_3_CN.

### Mass spectrometry

Proteins were identified using the MALDI-TOF/TOF mass spectrometer. The MALDI spectra were acquired on an UltrafleXtreme (Bruker Daltonics, Germany) mass spectrometer operated in reflector mode using delayed ion extraction. Positively charged ions in the *m*/*z* range of 800–3500 were analyzed. For each sample, 0.5 µl was co-crystallized with CHCA matrix and spotted directly onto the MALDI AnchorChip target (Bruker Daltonics, Germany). The MS spectra were externally calibrated using the Peptide Calibration Standard mixture (Bruker Daltonics, Germany). Flex control v. 3.3 was used for the acquisition of spectra, and all further data processing was performed using Flex analysis v. 3.3. Protein database searches using combined MS and MS/MS datasets were performed via BioTools 3.2 (Bruker Daltonics, Germany). Proteins were identified using the Mascot (Matrix Science, London, UK) program against the SwissProt database (2012; 538 010 sequences in total). The false discovery rate (FDR) for peptide identification was 0.05 in all analyses. The protein search was performed using the following search parameters: precursor-ion mass tolerance, ±0.1 Da; fragment-ion mass tolerance, ±0.4 Da and cysteine-treated with iodoacetamide to form carbamidomethyl-cysteine and oxidized methionine. Trypsin was set as the enzyme, with a maximum of one missed cleavage. Proteins were identified on the basis of at least two unique peptides with peptide score higher than 40 (*p* < 0.05). All analyses were done in triplicate.

The authors apologize and take the full responsibility for the missing peak lists and raw spectra of the analyzed proteins. These raw data were lost and are not available anymore. Instead, MALDI analyses in HTML format are available on request.

### Immunohistochemistry

To determine the appropriate antibody dilution, eliminate false positive results, and reduce the background staining, a series of positive control reactions were performed on a model tissue selected according to antibody datasheets, and reference sources (The Human Protein Atlas: http://www.proteinatlas.org (Uhlen et al. 2010). The positive control for selected antibodies is described in Table S2. Negative immunohistochemical control reactions were performed, by substituting the primary antibody with diluted 1 % BSA (bovine serum albumin) in PBS (phosphate buffered saline).

Selected paraffin blocks were cut using the manual rotary microtome (AccuCut, Sakura, Torrance, USA), to 3.0 µm thick paraffin sections, placed on extra adhesive slides (SuperFrostPlus, MenzelGlasser, Braunschweig, Germany) and dried at 60 °C for 1 h.

Deparaffinization, rehydration and antigen retrieval were performed automatically in high-pH antigen retrieval solution (cat. No K8004, Dako, Glostrup, Denmark) using PT-Link (Dako). Subsequently, sections were incubated for 10 min with 3 % H_2_O_2_, in room temperature to block the endogenous peroxidase, and furthermore, non-specific binding was blocked by incubation with 5 % BSA diluted in PBS for 10 min in room temperature. The detection of antigens was performed using primary mouse/rabbit monoclonal antibodies (Table S2). Then, the sections were incubated for 20 min in 37 °C with peroxidase detection system, Anti-Mouse/Anti-Rabbit EnVision Flex-HRP Labeled Polymer (cat. No K8000, Dako), and with DAB (3-3′diaminobenzidine) as a chromogen. Finally, the sections were counterstained with hematoxylin, dehydrated in increasing ethanol concentrations (80, 90, 96, 99.8 %), cleared in xylenes (I–IV), and mounted with Shandon Consul Mount (Thermo Scientific, Waltham, USA).

### Immunohistochemical analysis of protein abundance

The evaluation of protein abundance was performed in three hot spots (the tumor area with the highest immunostaining), in the light microscope ECLIPSE E800 (Nikon Instruments Europe, Amsterdam, Netherlands) under 20× original objective magnification, equipped with cooled CD camera (Nikon Digital Sight DS-5Mc; Germany) driven by NIS Elements F 3.0 software (Nikon; Germany).

The level of selected protein abundance was evaluated using Remmele–Stegner scale (Remmele and Stegner 1987). Using the IRS scoring system, the percentage of positive stained cells/area (PP) and the intensity of the color reaction (SI) were taken into account. The final protein abundance (IRS = SI × PP) was presented as the ratio of the positively stained cells/area (PP) (scale 0–3; 0 = no staining, 1 <10 % stained cells/area; 2 = 10–50 % stained cells/area; 3 ≥50 % stained cells/area), and the intensity of protein abundance (SI) (scale 0–3; 0—no protein, 1—weak; 2—moderate; 3—strong abundance).

All calculations were performed using STATISTICA 10 (StatSoft). Normal distribution was tested using the Kolmogorov–Smirnov test with Lilliefors correction and the Shapiro–Wilk test and the variance was assessed using Levene’s test. For the statistical analysis *U* Mann–Whitney nonparametric test was used, and *p* < 0.05 was considered as statistically significant.

## Results

### 2D comparative proteome profiling of LSCC cell lines derived from primary tumors, recurrences and metastases

Total protein extracts from 26 LSCC cell lines were subjected to 2D comparative proteome profiling. The cell lines were divided into three groups—derived from primary tumors (*n* = 11), recurrent tumors (*n* = 9) and metastases (*n* = 6). The proteome profiles between individual cell lines in the respective groups reveal some level of heterogeneity but the differences in number of protein spots detected in a cell line does not exceed 20 %. Moreover, no significant differences in the number of protein spots were observed between the analyzed groups.

After electrophoresis proteins were extracted from the gel and examined by MALDI-TOF/TOF mass spectrometry. As a result, 122 proteins were identified. For all identified proteins the relative levels of their abundance in each sample were determined (mean from three analyses).

**Fig. 1:**
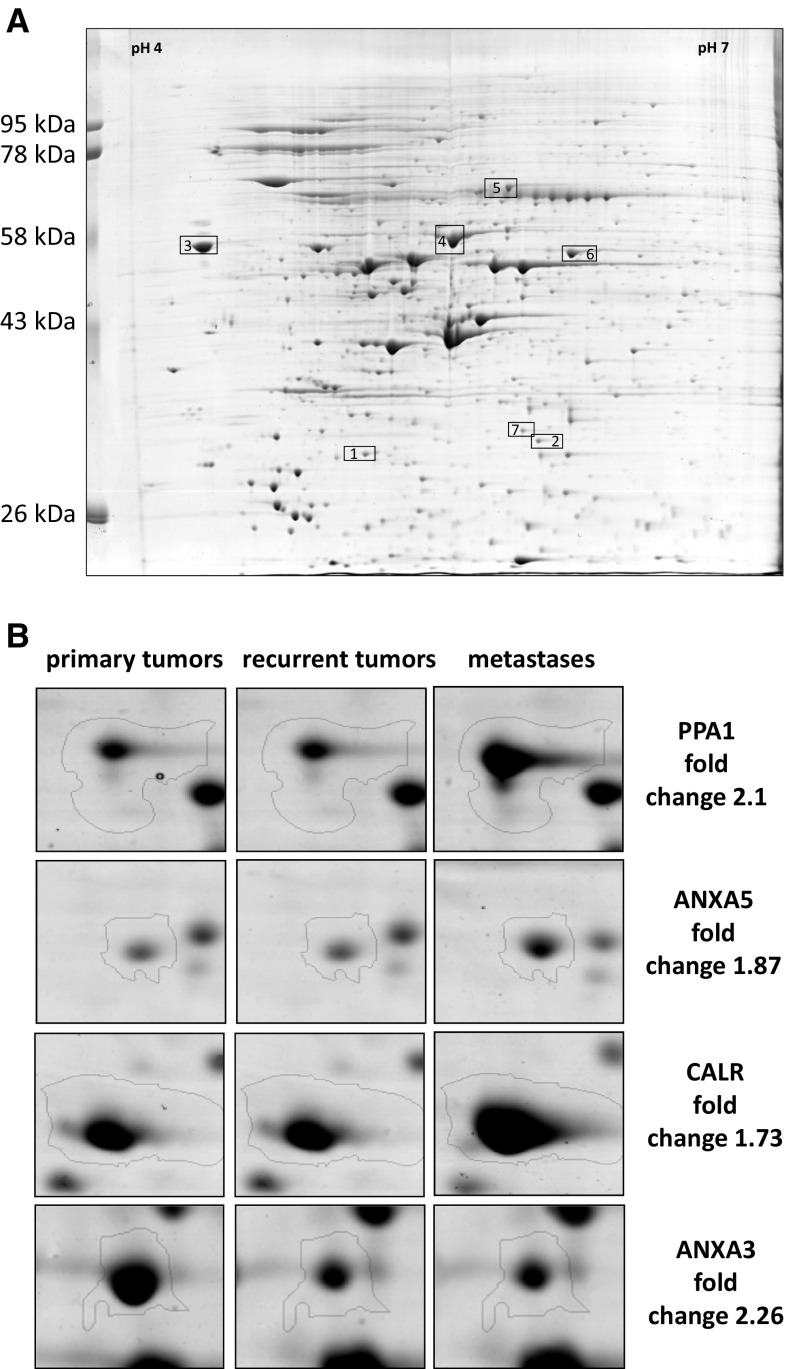
**a** Representative example of the 2D PAGE analysis of primary tumor cell line. IEF was performed in pH 4–7. The proteins showing different abundance identified by mass spectrometry are indexed by *numbers* and *boxed* (see Table 1). **b** Examples of differentially expressed proteins

Seven protein spots showed recurrent and significant differences in relative abundance between the three analyzed groups. These spots were identified as the following proteins: Annexin III (ANXA3), Annexin V (ANXA5), calreticulin (CALR), heat shock protein 60 (HSP60), heat shock 70 kDa protein 9B (HSP70), inorganic pyrophosphatase (PPA1) and protein disulphide isomerase (PDIA3). Three of these proteins, namely HSP60 (*p* = 0.0097; *p* = 0.0019), HSP70 (*p* = 0.0074; *p* = 0.0011) and ANXA3 (*p* = 0.00065; *p* = 0.0011) were significantly downregulated both in the groups of recurrent tumors as well as metastases as compared to the group of primary tumors, respectively, and were not further studied. Whereas, ANXA5 (*p* = 0.025), CALR (*p* = 0.0019) and PPA1 (*p* = 0.0031) showed significant upregulation and PDIA3 (*p* = 0.00092) downregulation only in the group of metastases as compared to the group of primary tumors (Table 1; Fig. 1).

**Table 1:** Short characteristic of the proteins showing different abundance between primary, recurrent tumors and metastases (2D comparative proteome profiling)

Spot no.	Protein identification	Accession (SwissProt)	Sequence coverage (%)	Score	Peptides matched	Theoretical MW (kDa)	Theoretical pI	Fold change (compared to primary tumors)	
								Recurrent	Metastases
1	Annexin V	ANXA5_HUMAN	51	163	16	35.9	4.94	0.83	**1.63**
2	Annexin III	ANXA3_HUMAN	30	108	10	36.5	5.63	**0.42**	**0.50**
3	Calreticulin	CALR_HUMAN	51	172	19	48.2	4.29	0.95	**1.74**
4	60 kDa heat shock protein	CH60_HUMAN	49	177	28	61.1	5.7	**0.72**	**0.54**
5	Heat shock 70 kDa protein 9	GRP75_HUMAN	41	170	23	73.9	5.87	**0.71**	**0.64**
6	Protein disulfide-isomerase A3	PDIA3_HUMAN	32	85	12	57.1	5.98	0.84	**0.52**
7	Inorganic pyrophosphatase PPA1	IPYR_HUMAN	52	161	12	33	5.54	1.02	**2.10**

Fold change <1 means downregulation; significant differences are bolded

In summary, elevated levels of ANXA5, CALR and PPA1 proteins were identified as potential markers of metastases.

### Immunohistochemical validation of ANXA5, CALR and PPA1 abundance in laryngeal squamous cell carcinoma

To validate the differential abundance of the three metastatic marker candidates and to analyze their subcellular localization the immunohistochemical staining (IHC) was performed on 40 primary LSCC tissue sections including 20 N(0), 20 N(+) cases, and 20 metastases of squamous cell carcinoma in lymph nodes [derived from the N(+) patients].

The analyses revealed membrane-cytoplasmatic abundance of ANXA5 protein. The ANXA5 protein was found in 95 % cases of N(0) LSCC, in 75 % cases of N(+) LSCC, and in 70 % cases of meta SCC in lymph nodes. The mean percentage of positive cells in given cases was 8 % in N(0), 6.6 % in N(+) and 8.8 % in metastatic tumors and the total IRS score was 2.1, 1.7 and 1.9, respectively. No significant differences in abundance of the studied protein were observed in the analyzed groups.

Similarly, the CALR protein was observed in the cytoplasm and membrane. We observed much higher percentage of positive cells in the N(+) group (79 %) and the metastatic tumors (44 %) compared to the N(0) group (37 %). However, the total IRS scale was similar in all studied groups: IRS = 4 for N(0), IRS = 3.5 for N(+) and IRS = 4 for the metastatic tumors. Moreover, no statistical differences of CALR abundance were observed among the three groups.

**Fig. 2:**
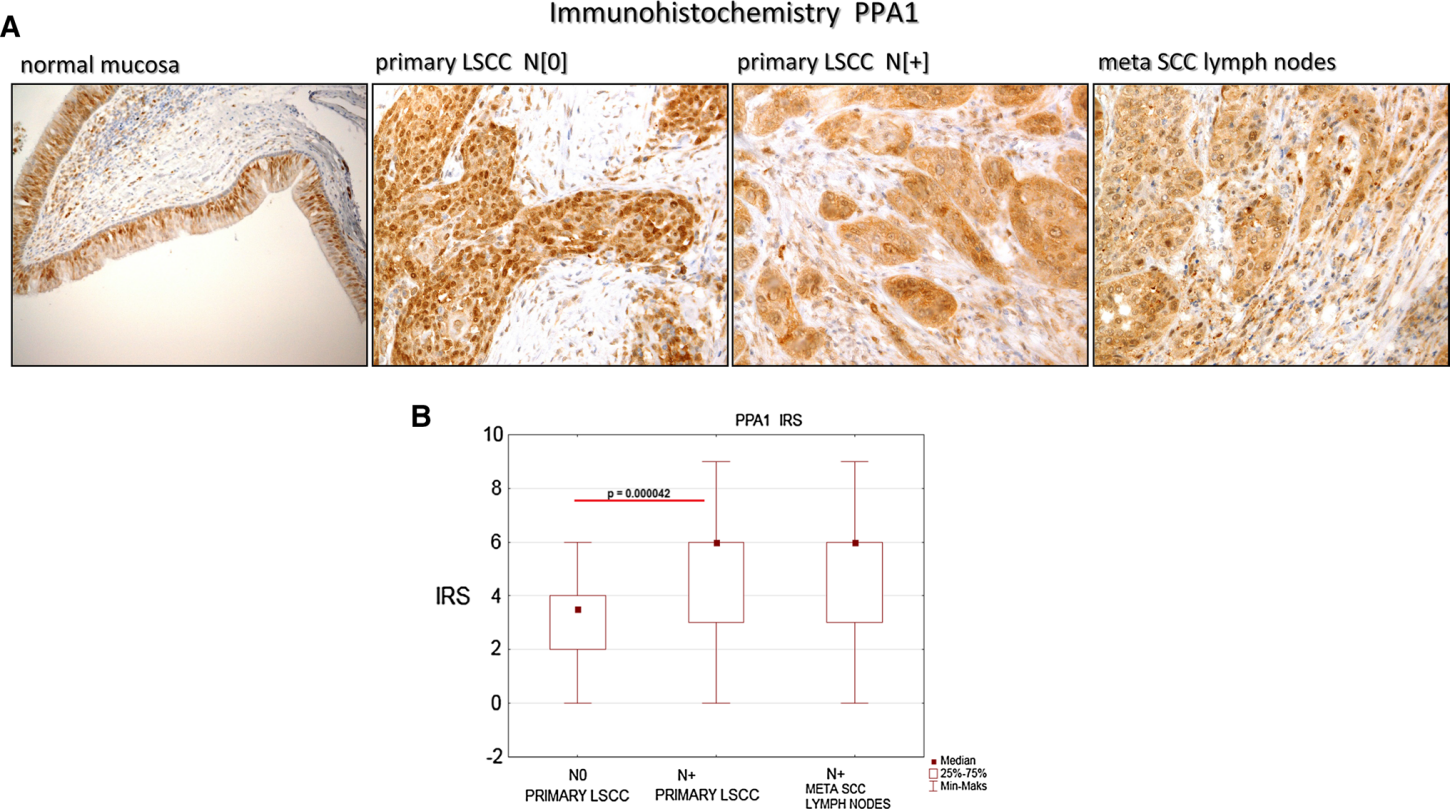
**a** The immunohistochemical staining of PPA1 in normal mucosa and laryngeal squamous cell carcinoma. Positive nuclear-cytoplasmic staining (*brown*), primary objective magnification ×10. **b** Median immunostaining of analyzed antigen in primary tumors, relative to lymph node involvement [N(0) vs. N(+)], and metastases in lymph nodes. *IRS* immunoreactive score

In contrast to the two above-mentioned proteins, PPA1 showed cytoplasmatic and nuclear localization with abundant positive cells (>90 %) in all three groups. Interestingly, we observed a shift in the fraction of cells showing combined nuclear and cytoplasmic staining [26.3 % of cells in N(0)] towards cells with cytoplasmic staining only [9.5 and 5.6 % cells with nuclear and cytoplasmic staining in N(+) and metastatic tumors, respectively]. The deprivation of cells showing both, PPA1 cytoplasmatic and nuclear staining in N(+) and metastatic tumors is reflected in the IRS score for nuclear staining that fell from three in the N(0) group to two in the N(+) and the metastatic tumor groups. Importantly, our results show a significant increase of cytoplasmic PPA1 abundance in the N(+) (IRS = 6) and metastatic tumors (IRS = 6) compared to the N(0) group (IRS = 3). Moreover, the statistically significant difference was observed between the PPA1 N(0) abundance, and PPA1 N(+) abundance (*p* = 0.000042) (Fig. 2).

According to the above presented results the increase of PPA1 protein abundance but not of ANXA5 and CALR proteins is correlated with metastasis formation. Therefore, the PPA1 protein is an interesting novel marker candidate of metastasis.

## Discussion

In this study, we aimed at the identification of differential proteins in primary tumors compared to recurrences/metastases of LSCC. Using an approach that combines a 2D electrophoresis-based protein screen in LSCC cell lines and subsequent immunohistochemical analysis of the candidate proteins in primary laryngeal cancer specimens, we identified the PPA1 protein that could possibly become a new marker for metastasis in this cancer.

The inorganic pyrophosphatase PPA1, member of the inorganic pyrophosphatase (PPase) family, is significantly up-regulated in LSCC cell lines derived from metastases compared to these derived from primary tumors. Importantly, we observed the same effect in immunohistochemically stained sections of primary LSCC tumors where sections derived from N(+) and metastases showed a significantly more intensive staining than sections derived from N(0) tumors demonstrating that the differences in cell lines are not merely a side effect of cell culturing. There was also a shift in PPA1 staining from a mixed nuclear-cytoplasmic in N(0) tumors to cytoplasmic in LSCC metastases.

In vivo PPA1 functions in the regulation of cell cycle. Hyperphosphorylation of RB1 mediated by CDKs and its resulting inactivation is required for transition from the G1 to S phase of the cell cycle (Weinberg 1995; Korenjak and Brehm 2005). In contrast, the PPA1 protein regulates RB1 dephosphorylation leading to cell cycle arrest in the G1 phase and growth inhibition (Berndt et al. 1997). The tumor suppressive function of PPA1 presented above seems contradictory to the observed elevated levels of the protein in the N(+) tumors. However, Berndt et al. demonstrated that the suppressive function of PPA1 requires the presence of wild type RB1 protein (Berndt et al. 1997) whereas deletions of the *RB1* gene is a common phenomenon in squamous cell carcinoma of the head and neck. In our manuscript from 2002 we have shown deletions of the *RB1* gene in as much as 37 % of primary and 60 % of metastatic LSCC tumors (Kujawski et al. 2002). In light of these findings, the elevated levels of the PPA1 protein together with the shift from mixed cytoplasmic-nuclear staining to cytoplasmic staining in the N(+) tumors might therefore reflect a process of PPA1 accumulation as consequence of aberrant RB1 suppressive pathway.

In addition, we show here decreasing abundance of the heat shock proteins HSP60 and HSP70 in cell lines derived from N(+) and metastases. This is intriguing, as it has been shown that heat shock proteins including HSP90 but also HSP70 protect mutated TP53 from degradation (Li et al. 2011). Together, the TP53-heat shock protein complex presents a dominant negative function in tumor cells. The observed up-regulation of heat shock proteins together with frequent TP53 mutations might result in selection advantage for cells in the primary LSCC tumors. This advantage might be no longer crucial in the recurrent or metastatic tumors that acquired additional genetic alterations.

In summary, we present here recurrent deregulations of Annexin III (ANXA3), Annexin V (ANXA5), calreticulin (CALR), heat shock protein 60 (HSP60), heat shock 70 kDa protein 9B (HSP70), inorganic pyrophosphatase (PPA1) and protein disulphide isomerase (PDIA3) in LSCC cell lines. Moreover, we show elevated PPA1 protein levels in the cytoplasm of N(+) LSCC tumors as compared to N(0) tumors. Therefore, the PPA1 protein is a potential marker candidate of metastasis in these tumors that could became useful in clinical routine examinations in the follow-up schedule.

## Electronic supplementary material

Below is the link to the electronic supplementary material.

Supplementary material 1 (XLSX 19 kb)

Supplementary material 2 (XLSX 10 kb)

Supplementary material 3 (HTML 19 kb)

Supplementary material 4 (HTML 10 kb)

Supplementary material 5 (HTML 19 kb)

Supplementary material 6 (HTML 10 kb)

Supplementary material 7 (HTML 19 kb)

Supplementary material 8 (HTML 10 kb)

Supplementary material 9 (HTML 10 kb)

### Electronic supplementary material

Below is the link to the electronic supplementary material.
