# Supplementary material for: Proteomic profiling identifies the inorganic pyrophosphatase (PPA1) protein as a potential biomarker of metastasis in laryngeal squamous cell carcinoma
Source: Amino Acids. 2016 Mar 7;48:1469–76. doi: 10.1007/s00726-016-2201-8 (PMC4875942; doi:10.1007/s00726-016-2201-8)
Supplement: Supplementary file 3 — Supplementary material 3 (HTML 19 kb) [file 726_2016_2201_MOESM3_ESM.html]

Mascot Search Results: ANXA3\_HUMAN


# MASCOT Search Results

## Protein View: ANXA3\_HUMAN

### Annexin A3 OS=Homo sapiens GN=ANXA3 PE=1 SV=3

|  |  |
| --- | --- |
| Database: | SwissProt |
| Score: | 108 |
| Expect: | 3.2e-07 |
| Nominal mass (Mr): | 36524 |
| Calculated pI: | 5.63 |
| Taxonomy: | Homo sapiens |

Sequence similarity is available as an NCBI BLAST search of ANXA3\_HUMAN against nr.

### Search parameters

|  |  |
| --- | --- |
| MS data file: | `peaklist.xml` |
| Enzyme: | Trypsin: cuts C-term side of KR unless next residue is P. |
| Fixed modifications: | Carbamidomethyl (C) |
| Variable modifications: | Oxidation (M) |
|  |  |
| --- | --- |
| Mass values searched: | 15 |
| Mass values matched: | 10 |

### Protein sequence coverage: 30%

Matched peptides shown in ***bold red***.

|  |  |  |  |  |  |
| --- | --- | --- | --- | --- | --- |
| `1` | `MASIWVGHRG` | `TVRDYPDFSP` | `SVDAEAIQKA` | `IRGIGTDEKM` | `LISILTERSN` |
| `51` | `AQRQLIVKEY` | `QAAYGKELKD` | `DLKGDLSGHF` | `EHLMVALVTP` | `PAVFDAKQLK` |
| `101` | `KSMKGAGTNE` | `DALIEILTTR` | `TSRQMKDISQ` | `AYYTVYKKSL` | `GDDISSETSG` |
| `151` | `DFRKALLTLA` | `DGRRDESLKV` | `DEHLAKQDAQ` | `ILYKAGENRW` | `GTDEDKFTEI` |
| `201` | `LCLRSFPQLK` | `LTFDEYRNIS` | `QKDIVDSIKG` | `ELSGHFEDLL` | `LAIVNCVRNT` |
| `251` | `PAFLAERLHR` | `ALKGIGTDEF` | `TLNRIMVSRS` | `EIDLLDIRTE` | `FKKHYGYSLY` |
| `301` | `SAIKSDTSGD` | `YEITLLKICG` | `GDD` |  |  |

Unformatted sequence string: 323 residues (for pasting into other applications).

Residue Number

Increasing Mass

Decreasing Mass

| Start | – | End | Observed | Mr(expt) | Mr(calc) | Delta | M | Peptide |
| --- | --- | --- | --- | --- | --- | --- | --- | --- |
| 40 | – | 48 | 1075.5819 | 1074.5746 | 1074.6107 | -0.0361 | 0 | K.MLISILTER.S |
| 105 | – | 120 | 1673.8484 | 1672.8411 | 1672.8632 | -0.0220 | 0 | K.GAGTNEDALIEILTTR.T |
| 139 | – | 153 | 1585.6853 | 1584.6780 | 1584.6904 | -0.0123 | 0 | K.SLGDDISSETSGDFR.K |
| 155 | – | 163 | 929.4979 | 928.4906 | 928.5342 | -0.0436 | 0 | K.ALLTLADGR.R |
| 190 | – | 204 | 1882.8633 | 1881.8560 | 1881.8931 | -0.0371 | 1 | R.WGTDEDKFTEILCLR.S |
| 197 | – | 204 | 1051.5292 | 1050.5219 | 1050.5532 | -0.0313 | 0 | K.FTEILCLR.S |
| 211 | – | 217 | 943.3843 | 942.3770 | 942.4447 | -0.0677 | 0 | K.LTFDEYR.N |
| 249 | – | 257 | 1018.4636 | 1017.4563 | 1017.5243 | -0.0680 | 0 | R.NTPAFLAER.L |
| 264 | – | 274 | 1222.5760 | 1221.5688 | 1221.5990 | -0.0302 | 0 | K.GIGTDEFTLNR.I |
| 280 | – | 288 | 1073.5630 | 1072.5557 | 1072.5764 | -0.0207 | 0 | R.SEIDLLDIR.T |

`No match to: 854.9537, 856.4859, 870.5244, 967.4593, 1236.5763`

---

```
AC   P12429; B2R9W6; Q6LET2;
DT   01-OCT-1989, integrated into UniProtKB/Swiss-Prot.
DT   23-JAN-2007, sequence version 3.
DT   09-DEC-2015, entry version 167.
DE   RecName: Full=Annexin A3;
DE   AltName: Full=35-alpha calcimedin;
DE   AltName: Full=Annexin III;
DE   AltName: Full=Annexin-3;
DE   AltName: Full=Inositol 1,2-cyclic phosphate 2-phosphohydrolase;
DE   AltName: Full=Lipocortin III;
DE   AltName: Full=Placental anticoagulant protein III;
DE            Short=PAP-III;
GN   Name=ANXA3; Synonyms=ANX3;
OS   Homo sapiens (Human).
OC   Eukaryota; Metazoa; Chordata; Craniata; Vertebrata; Euteleostomi;
OC   Mammalia; Eutheria; Euarchontoglires; Primates; Haplorrhini;
OC   Catarrhini; Hominidae; Homo.
OX   NCBI_TaxID=9606;
RN   [1]
RP   NUCLEOTIDE SEQUENCE [MRNA].
RX   PubMed=2968983;
RA   Pepinsky R.B., Tizard R., Mattaliano R.J., Sinclair L.K., Miller G.T.,
RA   Browning J.L., Chow E.P., Burne C., Huang K.-S., Pratt D., Wachter L.,
RA   Hession C., Frey A.Z., Wallner B.P.;
RT   "Five distinct calcium and phospholipid binding proteins share
RT   homology with lipocortin I.";
RL   J. Biol. Chem. 263:10799-10811(1988).
RN   [2]
RP   NUCLEOTIDE SEQUENCE [MRNA].
RX   PubMed=1830024; DOI=10.1016/0888-7543(91)90330-H;
RA   Tait J.F., Frankenberry D.A., Miao C.H., Killary A.M., Adler D.A.,
RA   Disteche C.M.;
RT   "Chromosomal localization of the human annexin III (ANX3) gene.";
RL   Genomics 10:441-448(1991).
RN   [3]
RP   NUCLEOTIDE SEQUENCE [GENOMIC DNA].
RX   PubMed=8276419; DOI=10.1006/geno.1993.1428;
RA   Tait J.F., Smith C., Xu L., Cookson B.T.;
RT   "Structure and polymorphisms of the human annexin III (ANX3) gene.";
RL   Genomics 18:79-86(1993).
RN   [4]
RP   NUCLEOTIDE SEQUENCE [LARGE SCALE MRNA].
RA   Ebert L., Schick M., Neubert P., Schatten R., Henze S., Korn B.;
RT   "Cloning of human full open reading frames in Gateway(TM) system entry
RT   vector (pDONR201).";
RL   Submitted (MAY-2004) to the EMBL/GenBank/DDBJ databases.
RN   [5]
RP   NUCLEOTIDE SEQUENCE [LARGE SCALE MRNA].
RC   TISSUE=Skeletal muscle;
RX   PubMed=14702039; DOI=10.1038/ng1285;
RA   Ota T., Suzuki Y., Nishikawa T., Otsuki T., Sugiyama T., Irie R.,
RA   Wakamatsu A., Hayashi K., Sato H., Nagai K., Kimura K., Makita H.,
RA   Sekine M., Obayashi M., Nishi T., Shibahara T., Tanaka T., Ishii S.,
RA   Yamamoto J., Saito K., Kawai Y., Isono Y., Nakamura Y., Nagahari K.,
RA   Murakami K., Yasuda T., Iwayanagi T., Wagatsuma M., Shiratori A.,
RA   Sudo H., Hosoiri T., Kaku Y., Kodaira H., Kondo H., Sugawara M.,
RA   Takahashi M., Kanda K., Yokoi T., Furuya T., Kikkawa E., Omura Y.,
RA   Abe K., Kamihara K., Katsuta N., Sato K., Tanikawa M., Yamazaki M.,
RA   Ninomiya K., Ishibashi T., Yamashita H., Murakawa K., Fujimori K.,
RA   Tanai H., Kimata M., Watanabe M., Hiraoka S., Chiba Y., Ishida S.,
RA   Ono Y., Takiguchi S., Watanabe S., Yosida M., Hotuta T., Kusano J.,
RA   Kanehori K., Takahashi-Fujii A., Hara H., Tanase T.-O., Nomura Y.,
RA   Togiya S., Komai F., Hara R., Takeuchi K., Arita M., Imose N.,
RA   Musashino K., Yuuki H., Oshima A., Sasaki N., Aotsuka S.,
RA   Yoshikawa Y., Matsunawa H., Ichihara T., Shiohata N., Sano S.,
RA   Moriya S., Momiyama H., Satoh N., Takami S., Terashima Y., Suzuki O.,
RA   Nakagawa S., Senoh A., Mizoguchi H., Goto Y., Shimizu F., Wakebe H.,
RA   Hishigaki H., Watanabe T., Sugiyama A., Takemoto M., Kawakami B.,
RA   Yamazaki M., Watanabe K., Kumagai A., Itakura S., Fukuzumi Y.,
RA   Fujimori Y., Komiyama M., Tashiro H., Tanigami A., Fujiwara T.,
RA   Ono T., Yamada K., Fujii Y., Ozaki K., Hirao M., Ohmori Y.,
RA   Kawabata A., Hikiji T., Kobatake N., Inagaki H., Ikema Y., Okamoto S.,
RA   Okitani R., Kawakami T., Noguchi S., Itoh T., Shigeta K., Senba T.,
RA   Matsumura K., Nakajima Y., Mizuno T., Morinaga M., Sasaki M.,
RA   Togashi T., Oyama M., Hata H., Watanabe M., Komatsu T.,
RA   Mizushima-Sugano J., Satoh T., Shirai Y., Takahashi Y., Nakagawa K.,
RA   Okumura K., Nagase T., Nomura N., Kikuchi H., Masuho Y., Yamashita R.,
RA   Nakai K., Yada T., Nakamura Y., Ohara O., Isogai T., Sugano S.;
RT   "Complete sequencing and characterization of 21,243 full-length human
RT   cDNAs.";
RL   Nat. Genet. 36:40-45(2004).
RN   [6]
RP   NUCLEOTIDE SEQUENCE [LARGE SCALE GENOMIC DNA].
RA   Mural R.J., Istrail S., Sutton G.G., Florea L., Halpern A.L.,
RA   Mobarry C.M., Lippert R., Walenz B., Shatkay H., Dew I., Miller J.R.,
RA   Flanigan M.J., Edwards N.J., Bolanos R., Fasulo D., Halldorsson B.V.,
RA   Hannenhalli S., Turner R., Yooseph S., Lu F., Nusskern D.R.,
RA   Shue B.C., Zheng X.H., Zhong F., Delcher A.L., Huson D.H.,
RA   Kravitz S.A., Mouchard L., Reinert K., Remington K.A., Clark A.G.,
RA   Waterman M.S., Eichler E.E., Adams M.D., Hunkapiller M.W., Myers E.W.,
RA   Venter J.C.;
RL   Submitted (JUL-2005) to the EMBL/GenBank/DDBJ databases.
RN   [7]
RP   NUCLEOTIDE SEQUENCE [LARGE SCALE MRNA].
RC   TISSUE=Cervix;
RX   PubMed=15489334; DOI=10.1101/gr.2596504;
RG   The MGC Project Team;
RT   "The status, quality, and expansion of the NIH full-length cDNA
RT   project: the Mammalian Gene Collection (MGC).";
RL   Genome Res. 14:2121-2127(2004).
RN   [8]
RP   PROTEIN SEQUENCE OF 2-8.
RC   TISSUE=Platelet;
RX   PubMed=12665801; DOI=10.1038/nbt810;
RA   Gevaert K., Goethals M., Martens L., Van Damme J., Staes A.,
RA   Thomas G.R., Vandekerckhove J.;
RT   "Exploring proteomes and analyzing protein processing by mass
RT   spectrometric identification of sorted N-terminal peptides.";
RL   Nat. Biotechnol. 21:566-569(2003).
RN   [9]
RP   PROTEIN SEQUENCE OF 2-9; 40-48; 105-120; 155-163; 249-257; 264-274 AND
RP   280-288, CLEAVAGE OF INITIATOR METHIONINE, ACETYLATION AT ALA-2, AND
RP   IDENTIFICATION BY MASS SPECTROMETRY.
RC   TISSUE=Colon carcinoma;
RA   Bienvenut W.V., Heiserich L., Gottlieb E.;
RL   Submitted (MAR-2008) to UniProtKB.
RN   [10]
RP   PROTEIN SEQUENCE OF 41-102 AND 126-138.
RX   PubMed=2159184; DOI=10.1126/science.2159184;
RA   Ross T.S., Tait J.F., Majerus P.W.;
RT   "Identity of inositol 1,2-cyclic phosphate 2-phosphohydrolase with
RT   lipocortin III.";
RL   Science 248:605-607(1990).
RN   [11]
RP   PROTEIN SEQUENCE OF 41-79; 85-88; 104-119; 126-150 AND 217-323.
RX   PubMed=2975506; DOI=10.1021/bi00417a011;
RA   Tait J.F., Sakata M., McMullen B.A., Miao C.H., Funakoshi T.,
RA   Hendrickson L.E., Fujikawa K.;
RT   "Placental anticoagulant proteins: isolation and comparative
RT   characterization four members of the lipocortin family.";
RL   Biochemistry 27:6268-6276(1988).
RN   [12]
RP   PROTEIN SEQUENCE OF 42-55; 74-82; 105-126; 155-169; 177-209; 264-274
RP   AND 305-315, AND CALCIUM-DEPENDENT BINDING TO PHOSPHOLIPIDS.
RX   PubMed=2138632; DOI=10.1172/JCI114537;
RA   Ernst J.D., Hoye E., Blackwood R.A., Jaye D.;
RT   "Purification and characterization of an abundant cytosolic protein
RT   from human neutrophils that promotes Ca2(+)-dependent aggregation of
RT   isolated specific granules.";
RL   J. Clin. Invest. 85:1065-1071(1990).
RN   [13]
RP   IDENTIFICATION BY MASS SPECTROMETRY [LARGE SCALE ANALYSIS].
RX   PubMed=21269460; DOI=10.1186/1752-0509-5-17;
RA   Burkard T.R., Planyavsky M., Kaupe I., Breitwieser F.P.,
RA   Buerckstuemmer T., Bennett K.L., Superti-Furga G., Colinge J.;
RT   "Initial characterization of the human central proteome.";
RL   BMC Syst. Biol. 5:17-17(2011).
RN   [14]
RP   X-RAY CRYSTALLOGRAPHY (1.8 ANGSTROMS).
RX   PubMed=8639653; DOI=10.1021/bi952092o;
RA   Favier-Perron B., Lewit-Bentley A., Russo-Marie F.;
RT   "The high-resolution crystal structure of human annexin III shows
RT   subtle differences with annexin V.";
RL   Biochemistry 35:1740-1744(1996).
RN   [15]
RP   VARIANTS ASN-19; ASN-219; LEU-251 AND SER-291.
RX   PubMed=10391209; DOI=10.1038/10290;
RA   Cargill M., Altshuler D., Ireland J., Sklar P., Ardlie K., Patil N.,
RA   Shaw N., Lane C.R., Lim E.P., Kalyanaraman N., Nemesh J., Ziaugra L.,
RA   Friedland L., Rolfe A., Warrington J., Lipshutz R., Daley G.Q.,
RA   Lander E.S.;
RT   "Characterization of single-nucleotide polymorphisms in coding regions
RT   of human genes.";
RL   Nat. Genet. 22:231-238(1999).
RN   [16]
RP   ERRATUM.
RA   Cargill M., Altshuler D., Ireland J., Sklar P., Ardlie K., Patil N.,
RA   Shaw N., Lane C.R., Lim E.P., Kalyanaraman N., Nemesh J., Ziaugra L.,
RA   Friedland L., Rolfe A., Warrington J., Lipshutz R., Daley G.Q.,
RA   Lander E.S.;
RL   Nat. Genet. 23:373-373(1999).
CC   -!- FUNCTION: Inhibitor of phospholipase A2, also possesses anti-
CC       coagulant properties. Also cleaves the cyclic bond of inositol
CC       1,2-cyclic phosphate to form inositol 1-phosphate.
CC   -!- DOMAIN: A pair of annexin repeats may form one binding site for
CC       calcium and phospholipid.
CC   -!- SIMILARITY: Belongs to the annexin family. {ECO:0000305}.
CC   -!- SIMILARITY: Contains 4 annexin repeats. {ECO:0000305}.
DR   EMBL; M20560; AAA59496.1; -; mRNA.
DR   EMBL; M63310; AAA52284.1; -; mRNA.
DR   EMBL; L20591; AAA16713.1; -; Genomic_DNA.
DR   EMBL; CR407648; CAG28576.1; -; mRNA.
DR   EMBL; AK313945; BAG36663.1; -; mRNA.
DR   EMBL; CH471057; EAX05822.1; -; Genomic_DNA.
DR   EMBL; BC000871; AAH00871.1; -; mRNA.
DR   CCDS; CCDS3584.1; -.
DR   PIR; A47658; LUHU3.
DR   RefSeq; NP_005130.1; NM_005139.2.
DR   UniGene; Hs.480042; -.
DR   PDB; 1AII; X-ray; 1.95 A; A=1-323.
DR   PDB; 1AXN; X-ray; 1.78 A; A=2-323.
DR   PDBsum; 1AII; -.
DR   PDBsum; 1AXN; -.
DR   ProteinModelPortal; P12429; -.
DR   SMR; P12429; 2-323.
DR   BioGrid; 106803; 13.
DR   IntAct; P12429; 6.
DR   MINT; MINT-4998835; -.
DR   STRING; 9606.ENSP00000264908; -.
DR   PhosphoSite; P12429; -.
DR   BioMuta; ANXA3; -.
DR   DMDM; 113954; -.
DR   OGP; P12429; -.
DR   SWISS-2DPAGE; P12429; -.
DR   MaxQB; P12429; -.
DR   PaxDb; P12429; -.
DR   PRIDE; P12429; -.
DR   DNASU; 306; -.
DR   Ensembl; ENST00000264908; ENSP00000264908; ENSG00000138772.
DR   GeneID; 306; -.
DR   KEGG; hsa:306; -.
DR   UCSC; uc003hld.3; human.
DR   CTD; 306; -.
DR   GeneCards; ANXA3; -.
DR   HGNC; HGNC:541; ANXA3.
DR   HPA; HPA013398; -.
DR   HPA; HPA013431; -.
DR   MIM; 106490; gene.
DR   neXtProt; NX_P12429; -.
DR   PharmGKB; PA24831; -.
DR   eggNOG; KOG0819; Eukaryota.
DR   eggNOG; ENOG410XPUN; LUCA.
DR   GeneTree; ENSGT00760000118972; -.
DR   HOGENOM; HOG000158803; -.
DR   HOVERGEN; HBG061815; -.
DR   InParanoid; P12429; -.
DR   KO; K17089; -.
DR   OMA; VNCVRNT; -.
DR   OrthoDB; EOG74XS72; -.
DR   PhylomeDB; P12429; -.
DR   TreeFam; TF105452; -.
DR   ChiTaRS; ANXA3; human.
DR   EvolutionaryTrace; P12429; -.
DR   GeneWiki; Annexin_A3; -.
DR   GenomeRNAi; 306; -.
DR   NextBio; 1235; -.
DR   PRO; PR:P12429; -.
DR   Proteomes; UP000005640; Chromosome 4.
DR   Bgee; P12429; -.
DR   CleanEx; HS_ANXA3; -.
DR   ExpressionAtlas; P12429; baseline and differential.
DR   Genevisible; P12429; HS.
DR   GO; GO:0030424; C:axon; IEA:Ensembl.
DR   GO; GO:0005737; C:cytoplasm; IDA:UniProtKB.
DR   GO; GO:0030425; C:dendrite; IEA:Ensembl.
DR   GO; GO:0070062; C:extracellular exosome; IDA:UniProtKB.
DR   GO; GO:0016020; C:membrane; IDA:UniProtKB.
DR   GO; GO:0043025; C:neuronal cell body; IEA:Ensembl.
DR   GO; GO:0030670; C:phagocytic vesicle membrane; IDA:UniProtKB.
DR   GO; GO:0005886; C:plasma membrane; IDA:UniProtKB.
DR   GO; GO:0042581; C:specific granule; IDA:UniProtKB.
DR   GO; GO:0005509; F:calcium ion binding; IEA:InterPro.
DR   GO; GO:0005544; F:calcium-dependent phospholipid binding; IDA:UniProtKB.
DR   GO; GO:0048306; F:calcium-dependent protein binding; IPI:AgBase.
DR   GO; GO:0019834; F:phospholipase A2 inhibitor activity; IEA:UniProtKB-KW.
DR   GO; GO:0042742; P:defense response to bacterium; IDA:UniProtKB.
DR   GO; GO:0021766; P:hippocampus development; IEA:Ensembl.
DR   GO; GO:0043312; P:neutrophil degranulation; IDA:UniProtKB.
DR   GO; GO:0031100; P:organ regeneration; IEA:Ensembl.
DR   GO; GO:0006909; P:phagocytosis; IDA:UniProtKB.
DR   GO; GO:0045766; P:positive regulation of angiogenesis; IDA:UniProtKB.
DR   GO; GO:0051054; P:positive regulation of DNA metabolic process; IEA:Ensembl.
DR   GO; GO:0010595; P:positive regulation of endothelial cell migration; IDA:UniProtKB.
DR   GO; GO:0051091; P:positive regulation of sequence-specific DNA binding transcription factor activity; IDA:UniProtKB.
DR   GO; GO:0051384; P:response to glucocorticoid; IEA:Ensembl.
DR   GO; GO:0070848; P:response to growth factor; IEA:Ensembl.
DR   Gene3D; 1.10.220.10; -; 4.
DR   InterPro; IPR001464; Annexin.
DR   InterPro; IPR018502; Annexin_repeat.
DR   InterPro; IPR018252; Annexin_repeat_CS.
DR   InterPro; IPR002390; AnnexinIII.
DR   PANTHER; PTHR10502:SF25; PTHR10502:SF25; 1.
DR   Pfam; PF00191; Annexin; 4.
DR   PRINTS; PR00196; ANNEXIN.
DR   PRINTS; PR00199; ANNEXINIII.
DR   SMART; SM00335; ANX; 4.
DR   PROSITE; PS00223; ANNEXIN; 4.
PE   1: Evidence at protein level;
KW   3D-structure; Acetylation; Annexin; Calcium;
KW   Calcium/phospholipid-binding; Complete proteome;
KW   Direct protein sequencing; Phospholipase A2 inhibitor; Phosphoprotein;
KW   Polymorphism; Reference proteome; Repeat.
FT   INIT_MET      1      1       Removed. {ECO:0000269|PubMed:12665801,
FT                                ECO:0000269|Ref.9}.
FT   CHAIN         2    323       Annexin A3.
FT                                /FTId=PRO_0000067477.
FT   REPEAT       27     87       Annexin 1.
FT   REPEAT       99    159       Annexin 2.
FT   REPEAT      183    243       Annexin 3.
FT   REPEAT      258    318       Annexin 4.
FT   MOD_RES       2      2       N-acetylalanine. {ECO:0000269|Ref.9}.
FT   MOD_RES     267    267       Phosphothreonine.
FT                                {ECO:0000250|UniProtKB:P14669}.
FT   VARIANT      19     19       S -> N (in dbSNP:rs5951).
FT                                {ECO:0000269|PubMed:10391209}.
FT                                /FTId=VAR_013914.
FT   VARIANT     219    219       I -> N (in dbSNP:rs5948).
FT                                {ECO:0000269|PubMed:10391209}.
FT                                /FTId=VAR_013915.
FT   VARIANT     251    251       P -> L (in dbSNP:rs5949).
FT                                {ECO:0000269|PubMed:10391209}.
FT                                /FTId=VAR_013916.
FT   VARIANT     291    291       F -> S (in dbSNP:rs5941).
FT                                {ECO:0000269|PubMed:10391209}.
FT                                /FTId=VAR_013917.
FT   CONFLICT     35     35       G -> R (in Ref. 4; CAG28576).
FT                                {ECO:0000305}.
FT   CONFLICT    146    146       S -> G (in Ref. 11; AA sequence).
FT                                {ECO:0000305}.
FT   CONFLICT    294    294       H -> R (in Ref. 11; AA sequence).
FT                                {ECO:0000305}.
FT   HELIX        20     31       {ECO:0000244|PDB:1AXN}.
FT   STRAND       32     35       {ECO:0000244|PDB:1AXN}.
FT   HELIX        38     45       {ECO:0000244|PDB:1AXN}.
FT   HELIX        50     64       {ECO:0000244|PDB:1AXN}.
FT   HELIX        68     75       {ECO:0000244|PDB:1AXN}.
FT   HELIX        78     88       {ECO:0000244|PDB:1AXN}.
FT   HELIX        91    103       {ECO:0000244|PDB:1AXN}.
FT   STRAND      105    107       {ECO:0000244|PDB:1AXN}.
FT   HELIX       110    119       {ECO:0000244|PDB:1AXN}.
FT   HELIX       122    136       {ECO:0000244|PDB:1AXN}.
FT   HELIX       140    147       {ECO:0000244|PDB:1AXN}.
FT   HELIX       150    160       {ECO:0000244|PDB:1AXN}.
FT   HELIX       172    185       {ECO:0000244|PDB:1AXN}.
FT   TURN        186    188       {ECO:0000244|PDB:1AXN}.
FT   STRAND      189    191       {ECO:0000244|PDB:1AXN}.
FT   HELIX       194    203       {ECO:0000244|PDB:1AXN}.
FT   HELIX       206    220       {ECO:0000244|PDB:1AXN}.
FT   HELIX       224    231       {ECO:0000244|PDB:1AXN}.
FT   HELIX       234    262       {ECO:0000244|PDB:1AXN}.
FT   STRAND      263    266       {ECO:0000244|PDB:1AXN}.
FT   HELIX       269    279       {ECO:0000244|PDB:1AXN}.
FT   TURN        280    283       {ECO:0000244|PDB:1AXN}.
FT   HELIX       284    295       {ECO:0000244|PDB:1AXN}.
FT   HELIX       299    306       {ECO:0000244|PDB:1AXN}.
FT   HELIX       309    319       {ECO:0000244|PDB:1AXN}.
SQ   SEQUENCE   323 AA;  36375 MW;  4128C715491FC132 CRC64;
     MASIWVGHRG TVRDYPDFSP SVDAEAIQKA IRGIGTDEKM LISILTERSN AQRQLIVKEY
     QAAYGKELKD DLKGDLSGHF EHLMVALVTP PAVFDAKQLK KSMKGAGTNE DALIEILTTR
     TSRQMKDISQ AYYTVYKKSL GDDISSETSG DFRKALLTLA DGRRDESLKV DEHLAKQDAQ
     ILYKAGENRW GTDEDKFTEI LCLRSFPQLK LTFDEYRNIS QKDIVDSIKG ELSGHFEDLL
     LAIVNCVRNT PAFLAERLHR ALKGIGTDEF TLNRIMVSRS EIDLLDIRTE FKKHYGYSLY
     SAIKSDTSGD YEITLLKICG GDD
```

|  |
| --- |
| **Mascot:** http://www.matrixscience.com/ |
