# Supplementary material for: Proteomic profiling identifies the inorganic pyrophosphatase (PPA1) protein as a potential biomarker of metastasis in laryngeal squamous cell carcinoma
Source: Amino Acids. 2016 Mar 7;48:1469–76. doi: 10.1007/s00726-016-2201-8 (PMC4875942; doi:10.1007/s00726-016-2201-8)
Supplement: Supplementary file 4 — Supplementary material 4 (HTML 10 kb) [file 726_2016_2201_MOESM4_ESM.html]

Mascot Search Results: ANXA5\_HUMAN


# MASCOT Search Results

## Protein View: ANXA5\_HUMAN

### Annexin A5 OS=Homo sapiens GN=ANXA5 PE=1 SV=2

|  |  |
| --- | --- |
| Database: | SwissProt |
| Score: | 163 |
| Expect: | 1e-12 |
| Nominal mass (Mr): | 35971 |
| Calculated pI: | 4.94 |
| Taxonomy: | Homo sapiens |

Sequence similarity is available as an NCBI BLAST search of ANXA5\_HUMAN against nr.

### Search parameters

|  |  |
| --- | --- |
| MS data file: | `peaklist.xml` |
| Enzyme: | Trypsin: cuts C-term side of KR unless next residue is P. |
| Fixed modifications: | Carbamidomethyl (C) |
| Variable modifications: | Oxidation (M) |
|  |  |
| --- | --- |
| Mass values searched: | 30 |
| Mass values matched: | 16 |

### Protein sequence coverage: 51%

Matched peptides shown in ***bold red***.

|  |  |  |  |  |  |
| --- | --- | --- | --- | --- | --- |
| `1` | `MAQVLRGTVT` | `DFPGFDERAD` | `AETLRKAMKG` | `LGTDEESILT` | `LLTSRSNAQR` |
| `51` | `QEISAAFKTL` | `FGRDLLDDLK` | `SELTGKFEKL` | `IVALMKPSRL` | `YDAYELKHAL` |
| `101` | `KGAGTNEKVL` | `TEIIASRTPE` | `ELRAIKQVYE` | `EEYGSSLEDD` | `VVGDTSGYYQ` |
| `151` | `RMLVVLLQAN` | `RDPDAGIDEA` | `QVEQDAQALF` | `QAGELKWGTD` | `EEKFITIFGT` |
| `201` | `RSVSHLRKVF` | `DKYMTISGFQ` | `IEETIDRETS` | `GNLEQLLLAV` | `VKSIRSIPAY` |
| `251` | `LAETLYYAMK` | `GAGTDDHTLI` | `RVMVSRSEID` | `LFNIRKEFRK` | `NFATSLYSMI` |
| `301` | `KGDTSGDYKK` | `ALLLLCGEDD` |  |  |  |

Unformatted sequence string: 320 residues (for pasting into other applications).

Residue Number

Increasing Mass

Decreasing Mass

| Start | – | End | Observed | Mr(expt) | Mr(calc) | Delta | M | Peptide |
| --- | --- | --- | --- | --- | --- | --- | --- | --- |
| 7 | – | 18 | 1340.8116 | 1339.8044 | 1339.6045 | 0.1999 | 0 | R.GTVTDFPGFDER.A |
| 30 | – | 45 | 1705.0941 | 1704.0868 | 1703.8941 | 0.1927 | 0 | K.GLGTDEESILTLLTSR.S |
| 51 | – | 58 | 893.6360 | 892.6288 | 892.4654 | 0.1634 | 0 | R.QEISAAFK.T |
| 90 | – | 97 | 1014.6841 | 1013.6768 | 1013.5069 | 0.1699 | 0 | R.LYDAYELK.H |
| 109 | – | 117 | 1001.7794 | 1000.7721 | 1000.5917 | 0.1804 | 0 | K.VLTEIIASR.T |
| 152 | – | 161 | 1172.8680 | 1171.8608 | 1171.6747 | 0.1861 | 0 | R.MLVVLLQANR.D + Oxidation (M) |
| 162 | – | 186 | 2658.5059 | 2657.4986 | 2657.2457 | 0.2529 | 0 | R.DPDAGIDEAQVEQDAQALFQAGELK.W |
| 187 | – | 193 | 864.5645 | 863.5572 | 863.3661 | 0.1911 | 0 | K.WGTDEEK.F |
| 194 | – | 201 | 954.7177 | 953.7104 | 953.5335 | 0.1769 | 0 | K.FITIFGTR.S |
| 213 | – | 227 | 1803.0597 | 1802.0524 | 1801.8556 | 0.1968 | 0 | K.YMTISGFQIEETIDR.E |
| 213 | – | 227 | 1819.0868 | 1818.0795 | 1817.8506 | 0.2290 | 0 | K.YMTISGFQIEETIDR.E + Oxidation (M) |
| 228 | – | 242 | 1614.1041 | 1613.0968 | 1612.9036 | 0.1933 | 0 | R.ETSGNLEQLLLAVVK.S |
| 261 | – | 271 | 1155.7874 | 1154.7801 | 1154.5680 | 0.2121 | 0 | K.GAGTDDHTLIR.V |
| 277 | – | 285 | 1106.7662 | 1105.7590 | 1105.5768 | 0.1822 | 0 | R.SEIDLFNIR.K |
| 291 | – | 301 | 1274.8220 | 1273.8147 | 1273.6376 | 0.1771 | 0 | K.NFATSLYSMIK.G |
| 291 | – | 301 | 1290.8225 | 1289.8152 | 1289.6326 | 0.1827 | 0 | K.NFATSLYSMIK.G + Oxidation (M) |

`No match to: 861.2621, 870.7573, 976.7216, 1003.6847, 1128.7560, 1177.7853, 1296.8179, 1354.8590, 1362.8049, 1636.0817, 1719.1182, 1767.4148, 2871.5742, 2889.8376`

---

```
AC   P08758; D3DNW7; Q6FHB3; Q6FI16; Q8WV69; Q9UDH9;
DT   01-NOV-1988, integrated into UniProtKB/Swiss-Prot.
DT   23-JAN-2007, sequence version 2.
DT   09-DEC-2015, entry version 185.
DE   RecName: Full=Annexin A5;
DE   AltName: Full=Anchorin CII;
DE   AltName: Full=Annexin V;
DE   AltName: Full=Annexin-5;
DE   AltName: Full=Calphobindin I;
DE            Short=CBP-I;
DE   AltName: Full=Endonexin II;
DE   AltName: Full=Lipocortin V;
DE   AltName: Full=Placental anticoagulant protein 4;
DE            Short=PP4;
DE   AltName: Full=Placental anticoagulant protein I;
DE            Short=PAP-I;
DE   AltName: Full=Thromboplastin inhibitor;
DE   AltName: Full=Vascular anticoagulant-alpha;
DE            Short=VAC-alpha;
GN   Name=ANXA5; Synonyms=ANX5, ENX2, PP4;
OS   Homo sapiens (Human).
OC   Eukaryota; Metazoa; Chordata; Craniata; Vertebrata; Euteleostomi;
OC   Mammalia; Eutheria; Euarchontoglires; Primates; Haplorrhini;
OC   Catarrhini; Hominidae; Homo.
OX   NCBI_TaxID=9606;
RN   [1]
RP   NUCLEOTIDE SEQUENCE [MRNA].
RX   PubMed=2964863; DOI=10.1021/bi00399a011;
RA   Funakoshi T., Hendrickson L.E., McMullen B.A., Fujikawa K.;
RT   "Primary structure of human placental anticoagulant protein.";
RL   Biochemistry 26:8087-8092(1987).
RN   [2]
RP   NUCLEOTIDE SEQUENCE [MRNA], AND PROTEIN SEQUENCE OF 2-320.
RX   PubMed=2963810;
RA   Iwasaki A., Suda M., Nakao H., Nagoya T., Saino Y., Arai K.,
RA   Mizoguchi T., Sato F., Yoshizaki H., Hirata M., Miyata T., Shidara Y.,
RA   Murata M., Maki M.;
RT   "Structure and expression of cDNA for an inhibitor of blood
RT   coagulation isolated from human placenta: a new lipocortin-like
RT   protein.";
RL   J. Biochem. 102:1261-1273(1987).
RN   [3]
RP   NUCLEOTIDE SEQUENCE [MRNA], AND PARTIAL PROTEIN SEQUENCE.
RX   PubMed=2455636; DOI=10.1111/j.1432-1033.1988.tb14139.x;
RA   Maurer-Fogy I., Reutelingsperger C.P.M., Pieters J., Bodo G.,
RA   Stratowa C., Hauptmann R.;
RT   "Cloning and expression of cDNA for human vascular anticoagulant, a
RT   Ca2+-dependent phospholipid-binding protein.";
RL   Eur. J. Biochem. 174:585-592(1988).
RN   [4]
RP   NUCLEOTIDE SEQUENCE [MRNA].
RX   PubMed=2967291;
RA   Kaplan R., Jaye M., Burgess W.H., Schlaepfer D.D., Haigler H.T.;
RT   "Cloning and expression of cDNA for human endonexin II, a Ca2+ and
RT   phospholipid binding protein.";
RL   J. Biol. Chem. 263:8037-8043(1988).
RN   [5]
RP   NUCLEOTIDE SEQUENCE [MRNA].
RX   PubMed=2968983;
RA   Pepinsky R.B., Tizard R., Mattaliano R.J., Sinclair L.K., Miller G.T.,
RA   Browning J.L., Chow E.P., Burne C., Huang K.-S., Pratt D., Wachter L.,
RA   Hession C., Frey A.Z., Wallner B.P.;
RT   "Five distinct calcium and phospholipid binding proteins share
RT   homology with lipocortin I.";
RL   J. Biol. Chem. 263:10799-10811(1988).
RN   [6]
RP   NUCLEOTIDE SEQUENCE [MRNA].
RX   PubMed=2967495; DOI=10.1073/pnas.85.11.3708;
RA   Grundmann U., Abel K.-J., Bohn H., Loebermann H., Lottspeich F.,
RA   Kuepper H.;
RT   "Characterization of cDNA encoding human placental anticoagulant
RT   protein (PP4): homology with the lipocortin family.";
RL   Proc. Natl. Acad. Sci. U.S.A. 85:3708-3712(1988).
RN   [7]
RP   NUCLEOTIDE SEQUENCE [GENOMIC DNA].
RC   TISSUE=Lung;
RX   PubMed=7958998; DOI=10.1016/0378-1119(94)90157-0;
RA   Fernandez M.-P., Morgan R.O., Fernandez M.R., Carcedo M.-T.;
RT   "The gene encoding human annexin V has a TATA-less promoter with a
RT   high G+C content.";
RL   Gene 149:253-260(1994).
RN   [8]
RP   NUCLEOTIDE SEQUENCE [GENOMIC DNA].
RX   PubMed=8034319; DOI=10.1006/geno.1994.1201;
RA   Cookson B.T., Engelhardt S., Smith C., Bamford H.A., Prochazka M.,
RA   Tait J.F.;
RT   "Organization of the human annexin V (ANX5) gene.";
RL   Genomics 20:463-467(1994).
RN   [9]
RP   NUCLEOTIDE SEQUENCE [LARGE SCALE MRNA].
RC   TISSUE=Neuroblastoma;
RX   PubMed=14702039; DOI=10.1038/ng1285;
RA   Ota T., Suzuki Y., Nishikawa T., Otsuki T., Sugiyama T., Irie R.,
RA   Wakamatsu A., Hayashi K., Sato H., Nagai K., Kimura K., Makita H.,
RA   Sekine M., Obayashi M., Nishi T., Shibahara T., Tanaka T., Ishii S.,
RA   Yamamoto J., Saito K., Kawai Y., Isono Y., Nakamura Y., Nagahari K.,
RA   Murakami K., Yasuda T., Iwayanagi T., Wagatsuma M., Shiratori A.,
RA   Sudo H., Hosoiri T., Kaku Y., Kodaira H., Kondo H., Sugawara M.,
RA   Takahashi M., Kanda K., Yokoi T., Furuya T., Kikkawa E., Omura Y.,
RA   Abe K., Kamihara K., Katsuta N., Sato K., Tanikawa M., Yamazaki M.,
RA   Ninomiya K., Ishibashi T., Yamashita H., Murakawa K., Fujimori K.,
RA   Tanai H., Kimata M., Watanabe M., Hiraoka S., Chiba Y., Ishida S.,
RA   Ono Y., Takiguchi S., Watanabe S., Yosida M., Hotuta T., Kusano J.,
RA   Kanehori K., Takahashi-Fujii A., Hara H., Tanase T.-O., Nomura Y.,
RA   Togiya S., Komai F., Hara R., Takeuchi K., Arita M., Imose N.,
RA   Musashino K., Yuuki H., Oshima A., Sasaki N., Aotsuka S.,
RA   Yoshikawa Y., Matsunawa H., Ichihara T., Shiohata N., Sano S.,
RA   Moriya S., Momiyama H., Satoh N., Takami S., Terashima Y., Suzuki O.,
RA   Nakagawa S., Senoh A., Mizoguchi H., Goto Y., Shimizu F., Wakebe H.,
RA   Hishigaki H., Watanabe T., Sugiyama A., Takemoto M., Kawakami B.,
RA   Yamazaki M., Watanabe K., Kumagai A., Itakura S., Fukuzumi Y.,
RA   Fujimori Y., Komiyama M., Tashiro H., Tanigami A., Fujiwara T.,
RA   Ono T., Yamada K., Fujii Y., Ozaki K., Hirao M., Ohmori Y.,
RA   Kawabata A., Hikiji T., Kobatake N., Inagaki H., Ikema Y., Okamoto S.,
RA   Okitani R., Kawakami T., Noguchi S., Itoh T., Shigeta K., Senba T.,
RA   Matsumura K., Nakajima Y., Mizuno T., Morinaga M., Sasaki M.,
RA   Togashi T., Oyama M., Hata H., Watanabe M., Komatsu T.,
RA   Mizushima-Sugano J., Satoh T., Shirai Y., Takahashi Y., Nakagawa K.,
RA   Okumura K., Nagase T., Nomura N., Kikuchi H., Masuho Y., Yamashita R.,
RA   Nakai K., Yada T., Nakamura Y., Ohara O., Isogai T., Sugano S.;
RT   "Complete sequencing and characterization of 21,243 full-length human
RT   cDNAs.";
RL   Nat. Genet. 36:40-45(2004).
RN   [10]
RP   NUCLEOTIDE SEQUENCE [LARGE SCALE MRNA].
RA   Halleck A., Ebert L., Mkoundinya M., Schick M., Eisenstein S.,
RA   Neubert P., Kstrang K., Schatten R., Shen B., Henze S., Mar W.,
RA   Korn B., Zuo D., Hu Y., LaBaer J.;
RT   "Cloning of human full open reading frames in Gateway(TM) system entry
RT   vector (pDONR201).";
RL   Submitted (JUN-2004) to the EMBL/GenBank/DDBJ databases.
RN   [11]
RP   NUCLEOTIDE SEQUENCE [LARGE SCALE GENOMIC DNA].
RX   PubMed=15815621; DOI=10.1038/nature03466;
RA   Hillier L.W., Graves T.A., Fulton R.S., Fulton L.A., Pepin K.H.,
RA   Minx P., Wagner-McPherson C., Layman D., Wylie K., Sekhon M.,
RA   Becker M.C., Fewell G.A., Delehaunty K.D., Miner T.L., Nash W.E.,
RA   Kremitzki C., Oddy L., Du H., Sun H., Bradshaw-Cordum H., Ali J.,
RA   Carter J., Cordes M., Harris A., Isak A., van Brunt A., Nguyen C.,
RA   Du F., Courtney L., Kalicki J., Ozersky P., Abbott S., Armstrong J.,
RA   Belter E.A., Caruso L., Cedroni M., Cotton M., Davidson T., Desai A.,
RA   Elliott G., Erb T., Fronick C., Gaige T., Haakenson W., Haglund K.,
RA   Holmes A., Harkins R., Kim K., Kruchowski S.S., Strong C.M.,
RA   Grewal N., Goyea E., Hou S., Levy A., Martinka S., Mead K.,
RA   McLellan M.D., Meyer R., Randall-Maher J., Tomlinson C.,
RA   Dauphin-Kohlberg S., Kozlowicz-Reilly A., Shah N.,
RA   Swearengen-Shahid S., Snider J., Strong J.T., Thompson J., Yoakum M.,
RA   Leonard S., Pearman C., Trani L., Radionenko M., Waligorski J.E.,
RA   Wang C., Rock S.M., Tin-Wollam A.-M., Maupin R., Latreille P.,
RA   Wendl M.C., Yang S.-P., Pohl C., Wallis J.W., Spieth J., Bieri T.A.,
RA   Berkowicz N., Nelson J.O., Osborne J., Ding L., Meyer R., Sabo A.,
RA   Shotland Y., Sinha P., Wohldmann P.E., Cook L.L., Hickenbotham M.T.,
RA   Eldred J., Williams D., Jones T.A., She X., Ciccarelli F.D.,
RA   Izaurralde E., Taylor J., Schmutz J., Myers R.M., Cox D.R., Huang X.,
RA   McPherson J.D., Mardis E.R., Clifton S.W., Warren W.C.,
RA   Chinwalla A.T., Eddy S.R., Marra M.A., Ovcharenko I., Furey T.S.,
RA   Miller W., Eichler E.E., Bork P., Suyama M., Torrents D.,
RA   Waterston R.H., Wilson R.K.;
RT   "Generation and annotation of the DNA sequences of human chromosomes 2
RT   and 4.";
RL   Nature 434:724-731(2005).
RN   [12]
RP   NUCLEOTIDE SEQUENCE [LARGE SCALE GENOMIC DNA].
RA   Mural R.J., Istrail S., Sutton G.G., Florea L., Halpern A.L.,
RA   Mobarry C.M., Lippert R., Walenz B., Shatkay H., Dew I., Miller J.R.,
RA   Flanigan M.J., Edwards N.J., Bolanos R., Fasulo D., Halldorsson B.V.,
RA   Hannenhalli S., Turner R., Yooseph S., Lu F., Nusskern D.R.,
RA   Shue B.C., Zheng X.H., Zhong F., Delcher A.L., Huson D.H.,
RA   Kravitz S.A., Mouchard L., Reinert K., Remington K.A., Clark A.G.,
RA   Waterman M.S., Eichler E.E., Adams M.D., Hunkapiller M.W., Myers E.W.,
RA   Venter J.C.;
RL   Submitted (SEP-2005) to the EMBL/GenBank/DDBJ databases.
RN   [13]
RP   NUCLEOTIDE SEQUENCE [LARGE SCALE MRNA].
RC   TISSUE=Muscle, Ovary, and Skin;
RX   PubMed=15489334; DOI=10.1101/gr.2596504;
RG   The MGC Project Team;
RT   "The status, quality, and expansion of the NIH full-length cDNA
RT   project: the Mammalian Gene Collection (MGC).";
RL   Genome Res. 14:2121-2127(2004).
RN   [14]
RP   PARTIAL PROTEIN SEQUENCE.
RX   PubMed=2532007;
RA   Rothhut R., Comera C., Cortial S., Haumont P.-Y., Diep Le K.H.,
RA   Cavadore J.-C., Conard J., Russo-Marie F., Lederer F.;
RT   "A 32 kDa lipocortin from human mononuclear cells appears to be
RT   identical with the placental inhibitor of blood coagulation.";
RL   Biochem. J. 263:929-935(1989).
RN   [15]
RP   PROTEIN SEQUENCE OF 7-18; 30-45; 187-201 AND 277-286, AND
RP   IDENTIFICATION BY MASS SPECTROMETRY.
RC   TISSUE=Melanoma;
RA   Quadroni M., Potts A., Barblan J., Bienvenut W.V.;
RL   Submitted (JAN-2005) to UniProtKB.
RN   [16]
RP   PROTEIN SEQUENCE OF 21-31; 93-108; 176-188 AND 304-319, AND
RP   INTERACTION WITH HBV.
RX   PubMed=8249278; DOI=10.1006/viro.1993.1628;
RA   Hertogs K., Leenders W.P., Depla E., De Bruin W.C., Meheus L.,
RA   Raymackers J., Moshage H., Yap S.H.;
RT   "Endonexin II, present on human liver plasma membranes, is a specific
RT   binding protein of small hepatitis B virus (HBV) envelope protein.";
RL   Virology 197:549-557(1993).
RN   [17]
RP   PROTEIN SEQUENCE OF 86-131; 259-297 AND 300-320.
RX   PubMed=2957692; DOI=10.1073/pnas.84.17.6078;
RA   Schlaepfer D.D., Mehlman T., Burgess W.H., Haigler H.T.;
RT   "Structural and functional characterization of endonexin II, a
RT   calcium- and phospholipid-binding protein.";
RL   Proc. Natl. Acad. Sci. U.S.A. 84:6078-6082(1987).
RN   [18]
RP   PROTEIN SEQUENCE OF 85-93.
RC   TISSUE=Placenta;
RX   PubMed=2974032;
RA   Ahn N.G., Teller D.C., Bienkowski M.J., McMullen B.A., Lipkin E.W.,
RA   de Haen C.;
RT   "Sedimentation equilibrium analysis of five lipocortin-related
RT   phospholipase A2 inhibitors from human placenta. Evidence against a
RT   mechanistically relevant association between enzyme and inhibitor.";
RL   J. Biol. Chem. 263:18657-18663(1988).
RN   [19]
RP   PROTEIN SEQUENCE OF 152-161 AND 246-260.
RC   TISSUE=Adipocyte;
RX   PubMed=15242332; DOI=10.1042/BJ20040647;
RA   Aboulaich N., Vainonen J.P., Stralfors P., Vener A.V.;
RT   "Vectorial proteomics reveal targeting, phosphorylation and specific
RT   fragmentation of polymerase I and transcript release factor (PTRF) at
RT   the surface of caveolae in human adipocytes.";
RL   Biochem. J. 383:237-248(2004).
RN   [20]
RP   ACETYLATION [LARGE SCALE ANALYSIS] AT LYS-101, AND IDENTIFICATION BY
RP   MASS SPECTROMETRY [LARGE SCALE ANALYSIS].
RC   TISSUE=Cervix carcinoma;
RX   PubMed=16916647; DOI=10.1016/j.molcel.2006.06.026;
RA   Kim S.C., Sprung R., Chen Y., Xu Y., Ball H., Pei J., Cheng T.,
RA   Kho Y., Xiao H., Xiao L., Grishin N.V., White M., Yang X.-J., Zhao Y.;
RT   "Substrate and functional diversity of lysine acetylation revealed by
RT   a proteomics survey.";
RL   Mol. Cell 23:607-618(2006).
RN   [21]
RP   INVOLVEMENT IN RPRGL3.
RX   PubMed=17339269; DOI=10.1093/hmg/ddm017;
RA   Bogdanova N., Horst J., Chlystun M., Croucher P.J., Nebel A.,
RA   Bohring A., Todorova A., Schreiber S., Gerke V., Krawczak M.,
RA   Markoff A.;
RT   "A common haplotype of the annexin A5 (ANXA5) gene promoter is
RT   associated with recurrent pregnancy loss.";
RL   Hum. Mol. Genet. 16:573-578(2007).
RN   [22]
RP   ACETYLATION [LARGE SCALE ANALYSIS] AT ALA-2, CLEAVAGE OF INITIATOR
RP   METHIONINE [LARGE SCALE ANALYSIS], AND IDENTIFICATION BY MASS
RP   SPECTROMETRY [LARGE SCALE ANALYSIS].
RX   PubMed=19413330; DOI=10.1021/ac9004309;
RA   Gauci S., Helbig A.O., Slijper M., Krijgsveld J., Heck A.J.,
RA   Mohammed S.;
RT   "Lys-N and trypsin cover complementary parts of the phosphoproteome in
RT   a refined SCX-based approach.";
RL   Anal. Chem. 81:4493-4501(2009).
RN   [23]
RP   ACETYLATION [LARGE SCALE ANALYSIS] AT LYS-70; LYS-76; LYS-79 AND
RP   LYS-97, AND IDENTIFICATION BY MASS SPECTROMETRY [LARGE SCALE
RP   ANALYSIS].
RX   PubMed=19608861; DOI=10.1126/science.1175371;
RA   Choudhary C., Kumar C., Gnad F., Nielsen M.L., Rehman M.,
RA   Walther T.C., Olsen J.V., Mann M.;
RT   "Lysine acetylation targets protein complexes and co-regulates major
RT   cellular functions.";
RL   Science 325:834-840(2009).
RN   [24]
RP   IDENTIFICATION BY MASS SPECTROMETRY [LARGE SCALE ANALYSIS].
RX   PubMed=21269460; DOI=10.1186/1752-0509-5-17;
RA   Burkard T.R., Planyavsky M., Kaupe I., Breitwieser F.P.,
RA   Buerckstuemmer T., Bennett K.L., Superti-Furga G., Colinge J.;
RT   "Initial characterization of the human central proteome.";
RL   BMC Syst. Biol. 5:17-17(2011).
RN   [25]
RP   S-NITROSYLATION, AND DOMAIN.
RX   PubMed=25417112; DOI=10.1016/j.cell.2014.09.032;
RA   Jia J., Arif A., Terenzi F., Willard B., Plow E.F., Hazen S.L.,
RA   Fox P.L.;
RT   "Target-selective protein S-nitrosylation by sequence motif
RT   recognition.";
RL   Cell 159:623-634(2014).
RN   [26]
RP   IDENTIFICATION BY MASS SPECTROMETRY [LARGE SCALE ANALYSIS].
RC   TISSUE=Liver;
RX   PubMed=24275569; DOI=10.1016/j.jprot.2013.11.014;
RA   Bian Y., Song C., Cheng K., Dong M., Wang F., Huang J., Sun D.,
RA   Wang L., Ye M., Zou H.;
RT   "An enzyme assisted RP-RPLC approach for in-depth analysis of human
RT   liver phosphoproteome.";
RL   J. Proteomics 96:253-262(2014).
RN   [27]
RP   IDENTIFICATION BY MASS SPECTROMETRY [LARGE SCALE ANALYSIS].
RX   PubMed=25944712; DOI=10.1002/pmic.201400617;
RA   Vaca Jacome A.S., Rabilloud T., Schaeffer-Reiss C., Rompais M.,
RA   Ayoub D., Lane L., Bairoch A., Van Dorsselaer A., Carapito C.;
RT   "N-terminome analysis of the human mitochondrial proteome.";
RL   Proteomics 15:2519-2524(2015).
RN   [28]
RP   X-RAY CRYSTALLOGRAPHY (2.5 ANGSTROMS).
RX   PubMed=2147412;
RA   Huber R., Roemisch J., Paques E.-P.;
RT   "The crystal and molecular structure of human annexin V, an
RT   anticoagulant protein that binds to calcium and membranes.";
RL   EMBO J. 9:3867-3874(1990).
RN   [29]
RP   X-RAY CRYSTALLOGRAPHY (2.0 ANGSTROMS).
RX   PubMed=2148156; DOI=10.1016/0014-5793(90)81428-Q;
RA   Huber R., Schneider M., Mayr I., Roemisch J., Paques E.-P.;
RT   "The calcium binding sites in human annexin V by crystal structure
RT   analysis at 2.0-A resolution. Implications for membrane binding and
RT   calcium channel activity.";
RL   FEBS Lett. 275:15-21(1990).
RN   [30]
RP   X-RAY CRYSTALLOGRAPHY (2.0 ANGSTROMS).
RX   PubMed=1311770; DOI=10.1016/0022-2836(92)90984-R;
RA   Huber R., Berendes R., Burger A., Schneider M., Karshikov A.,
RA   Luecke H., Roemisch J., Paques E.-P.;
RT   "Crystal and molecular structure of human annexin V after refinement.
RT   Implications for structure, membrane binding and ion channel formation
RT   of the annexin family of proteins.";
RL   J. Mol. Biol. 223:683-704(1992).
RN   [31]
RP   X-RAY CRYSTALLOGRAPHY (3.0 ANGSTROMS).
RX   PubMed=9398511; DOI=10.1006/jmbi.1997.1375;
RA   Kaneko N., Ago H., Matsuda R., Inagaki E., Miyano M.;
RT   "Crystal structure of annexin V with its ligand K-201 as a calcium
RT   channel activity inhibitor.";
RL   J. Mol. Biol. 274:16-20(1997).
RN   [32]
RP   X-RAY CRYSTALLOGRAPHY (2.3 ANGSTROMS).
RX   PubMed=9435213; DOI=10.1073/pnas.95.2.455;
RA   Budisa N., Minks C., Medrano F.J., Lutz J., Huber R., Moroder L.;
RT   "Residue-specific bioincorporation of non-natural, biologically active
RT   amino acids into proteins as possible drug carriers: structure and
RT   stability of the per-thiaproline mutant of annexin V.";
RL   Proc. Natl. Acad. Sci. U.S.A. 95:455-459(1998).
CC   -!- FUNCTION: This protein is an anticoagulant protein that acts as an
CC       indirect inhibitor of the thromboplastin-specific complex, which
CC       is involved in the blood coagulation cascade.
CC   -!- SUBUNIT: Monomer. Binds ATRX and EIF5B (By similarity). Interacts
CC       with hepatitis B virus (HBV). {ECO:0000250,
CC       ECO:0000269|PubMed:8249278}.
CC   -!- INTERACTION:
CC       Q9BSI4:TINF2; NbExp=2; IntAct=EBI-296601, EBI-717399;
CC   -!- DOMAIN: The [IL]-x-C-x-x-[DE] motif is a proposed target motif for
CC       cysteine S-nitrosylation mediated by the iNOS-S100A8/A9
CC       transnitrosylase complex. {ECO:0000305|PubMed:25417112}.
CC   -!- DOMAIN: A pair of annexin repeats may form one binding site for
CC       calcium and phospholipid.
CC   -!- PTM: S-nitrosylation is induced by interferon-gamma and
CC       oxidatively-modified low-densitity lipoprotein (LDL(ox)) possibly
CC       implicating the iNOS-S100A8/9 transnitrosylase complex.
CC       {ECO:0000305|PubMed:25417112}.
CC   -!- DISEASE: Pregnancy loss, recurrent, 3 (RPRGL3) [MIM:614391]: A
CC       common complication of pregnancy, resulting in spontaneous
CC       abortion before the fetus has reached viability. The term includes
CC       all miscarriages from the time of conception until 24 weeks of
CC       gestation. Recurrent pregnancy loss is defined as 3 or more
CC       consecutive spontaneous abortions. {ECO:0000269|PubMed:17339269}.
CC       Note=Disease susceptibility is associated with variations
CC       affecting the gene represented in this entry.
CC   -!- SIMILARITY: Belongs to the annexin family. {ECO:0000305}.
CC   -!- SIMILARITY: Contains 4 annexin repeats. {ECO:0000305}.
CC   -!- WEB RESOURCE: Name=R&D Systems' cytokine source book: Annexin V;
CC       URL="http://www.rndsystems.com/molecule_detail.aspx?m=1063";
DR   EMBL; M18366; AAA35570.1; -; mRNA.
DR   EMBL; D00172; BAA00122.1; -; mRNA.
DR   EMBL; X12454; CAA30985.1; -; mRNA.
DR   EMBL; J03745; AAA52386.1; -; mRNA.
DR   EMBL; M21731; AAA36166.1; -; mRNA.
DR   EMBL; M19384; AAB59545.1; -; mRNA.
DR   EMBL; U01691; AAB40047.1; -; Genomic_DNA.
DR   EMBL; U01681; AAB40047.1; JOINED; Genomic_DNA.
DR   EMBL; U01682; AAB40047.1; JOINED; Genomic_DNA.
DR   EMBL; U01683; AAB40047.1; JOINED; Genomic_DNA.
DR   EMBL; U01685; AAB40047.1; JOINED; Genomic_DNA.
DR   EMBL; U01686; AAB40047.1; JOINED; Genomic_DNA.
DR   EMBL; U01687; AAB40047.1; JOINED; Genomic_DNA.
DR   EMBL; U01689; AAB40047.1; JOINED; Genomic_DNA.
DR   EMBL; U01690; AAB40047.1; JOINED; Genomic_DNA.
DR   EMBL; U05770; AAB60648.1; -; Genomic_DNA.
DR   EMBL; U05760; AAB60648.1; JOINED; Genomic_DNA.
DR   EMBL; U05761; AAB60648.1; JOINED; Genomic_DNA.
DR   EMBL; U05762; AAB60648.1; JOINED; Genomic_DNA.
DR   EMBL; U05764; AAB60648.1; JOINED; Genomic_DNA.
DR   EMBL; U05765; AAB60648.1; JOINED; Genomic_DNA.
DR   EMBL; U05766; AAB60648.1; JOINED; Genomic_DNA.
DR   EMBL; U05767; AAB60648.1; JOINED; Genomic_DNA.
DR   EMBL; U05768; AAB60648.1; JOINED; Genomic_DNA.
DR   EMBL; U05769; AAB60648.1; JOINED; Genomic_DNA.
DR   EMBL; AK312644; BAG35528.1; -; mRNA.
DR   EMBL; CR536522; CAG38759.1; -; mRNA.
DR   EMBL; CR541842; CAG46640.1; -; mRNA.
DR   EMBL; AC096730; AAY40954.1; -; Genomic_DNA.
DR   EMBL; CH471056; EAX05257.1; -; Genomic_DNA.
DR   EMBL; CH471056; EAX05258.1; -; Genomic_DNA.
DR   EMBL; BC001429; AAH01429.1; -; mRNA.
DR   EMBL; BC004993; AAH04993.1; -; mRNA.
DR   EMBL; BC012804; AAH12804.1; -; mRNA.
DR   EMBL; BC012822; AAH12822.1; -; mRNA.
DR   EMBL; BC018671; AAH18671.1; -; mRNA.
DR   CCDS; CCDS3720.1; -.
DR   PIR; D29250; AQHUP.
DR   RefSeq; NP_001145.1; NM_001154.3.
DR   UniGene; Hs.480653; -.
DR   PDB; 1ANW; X-ray; 2.40 A; A/B=2-320.
DR   PDB; 1ANX; X-ray; 1.90 A; A/B/C=2-320.
DR   PDB; 1AVH; X-ray; 2.30 A; A/B=1-320.
DR   PDB; 1AVR; X-ray; 2.30 A; A=1-320.
DR   PDB; 1HAK; X-ray; 3.00 A; A/B=1-320.
DR   PDB; 1HVD; X-ray; 2.00 A; A=2-320.
DR   PDB; 1HVE; X-ray; 2.30 A; A=2-320.
DR   PDB; 1HVF; X-ray; 2.00 A; A=2-320.
DR   PDB; 1HVG; X-ray; 3.00 A; A=2-320.
DR   PDB; 1SAV; X-ray; 2.50 A; A=1-320.
DR   PDB; 2XO2; X-ray; 2.80 A; A=1-320.
DR   PDB; 2XO3; X-ray; 2.30 A; A=1-320.
DR   PDBsum; 1ANW; -.
DR   PDBsum; 1ANX; -.
DR   PDBsum; 1AVH; -.
DR   PDBsum; 1AVR; -.
DR   PDBsum; 1HAK; -.
DR   PDBsum; 1HVD; -.
DR   PDBsum; 1HVE; -.
DR   PDBsum; 1HVF; -.
DR   PDBsum; 1HVG; -.
DR   PDBsum; 1SAV; -.
DR   PDBsum; 2XO2; -.
DR   PDBsum; 2XO3; -.
DR   ProteinModelPortal; P08758; -.
DR   SMR; P08758; 3-318.
DR   BioGrid; 106805; 41.
DR   IntAct; P08758; 20.
DR   MINT; MINT-1382250; -.
DR   STRING; 9606.ENSP00000296511; -.
DR   PhosphoSite; P08758; -.
DR   BioMuta; ANXA5; -.
DR   DMDM; 113960; -.
DR   OGP; P08758; -.
DR   REPRODUCTION-2DPAGE; IPI00329801; -.
DR   REPRODUCTION-2DPAGE; P08758; -.
DR   PaxDb; P08758; -.
DR   PeptideAtlas; P08758; -.
DR   PRIDE; P08758; -.
DR   DNASU; 308; -.
DR   Ensembl; ENST00000296511; ENSP00000296511; ENSG00000164111.
DR   GeneID; 308; -.
DR   KEGG; hsa:308; -.
DR   UCSC; uc003idu.4; human.
DR   CTD; 308; -.
DR   GeneCards; ANXA5; -.
DR   HGNC; HGNC:543; ANXA5.
DR   HPA; CAB003677; -.
DR   HPA; HPA035330; -.
DR   MalaCards; ANXA5; -.
DR   MIM; 131230; gene.
DR   MIM; 614391; phenotype.
DR   neXtProt; NX_P08758; -.
DR   PharmGKB; PA24833; -.
DR   eggNOG; KOG0819; Eukaryota.
DR   eggNOG; ENOG410XPUN; LUCA.
DR   HOGENOM; HOG000158803; -.
DR   HOVERGEN; HBG061815; -.
DR   InParanoid; P08758; -.
DR   KO; K16646; -.
DR   OMA; KCIRSVP; -.
DR   OrthoDB; EOG74XS72; -.
DR   PhylomeDB; P08758; -.
DR   TreeFam; TF105452; -.
DR   SignaLink; P08758; -.
DR   ChiTaRS; ANXA5; human.
DR   EvolutionaryTrace; P08758; -.
DR   GeneWiki; Annexin_A5; -.
DR   GenomeRNAi; 308; -.
DR   NextBio; 1243; -.
DR   PRO; PR:P08758; -.
DR   Proteomes; UP000005640; Chromosome 4.
DR   Bgee; P08758; -.
DR   CleanEx; HS_ANXA5; -.
DR   ExpressionAtlas; P08758; baseline and differential.
DR   Genevisible; P08758; HS.
DR   GO; GO:0042995; C:cell projection; IEA:Ensembl.
DR   GO; GO:0005737; C:cytoplasm; TAS:UniProtKB.
DR   GO; GO:0072563; C:endothelial microparticle; IEA:Ensembl.
DR   GO; GO:0009897; C:external side of plasma membrane; IEA:Ensembl.
DR   GO; GO:0070062; C:extracellular exosome; IDA:UniProtKB.
DR   GO; GO:0005925; C:focal adhesion; IDA:UniProtKB.
DR   GO; GO:0014704; C:intercalated disc; IEA:Ensembl.
DR   GO; GO:0005622; C:intracellular; IDA:LIFEdb.
DR   GO; GO:0016020; C:membrane; IDA:UniProtKB.
DR   GO; GO:0005634; C:nucleus; IEA:Ensembl.
DR   GO; GO:0042383; C:sarcolemma; IEA:Ensembl.
DR   GO; GO:0030018; C:Z disc; IEA:Ensembl.
DR   GO; GO:0005509; F:calcium ion binding; IEA:InterPro.
DR   GO; GO:0005544; F:calcium-dependent phospholipid binding; IDA:UniProtKB.
DR   GO; GO:0017046; F:peptide hormone binding; IEA:Ensembl.
DR   GO; GO:0004859; F:phospholipase inhibitor activity; TAS:ProtInc.
DR   GO; GO:0005543; F:phospholipid binding; TAS:ProtInc.
DR   GO; GO:0007596; P:blood coagulation; IEA:UniProtKB-KW.
DR   GO; GO:0098779; P:mitophagy in response to mitochondrial depolarization; IGI:ParkinsonsUK-UCL.
DR   GO; GO:0043066; P:negative regulation of apoptotic process; TAS:UniProtKB.
DR   GO; GO:0030195; P:negative regulation of blood coagulation; IEA:Ensembl.
DR   GO; GO:0043086; P:negative regulation of catalytic activity; TAS:GOC.
DR   GO; GO:0043065; P:positive regulation of apoptotic process; IEA:Ensembl.
DR   GO; GO:0002230; P:positive regulation of defense response to virus by host; IMP:ParkinsonsUK-UCL.
DR   GO; GO:0051260; P:protein homooligomerization; IEA:Ensembl.
DR   GO; GO:1901317; P:regulation of sperm motility; IEA:Ensembl.
DR   GO; GO:0051592; P:response to calcium ion; IEA:Ensembl.
DR   GO; GO:0010033; P:response to organic substance; IEA:Ensembl.
DR   GO; GO:0007165; P:signal transduction; TAS:UniProtKB.
DR   GO; GO:0098792; P:xenophagy; IMP:ParkinsonsUK-UCL.
DR   Gene3D; 1.10.220.10; -; 4.
DR   InterPro; IPR001464; Annexin.
DR   InterPro; IPR018502; Annexin_repeat.
DR   InterPro; IPR018252; Annexin_repeat_CS.
DR   InterPro; IPR015473; Annexins_V.
DR   InterPro; IPR002392; AnnexinV.
DR   PANTHER; PTHR10502:SF26; PTHR10502:SF26; 1.
DR   Pfam; PF00191; Annexin; 4.
DR   PRINTS; PR00196; ANNEXIN.
DR   PRINTS; PR00201; ANNEXINV.
DR   SMART; SM00335; ANX; 4.
DR   PROSITE; PS00223; ANNEXIN; 4.
PE   1: Evidence at protein level;
KW   3D-structure; Acetylation; Annexin; Blood coagulation; Calcium;
KW   Calcium/phospholipid-binding; Complete proteome;
KW   Direct protein sequencing; Hemostasis; Phosphoprotein;
KW   Reference proteome; Repeat; S-nitrosylation.
FT   INIT_MET      1      1       Removed. {ECO:0000244|PubMed:19413330,
FT                                ECO:0000269|PubMed:2963810}.
FT   CHAIN         2    320       Annexin A5.
FT                                /FTId=PRO_0000067487.
FT   REPEAT       24     84       Annexin 1.
FT   REPEAT       96    156       Annexin 2.
FT   REPEAT      180    240       Annexin 3.
FT   REPEAT      255    315       Annexin 4.
FT   MOTIF       314    319       [IL]-x-C-x-x-[DE] motif.
FT                                {ECO:0000305|PubMed:25417112}.
FT   MOD_RES       2      2       N-acetylalanine.
FT                                {ECO:0000244|PubMed:19413330}.
FT   MOD_RES      37     37       Phosphoserine.
FT                                {ECO:0000250|UniProtKB:P48036}.
FT   MOD_RES      70     70       N6-acetyllysine.
FT                                {ECO:0000244|PubMed:19608861}.
FT   MOD_RES      76     76       N6-acetyllysine.
FT                                {ECO:0000244|PubMed:19608861}.
FT   MOD_RES      79     79       N6-acetyllysine.
FT                                {ECO:0000244|PubMed:19608861}.
FT   MOD_RES      97     97       N6-acetyllysine.
FT                                {ECO:0000244|PubMed:19608861}.
FT   MOD_RES     101    101       N6-acetyllysine.
FT                                {ECO:0000244|PubMed:16916647}.
FT   MOD_RES     290    290       N6-succinyllysine.
FT                                {ECO:0000250|UniProtKB:P48036}.
FT   CONFLICT    135    135       S -> L (in Ref. 10; CAG38759).
FT                                {ECO:0000305}.
FT   CONFLICT    279    279       I -> T (in Ref. 13; AAH18671).
FT                                {ECO:0000305}.
FT   HELIX        17     28       {ECO:0000244|PDB:1ANX}.
FT   STRAND       29     32       {ECO:0000244|PDB:1ANX}.
FT   HELIX        35     43       {ECO:0000244|PDB:1ANX}.
FT   HELIX        47     61       {ECO:0000244|PDB:1ANX}.
FT   HELIX        65     72       {ECO:0000244|PDB:1ANX}.
FT   HELIX        75     85       {ECO:0000244|PDB:1ANX}.
FT   HELIX        88    100       {ECO:0000244|PDB:1ANX}.
FT   STRAND      102    104       {ECO:0000244|PDB:1ANX}.
FT   HELIX       107    116       {ECO:0000244|PDB:1ANX}.
FT   HELIX       119    133       {ECO:0000244|PDB:1ANX}.
FT   HELIX       137    144       {ECO:0000244|PDB:1ANX}.
FT   HELIX       147    157       {ECO:0000244|PDB:1ANX}.
FT   HELIX       169    182       {ECO:0000244|PDB:1ANX}.
FT   TURN        183    185       {ECO:0000244|PDB:1ANX}.
FT   STRAND      186    188       {ECO:0000244|PDB:1ANX}.
FT   HELIX       191    200       {ECO:0000244|PDB:1ANX}.
FT   HELIX       203    217       {ECO:0000244|PDB:1ANX}.
FT   HELIX       221    228       {ECO:0000244|PDB:1ANX}.
FT   HELIX       231    245       {ECO:0000244|PDB:1ANX}.
FT   HELIX       247    256       {ECO:0000244|PDB:1ANX}.
FT   HELIX       257    259       {ECO:0000244|PDB:1ANX}.
FT   STRAND      260    263       {ECO:0000244|PDB:1ANX}.
FT   HELIX       266    276       {ECO:0000244|PDB:1ANX}.
FT   TURN        277    280       {ECO:0000244|PDB:1ANX}.
FT   HELIX       281    290       {ECO:0000244|PDB:1ANX}.
FT   STRAND      292    294       {ECO:0000244|PDB:1HVG}.
FT   HELIX       296    303       {ECO:0000244|PDB:1ANX}.
FT   HELIX       306    316       {ECO:0000244|PDB:1ANX}.
SQ   SEQUENCE   320 AA;  35937 MW;  45E14E3964BA4D1A CRC64;
     MAQVLRGTVT DFPGFDERAD AETLRKAMKG LGTDEESILT LLTSRSNAQR QEISAAFKTL
     FGRDLLDDLK SELTGKFEKL IVALMKPSRL YDAYELKHAL KGAGTNEKVL TEIIASRTPE
     ELRAIKQVYE EEYGSSLEDD VVGDTSGYYQ RMLVVLLQAN RDPDAGIDEA QVEQDAQALF
     QAGELKWGTD EEKFITIFGT RSVSHLRKVF DKYMTISGFQ IEETIDRETS GNLEQLLLAV
     VKSIRSIPAY LAETLYYAMK GAGTDDHTLI RVMVSRSEID LFNIRKEFRK NFATSLYSMI
     KGDTSGDYKK ALLLLCGEDD
```

|  |
| --- |
| **Mascot:** http://www.matrixscience.com/ |
