# Supplementary material for: Proteomic profiling identifies the inorganic pyrophosphatase (PPA1) protein as a potential biomarker of metastasis in laryngeal squamous cell carcinoma
Source: Amino Acids. 2016 Mar 7;48:1469–76. doi: 10.1007/s00726-016-2201-8 (PMC4875942; doi:10.1007/s00726-016-2201-8)
Supplement: Supplementary file 5 — Supplementary material 5 (HTML 19 kb) [file 726_2016_2201_MOESM5_ESM.html]

Mascot Search Results: CALR\_HUMAN


# MASCOT Search Results

## Protein View: CALR\_HUMAN

### Calreticulin OS=Homo sapiens GN=CALR PE=1 SV=1

|  |  |
| --- | --- |
| Database: | SwissProt |
| Score: | 172 |
| Expect: | 1.3e-13 |
| Nominal mass (Mr): | 48283 |
| Calculated pI: | 4.29 |
| Taxonomy: | Homo sapiens |

Sequence similarity is available as an NCBI BLAST search of CALR\_HUMAN against nr.

### Search parameters

|  |  |
| --- | --- |
| MS data file: | `peaklist.xml` |
| Enzyme: | Trypsin: cuts C-term side of KR unless next residue is P. |
| Fixed modifications: | Carbamidomethyl (C) |
| Variable modifications: | Oxidation (M) |
|  |  |
| --- | --- |
| Mass values searched: | 54 |
| Mass values matched: | 19 |

### Protein sequence coverage: 51%

Matched peptides shown in ***bold red***.

|  |  |  |  |  |  |
| --- | --- | --- | --- | --- | --- |
| `1` | `MLLSVPLLLG` | `LLGLAVAEPA` | `VYFKEQFLDG` | `DGWTSRWIES` | `KHKSDFGKFV` |
| `51` | `LSSGKFYGDE` | `EKDKGLQTSQ` | `DARFYALSAS` | `FEPFSNKGQT` | `LVVQFTVKHE` |
| `101` | `QNIDCGGGYV` | `KLFPNSLDQT` | `DMHGDSEYNI` | `MFGPDICGPG` | `TKKVHVIFNY` |
| `151` | `KGKNVLINKD` | `IRCKDDEFTH` | `LYTLIVRPDN` | `TYEVKIDNSQ` | `VESGSLEDDW` |
| `201` | `DFLPPKKIKD` | `PDASKPEDWD` | `ERAKIDDPTD` | `SKPEDWDKPE` | `HIPDPDAKKP` |
| `251` | `EDWDEEMDGE` | `WEPPVIQNPE` | `YKGEWKPRQI` | `DNPDYKGTWI` | `HPEIDNPEYS` |
| `301` | `PDPSIYAYDN` | `FGVLGLDLWQ` | `VKSGTIFDNF` | `LITNDEAYAE` | `EFGNETWGVT` |
| `351` | `KAAEKQMKDK` | `QDEEQRLKEE` | `EEDKKRKEEE` | `EAEDKEDDED` | `KDEDEEDEED` |
| `401` | `KEEDEEEDVP` | `GQAKDEL` |  |  |  |

Unformatted sequence string: 417 residues (for pasting into other applications).

Residue Number

Increasing Mass

Decreasing Mass

| Start | – | End | Observed | Mr(expt) | Mr(calc) | Delta | M | Peptide |
| --- | --- | --- | --- | --- | --- | --- | --- | --- |
| 25 | – | 36 | 1410.6464 | 1409.6391 | 1409.6212 | 0.0179 | 0 | K.EQFLDGDGWTSR.W |
| 56 | – | 62 | 887.3286 | 886.3213 | 886.3709 | -0.0496 | 0 | K.FYGDEEK.D |
| 56 | – | 64 | 1130.4652 | 1129.4579 | 1129.4928 | -0.0348 | 1 | K.FYGDEEKDK.G |
| 65 | – | 73 | 975.4559 | 974.4486 | 974.4781 | -0.0295 | 0 | K.GLQTSQDAR.F |
| 74 | – | 87 | 1607.7904 | 1606.7831 | 1606.7667 | 0.0164 | 0 | R.FYALSASFEPFSNK.G |
| 88 | – | 98 | 1219.6713 | 1218.6640 | 1218.6972 | -0.0333 | 0 | K.GQTLVVQFTVK.H |
| 99 | – | 111 | 1476.6793 | 1475.6720 | 1475.6463 | 0.0257 | 0 | K.HEQNIDCGGGYVK.L |
| 143 | – | 151 | 1147.6191 | 1146.6119 | 1146.6550 | -0.0431 | 1 | K.KVHVIFNYK.G |
| 163 | – | 185 | 2856.6123 | 2855.6050 | 2855.3800 | 0.2250 | 1 | R.CKDDEFTHLYTLIVRPDNTYEVK.I |
| 165 | – | 185 | 2568.2266 | 2567.2193 | 2567.2544 | -0.0351 | 0 | K.DDEFTHLYTLIVRPDNTYEVK.I |
| 186 | – | 206 | 2391.1047 | 2390.0975 | 2390.0914 | 0.0061 | 0 | K.IDNSQVESGSLEDDWDFLPPK.K |
| 186 | – | 207 | 2519.1697 | 2518.1624 | 2518.1864 | -0.0240 | 1 | K.IDNSQVESGSLEDDWDFLPPKK.I |
| 208 | – | 222 | 1800.8702 | 1799.8630 | 1799.8326 | 0.0304 | 1 | K.IKDPDASKPEDWDER.A |
| 210 | – | 222 | 1559.7057 | 1558.6984 | 1558.6536 | 0.0448 | 0 | K.DPDASKPEDWDER.A |
| 225 | – | 248 | 2760.3127 | 2759.3055 | 2759.2562 | 0.0492 | 0 | K.IDDPTDSKPEDWDKPEHIPDPDAK.K |
| 279 | – | 286 | 992.4083 | 991.4010 | 991.4611 | -0.0601 | 0 | R.QIDNPDYK.G |
| 323 | – | 351 | 3268.7312 | 3267.7239 | 3267.4884 | 0.2355 | 0 | K.SGTIFDNFLITNDEAYAEEFGNETWGVTK.A |
| 359 | – | 366 | 1047.4501 | 1046.4428 | 1046.4628 | -0.0200 | 1 | K.DKQDEEQR.L |
| 367 | – | 374 | 1019.5157 | 1018.5084 | 1018.4818 | 0.0266 | 1 | R.LKEEEEDK.K |

`No match to: 853.4020, 870.5240, 901.3413, 958.4653, 960.3780, 1061.4692, 1113.4661, 1303.6865, 1327.5280, 1333.7028, 1336.6158, 1366.6003, 1392.6058, 1396.1423, 1410.0952, 1424.6542, 1426.6615, 1442.6436, 1490.7008, 1510.1907, 1547.8887, 1574.7183, 1756.8534, 1814.9020, 1828.9144, 1832.8833, 1909.9927, 2239.1309, 2405.1172, 2774.3113, 2839.4360, 2870.9163, 2974.5032, 2985.6277, 3001.4160`

---

```
AC   P27797; Q6IAT4; Q9UDG2;
DT   01-AUG-1992, integrated into UniProtKB/Swiss-Prot.
DT   01-AUG-1992, sequence version 1.
DT   09-DEC-2015, entry version 192.
DE   RecName: Full=Calreticulin;
DE   AltName: Full=CRP55;
DE   AltName: Full=Calregulin;
DE   AltName: Full=Endoplasmic reticulum resident protein 60;
DE            Short=ERp60;
DE   AltName: Full=HACBP;
DE   AltName: Full=grp60;
DE   Flags: Precursor;
GN   Name=CALR; Synonyms=CRTC;
OS   Homo sapiens (Human).
OC   Eukaryota; Metazoa; Chordata; Craniata; Vertebrata; Euteleostomi;
OC   Mammalia; Eutheria; Euarchontoglires; Primates; Haplorrhini;
OC   Catarrhini; Hominidae; Homo.
OX   NCBI_TaxID=9606;
RN   [1]
RP   NUCLEOTIDE SEQUENCE [MRNA].
RX   PubMed=2332496; DOI=10.1172/JCI114582;
RA   McCauliffe D.P., Lux F.A., Lieu T.S., Sanz I., Hanke J., Newkirk M.M.,
RA   Bachinski L.L., Itoh Y., Siciliano M.J., Reichlin M., Sontheimer R.D.,
RA   Capra J.D.;
RT   "Molecular cloning, expression, and chromosome 19 localization of a
RT   human Ro/SS-A autoantigen.";
RL   J. Clin. Invest. 85:1379-1391(1990).
RN   [2]
RP   NUCLEOTIDE SEQUENCE [MRNA].
RX   PubMed=1919005;
RA   Rokeach L.A., Haselby J.A., Meilof J.F., Smeenk R.J., Unnasch T.R.,
RA   Greene B.M., Hoch S.O.;
RT   "Characterization of the autoantigen calreticulin.";
RL   J. Immunol. 147:3031-3039(1991).
RN   [3]
RP   NUCLEOTIDE SEQUENCE [GENOMIC DNA].
RX   PubMed=1733953;
RA   McCauliffe D.P., Yang Y.S., Wilson J., Sontheimer R.D., Capra J.D.;
RT   "The 5'-flanking region of the human calreticulin gene shares homology
RT   with the human GRP78, GRP94, and protein disulfide isomerase
RT   promoters.";
RL   J. Biol. Chem. 267:2557-2562(1992).
RN   [4]
RP   NUCLEOTIDE SEQUENCE [MRNA].
RA   Liu J., Peng X., Yuan J., Qiang B.;
RL   Submitted (JUL-2001) to the EMBL/GenBank/DDBJ databases.
RN   [5]
RP   NUCLEOTIDE SEQUENCE [LARGE SCALE MRNA].
RX   PubMed=19054851; DOI=10.1038/nmeth.1273;
RA   Goshima N., Kawamura Y., Fukumoto A., Miura A., Honma R., Satoh R.,
RA   Wakamatsu A., Yamamoto J., Kimura K., Nishikawa T., Andoh T., Iida Y.,
RA   Ishikawa K., Ito E., Kagawa N., Kaminaga C., Kanehori K., Kawakami B.,
RA   Kenmochi K., Kimura R., Kobayashi M., Kuroita T., Kuwayama H.,
RA   Maruyama Y., Matsuo K., Minami K., Mitsubori M., Mori M.,
RA   Morishita R., Murase A., Nishikawa A., Nishikawa S., Okamoto T.,
RA   Sakagami N., Sakamoto Y., Sasaki Y., Seki T., Sono S., Sugiyama A.,
RA   Sumiya T., Takayama T., Takayama Y., Takeda H., Togashi T., Yahata K.,
RA   Yamada H., Yanagisawa Y., Endo Y., Imamoto F., Kisu Y., Tanaka S.,
RA   Isogai T., Imai J., Watanabe S., Nomura N.;
RT   "Human protein factory for converting the transcriptome into an in
RT   vitro-expressed proteome.";
RL   Nat. Methods 5:1011-1017(2008).
RN   [6]
RP   NUCLEOTIDE SEQUENCE [LARGE SCALE MRNA].
RA   Kalnine N., Chen X., Rolfs A., Halleck A., Hines L., Eisenstein S.,
RA   Koundinya M., Raphael J., Moreira D., Kelley T., LaBaer J., Lin Y.,
RA   Phelan M., Farmer A.;
RT   "Cloning of human full-length CDSs in BD Creator(TM) system donor
RT   vector.";
RL   Submitted (MAY-2003) to the EMBL/GenBank/DDBJ databases.
RN   [7]
RP   NUCLEOTIDE SEQUENCE [LARGE SCALE MRNA].
RA   Ebert L., Schick M., Neubert P., Schatten R., Henze S., Korn B.;
RT   "Cloning of human full open reading frames in Gateway(TM) system entry
RT   vector (pDONR201).";
RL   Submitted (JUN-2004) to the EMBL/GenBank/DDBJ databases.
RN   [8]
RP   NUCLEOTIDE SEQUENCE [LARGE SCALE GENOMIC DNA].
RX   PubMed=15057824; DOI=10.1038/nature02399;
RA   Grimwood J., Gordon L.A., Olsen A.S., Terry A., Schmutz J.,
RA   Lamerdin J.E., Hellsten U., Goodstein D., Couronne O., Tran-Gyamfi M.,
RA   Aerts A., Altherr M., Ashworth L., Bajorek E., Black S., Branscomb E.,
RA   Caenepeel S., Carrano A.V., Caoile C., Chan Y.M., Christensen M.,
RA   Cleland C.A., Copeland A., Dalin E., Dehal P., Denys M., Detter J.C.,
RA   Escobar J., Flowers D., Fotopulos D., Garcia C., Georgescu A.M.,
RA   Glavina T., Gomez M., Gonzales E., Groza M., Hammon N., Hawkins T.,
RA   Haydu L., Ho I., Huang W., Israni S., Jett J., Kadner K., Kimball H.,
RA   Kobayashi A., Larionov V., Leem S.-H., Lopez F., Lou Y., Lowry S.,
RA   Malfatti S., Martinez D., McCready P.M., Medina C., Morgan J.,
RA   Nelson K., Nolan M., Ovcharenko I., Pitluck S., Pollard M.,
RA   Popkie A.P., Predki P., Quan G., Ramirez L., Rash S., Retterer J.,
RA   Rodriguez A., Rogers S., Salamov A., Salazar A., She X., Smith D.,
RA   Slezak T., Solovyev V., Thayer N., Tice H., Tsai M., Ustaszewska A.,
RA   Vo N., Wagner M., Wheeler J., Wu K., Xie G., Yang J., Dubchak I.,
RA   Furey T.S., DeJong P., Dickson M., Gordon D., Eichler E.E.,
RA   Pennacchio L.A., Richardson P., Stubbs L., Rokhsar D.S., Myers R.M.,
RA   Rubin E.M., Lucas S.M.;
RT   "The DNA sequence and biology of human chromosome 19.";
RL   Nature 428:529-535(2004).
RN   [9]
RP   NUCLEOTIDE SEQUENCE [LARGE SCALE GENOMIC DNA].
RA   Mural R.J., Istrail S., Sutton G.G., Florea L., Halpern A.L.,
RA   Mobarry C.M., Lippert R., Walenz B., Shatkay H., Dew I., Miller J.R.,
RA   Flanigan M.J., Edwards N.J., Bolanos R., Fasulo D., Halldorsson B.V.,
RA   Hannenhalli S., Turner R., Yooseph S., Lu F., Nusskern D.R.,
RA   Shue B.C., Zheng X.H., Zhong F., Delcher A.L., Huson D.H.,
RA   Kravitz S.A., Mouchard L., Reinert K., Remington K.A., Clark A.G.,
RA   Waterman M.S., Eichler E.E., Adams M.D., Hunkapiller M.W., Myers E.W.,
RA   Venter J.C.;
RL   Submitted (JUL-2005) to the EMBL/GenBank/DDBJ databases.
RN   [10]
RP   NUCLEOTIDE SEQUENCE [LARGE SCALE MRNA].
RC   TISSUE=Eye, Pancreas, and Skin;
RX   PubMed=15489334; DOI=10.1101/gr.2596504;
RG   The MGC Project Team;
RT   "The status, quality, and expansion of the NIH full-length cDNA
RT   project: the Mammalian Gene Collection (MGC).";
RL   Genome Res. 14:2121-2127(2004).
RN   [11]
RP   PROTEIN SEQUENCE OF 18-48; 65-92; 96-114; 168-205 AND 257-354.
RC   TISSUE=Placenta;
RX   PubMed=7841019;
RA   Houen G., Koch C.;
RT   "Human placental calreticulin: purification, characterization and
RT   association with other proteins.";
RL   Acta Chem. Scand. 48:905-911(1994).
RN   [12]
RP   PROTEIN SEQUENCE OF 18-41.
RX   PubMed=3260607; DOI=10.1172/JCI113607;
RA   Lieu T.-S., Newkirk M.M., Capra J.D., Sontheimer R.D.;
RT   "Molecular characterization of human Ro/SS-A antigen. Amino terminal
RT   sequence of the protein moiety of human Ro/SS-A antigen and
RT   immunological activity of a corresponding synthetic peptide.";
RL   J. Clin. Invest. 82:96-101(1988).
RN   [13]
RP   PROTEIN SEQUENCE OF 18-36.
RX   PubMed=1911778; DOI=10.1021/bi00105a008;
RA   Rojiani M.V., Finlay B.B., Gray V., Dedhar S.;
RT   "In vitro interaction of a polypeptide homologous to human Ro/SS-A
RT   antigen (calreticulin) with a highly conserved amino acid sequence in
RT   the cytoplasmic domain of integrin alpha subunits.";
RL   Biochemistry 30:9859-9866(1991).
RN   [14]
RP   PROTEIN SEQUENCE OF 18-32.
RX   PubMed=2400400;
RA   Krause K.-H., Simmerman H.K.B., Jones L.R., Campbell K.P.;
RT   "Sequence similarity of calreticulin with a Ca2(+)-binding protein
RT   that co-purifies with an Ins(1,4,5)P3-sensitive Ca2+ store in HL-60
RT   cells.";
RL   Biochem. J. 270:545-548(1990).
RN   [15]
RP   PROTEIN SEQUENCE OF 18-28.
RC   TISSUE=Liver;
RX   PubMed=1286669; DOI=10.1002/elps.11501301201;
RA   Hochstrasser D.F., Frutiger S., Paquet N., Bairoch A., Ravier F.,
RA   Pasquali C., Sanchez J.-C., Tissot J.-D., Bjellqvist B., Vargas R.,
RA   Appel R.D., Hughes G.J.;
RT   "Human liver protein map: a reference database established by
RT   microsequencing and gel comparison.";
RL   Electrophoresis 13:992-1001(1992).
RN   [16]
RP   PROTEIN SEQUENCE OF 25-36; 74-111 AND 208-222, AND IDENTIFICATION BY
RP   MASS SPECTROMETRY.
RC   TISSUE=Brain, Cajal-Retzius cell, and Fetal brain cortex;
RA   Lubec G., Afjehi-Sadat L., Chen W.-Q., Sun Y.;
RL   Submitted (DEC-2008) to UniProtKB.
RN   [17]
RP   PROTEIN SEQUENCE OF 25-34; 56-62; 208-221 AND 273-278.
RC   TISSUE=Keratinocyte;
RX   PubMed=1286667; DOI=10.1002/elps.11501301199;
RA   Rasmussen H.H., van Damme J., Puype M., Gesser B., Celis J.E.,
RA   Vandekerckhove J.;
RT   "Microsequences of 145 proteins recorded in the two-dimensional gel
RT   protein database of normal human epidermal keratinocytes.";
RL   Electrophoresis 13:960-969(1992).
RN   [18]
RP   PROTEIN SEQUENCE OF 18-27, AND SUBCELLULAR LOCATION.
RX   PubMed=8418194; DOI=10.1084/jem.177.1.1;
RA   Dupuis M., Schaerer E., Krause K.-H., Tschopp J.;
RT   "The calcium-binding protein calreticulin is a major constituent of
RT   lytic granules in cytolytic T lymphocytes.";
RL   J. Exp. Med. 177:1-7(1993).
RN   [19]
RP   PROTEIN SEQUENCE OF 18-26.
RC   TISSUE=Colon carcinoma;
RX   PubMed=9150948; DOI=10.1002/elps.1150180344;
RA   Ji H., Reid G.E., Moritz R.L., Eddes J.S., Burgess A.W., Simpson R.J.;
RT   "A two-dimensional gel database of human colon carcinoma proteins.";
RL   Electrophoresis 18:605-613(1997).
RN   [20]
RP   FUNCTION.
RX   PubMed=7876246; DOI=10.1074/jbc.270.9.4741;
RA   Nauseef W.M., McCormick S.J., Clark R.A.;
RT   "Calreticulin functions as a molecular chaperone in the biosynthesis
RT   of myeloperoxidase.";
RL   J. Biol. Chem. 270:4741-4747(1995).
RN   [21]
RP   INTERACTION WITH TRIM21.
RX   PubMed=8666824;
RA   Cheng S.T., Nguyen T.Q., Yang Y.S., Capra J.D., Sontheimer R.D.;
RT   "Calreticulin binds hYRNA and the 52-kDa polypeptide component of the
RT   Ro/SS-A ribonucleoprotein autoantigen.";
RL   J. Immunol. 156:4484-4491(1996).
RN   [22]
RP   SUBCELLULAR LOCATION.
RX   PubMed=10358038; DOI=10.1074/jbc.274.24.16917;
RA   Arosa F.A., de Jesus O., Porto G., Carmo A.M., de Sousa M.;
RT   "Calreticulin is expressed on the cell surface of activated human
RT   peripheral blood T lymphocytes in association with major
RT   histocompatibility complex class I molecules.";
RL   J. Biol. Chem. 274:16917-16922(1999).
RN   [23]
RP   FUNCTION, INTERACTION WITH NR3C1, SUBCELLULAR LOCATION, AND MASS
RP   SPECTROMETRY.
RX   PubMed=11149926; DOI=10.1083/jcb.152.1.127;
RA   Holaska J.M., Black B.E., Love D.C., Hanover J.A., Leszyk J.,
RA   Paschal B.M.;
RT   "Calreticulin is a receptor for nuclear export.";
RL   J. Cell Biol. 152:127-140(2001).
RN   [24]
RP   PARTIAL PROTEIN SEQUENCE, DISULFIDE BOND, AND IDENTIFICATION BY MASS
RP   SPECTROMETRY.
RC   TISSUE=Placenta;
RX   PubMed=11322874; DOI=10.1046/j.1432-1327.2001.02138.x;
RA   Hoejrup P., Roepstorff P., Houen G.;
RT   "Human placental calreticulin characterization of domain structure and
RT   post-translational modifications.";
RL   Eur. J. Biochem. 268:2558-2565(2001).
RN   [25]
RP   GLYCOSYLATION [LARGE SCALE ANALYSIS] AT ASN-344.
RC   TISSUE=Liver;
RX   PubMed=19159218; DOI=10.1021/pr8008012;
RA   Chen R., Jiang X., Sun D., Han G., Wang F., Ye M., Wang L., Zou H.;
RT   "Glycoproteomics analysis of human liver tissue by combination of
RT   multiple enzyme digestion and hydrazide chemistry.";
RL   J. Proteome Res. 8:651-661(2009).
RN   [26]
RP   ACETYLATION [LARGE SCALE ANALYSIS] AT LYS-48; LYS-159 AND LYS-209, AND
RP   IDENTIFICATION BY MASS SPECTROMETRY [LARGE SCALE ANALYSIS].
RX   PubMed=19608861; DOI=10.1126/science.1175371;
RA   Choudhary C., Kumar C., Gnad F., Nielsen M.L., Rehman M.,
RA   Walther T.C., Olsen J.V., Mann M.;
RT   "Lysine acetylation targets protein complexes and co-regulates major
RT   cellular functions.";
RL   Science 325:834-840(2009).
RN   [27]
RP   INTERACTION WITH PPIB.
RX   PubMed=20801878; DOI=10.1074/jbc.M110.160101;
RA   Kozlov G., Bastos-Aristizabal S., Maattanen P., Rosenauer A.,
RA   Zheng F., Killikelly A., Trempe J.F., Thomas D.Y., Gehring K.;
RT   "Structural basis of cyclophilin B binding by the
RT   calnexin/calreticulin P-domain.";
RL   J. Biol. Chem. 285:35551-35557(2010).
RN   [28]
RP   IDENTIFICATION BY MASS SPECTROMETRY [LARGE SCALE ANALYSIS].
RX   PubMed=21269460; DOI=10.1186/1752-0509-5-17;
RA   Burkard T.R., Planyavsky M., Kaupe I., Breitwieser F.P.,
RA   Buerckstuemmer T., Bennett K.L., Superti-Furga G., Colinge J.;
RT   "Initial characterization of the human central proteome.";
RL   BMC Syst. Biol. 5:17-17(2011).
RN   [29]
RP   IDENTIFICATION BY MASS SPECTROMETRY [LARGE SCALE ANALYSIS].
RX   PubMed=22905912; DOI=10.1021/pr300539b;
RA   Rosenow A., Noben J.P., Jocken J., Kallendrusch S.,
RA   Fischer-Posovszky P., Mariman E.C., Renes J.;
RT   "Resveratrol-induced changes of the human adipocyte secretion
RT   profile.";
RL   J. Proteome Res. 11:4733-4743(2012).
RN   [30]
RP   IDENTIFICATION BY MASS SPECTROMETRY [LARGE SCALE ANALYSIS].
RC   TISSUE=Liver;
RX   PubMed=24275569; DOI=10.1016/j.jprot.2013.11.014;
RA   Bian Y., Song C., Cheng K., Dong M., Wang F., Huang J., Sun D.,
RA   Wang L., Ye M., Zou H.;
RT   "An enzyme assisted RP-RPLC approach for in-depth analysis of human
RT   liver phosphoproteome.";
RL   J. Proteomics 96:253-262(2014).
RN   [31]
RP   CLEAVAGE OF SIGNAL PEPTIDE [LARGE SCALE ANALYSIS] AFTER ALA-17, AND
RP   IDENTIFICATION BY MASS SPECTROMETRY [LARGE SCALE ANALYSIS].
RX   PubMed=25944712; DOI=10.1002/pmic.201400617;
RA   Vaca Jacome A.S., Rabilloud T., Schaeffer-Reiss C., Rompais M.,
RA   Ayoub D., Lane L., Bairoch A., Van Dorsselaer A., Carapito C.;
RT   "N-terminome analysis of the human mitochondrial proteome.";
RL   Proteomics 15:2519-2524(2015).
RN   [32]
RP   X-RAY CRYSTALLOGRAPHY (2.3 ANGSTROMS) OF 195-205 IN COMPLEX WITH
RP   GABARAP, AND INTERACTION WITH GABARAP.
RX   PubMed=19154346; DOI=10.1111/j.1742-4658.2008.06857.x;
RA   Thielmann Y., Weiergraber O.H., Mohrluder J., Willbold D.;
RT   "Structural framework of the GABARAP-calreticulin interface --
RT   implications for substrate binding to endoplasmic reticulum
RT   chaperones.";
RL   FEBS J. 276:1140-1152(2009).
RN   [33]
RP   X-RAY CRYSTALLOGRAPHY (1.55 ANGSTROMS) OF 18-368 IN COMPLEX WITH
RP   CALCIUM IONS, AND DISULFIDE BOND.
RX   PubMed=21423620; DOI=10.1371/journal.pone.0017886;
RA   Chouquet A., Paidassi H., Ling W.L., Frachet P., Houen G.,
RA   Arlaud G.J., Gaboriaud C.;
RT   "X-ray structure of the human calreticulin globular domain reveals a
RT   peptide-binding area and suggests a multi-molecular mechanism.";
RL   PLoS ONE 6:E17886-E17886(2011).
CC   -!- FUNCTION: Calcium-binding chaperone that promotes folding,
CC       oligomeric assembly and quality control in the endoplasmic
CC       reticulum (ER) via the calreticulin/calnexin cycle. This lectin
CC       interacts transiently with almost all of the monoglucosylated
CC       glycoproteins that are synthesized in the ER. Interacts with the
CC       DNA-binding domain of NR3C1 and mediates its nuclear export.
CC       Involved in maternal gene expression regulation. May participate
CC       in oocyte maturation via the regulation of calcium homeostasis (By
CC       similarity). {ECO:0000250, ECO:0000269|PubMed:11149926,
CC       ECO:0000269|PubMed:7876246}.
CC   -!- SUBUNIT: Monomer. Component of an EIF2 complex at least composed
CC       of CELF1/CUGBP1, CALR, CALR3, EIF2S1, EIF2S2, HSP90B1 and HSPA5.
CC       Interacts with PDIA3/ERp57 and C9orf9 (By similarity). Interacts
CC       with NR3C1 and TRIM21. Interacts with GABARAP. Interacts with
CC       PPIB. {ECO:0000250|UniProtKB:P14211, ECO:0000250|UniProtKB:P18418,
CC       ECO:0000269|PubMed:11149926, ECO:0000269|PubMed:19154346,
CC       ECO:0000269|PubMed:20801878, ECO:0000269|PubMed:21423620,
CC       ECO:0000269|PubMed:8666824}.
CC   -!- INTERACTION:
CC       P05067:APP; NbExp=2; IntAct=EBI-1049597, EBI-77613;
CC       O95166:GABARAP; NbExp=4; IntAct=EBI-1049597, EBI-712001;
CC       P04792:HSPB1; NbExp=2; IntAct=EBI-1049597, EBI-352682;
CC       Q03518:TAP1; NbExp=2; IntAct=EBI-1049597, EBI-747259;
CC   -!- SUBCELLULAR LOCATION: Endoplasmic reticulum lumen. Cytoplasm,
CC       cytosol. Secreted, extracellular space, extracellular matrix. Cell
CC       surface. Sarcoplasmic reticulum lumen {ECO:0000250}. Note=Also
CC       found in cell surface (T cells), cytosol and extracellular matrix.
CC       Associated with the lytic granules in the cytolytic T-lymphocytes.
CC   -!- DOMAIN: Can be divided into a N-terminal globular domain, a
CC       proline-rich P-domain forming an elongated arm-like structure and
CC       a C-terminal acidic domain. The P-domain binds one molecule of
CC       calcium with high affinity, whereas the acidic C-domain binds
CC       multiple calcium ions with low affinity.
CC   -!- DOMAIN: The interaction with glycans occurs through a binding site
CC       in the globular lectin domain.
CC   -!- DOMAIN: The zinc binding sites are localized to the N-domain.
CC   -!- DOMAIN: Associates with PDIA3 through the tip of the extended arm
CC       formed by the P-domain.
CC   -!- MASS SPECTROMETRY: Mass=46879; Method=MALDI; Range=18-417;
CC       Evidence={ECO:0000269|PubMed:11149926};
CC   -!- SIMILARITY: Belongs to the calreticulin family. {ECO:0000305}.
CC   -!- CAUTION: Was originally thought to be the 52 kDa Ro autoantigen.
CC       {ECO:0000305|PubMed:2332496}.
CC   -!- WEB RESOURCE: Name=Wikipedia; Note=Calreticulin entry;
CC       URL="https://en.wikipedia.org/wiki/Calreticulin";
CC   -!- WEB RESOURCE: Name=Functional Glycomics Gateway - Glycan Binding;
CC       Note=Calreticulin;
CC       URL="http://www.functionalglycomics.org/glycomics/GBPServlet?&operationType=view&cbpId=cbp_hum_other_405";
DR   EMBL; M32294; AAA36582.1; -; mRNA.
DR   EMBL; M84739; AAA51916.1; -; mRNA.
DR   EMBL; AY047586; AAL13126.1; -; mRNA.
DR   EMBL; AB451408; BAG70222.1; -; mRNA.
DR   EMBL; BT007448; AAP36116.1; -; mRNA.
DR   EMBL; CR457070; CAG33351.1; -; mRNA.
DR   EMBL; AD000092; AAB51176.1; -; Genomic_DNA.
DR   EMBL; CH471106; EAW84331.1; -; Genomic_DNA.
DR   EMBL; BC002500; AAH02500.1; -; mRNA.
DR   EMBL; BC007911; AAH07911.1; -; mRNA.
DR   EMBL; BC020493; AAH20493.1; -; mRNA.
DR   CCDS; CCDS12288.1; -.
DR   PIR; A42330; A37047.
DR   RefSeq; NP_004334.1; NM_004343.3.
DR   UniGene; Hs.515162; -.
DR   PDB; 2CLR; X-ray; 2.00 A; C/F=1-10.
DR   PDB; 3DOW; X-ray; 2.30 A; B=195-205.
DR   PDB; 3POS; X-ray; 1.65 A; A/B/C=18-204, A/B/C=302-368.
DR   PDB; 3POW; X-ray; 1.55 A; A=18-204, A=302-368.
DR   PDBsum; 2CLR; -.
DR   PDBsum; 3DOW; -.
DR   PDBsum; 3POS; -.
DR   PDBsum; 3POW; -.
DR   DisProt; DP00333; -.
DR   ProteinModelPortal; P27797; -.
DR   SMR; P27797; 18-367.
DR   BioGrid; 107262; 112.
DR   DIP; DIP-104N; -.
DR   IntAct; P27797; 29.
DR   MINT; MINT-101756; -.
DR   STRING; 9606.ENSP00000320866; -.
DR   DrugBank; DB00025; Antihemophilic Factor.
DR   DrugBank; DB01065; Melatonin.
DR   DrugBank; DB00031; Tenecteplase.
DR   PhosphoSite; P27797; -.
DR   BioMuta; CALR; -.
DR   DMDM; 117501; -.
DR   DOSAC-COBS-2DPAGE; P27797; -.
DR   OGP; P27797; -.
DR   REPRODUCTION-2DPAGE; IPI00020599; -.
DR   SWISS-2DPAGE; P27797; -.
DR   UCD-2DPAGE; P27797; -.
DR   PaxDb; P27797; -.
DR   PRIDE; P27797; -.
DR   DNASU; 811; -.
DR   Ensembl; ENST00000316448; ENSP00000320866; ENSG00000179218.
DR   GeneID; 811; -.
DR   KEGG; hsa:811; -.
DR   UCSC; uc002mvu.2; human.
DR   CTD; 811; -.
DR   GeneCards; CALR; -.
DR   HGNC; HGNC:1455; CALR.
DR   HPA; CAB001513; -.
DR   HPA; CAB019952; -.
DR   HPA; HPA002242; -.
DR   MalaCards; CALR; -.
DR   MIM; 109091; gene.
DR   neXtProt; NX_P27797; -.
DR   Orphanet; 3318; Essential thrombocythemia.
DR   Orphanet; 824; Myelofibrosis with myeloid metaplasia.
DR   PharmGKB; PA26046; -.
DR   eggNOG; KOG0674; Eukaryota.
DR   eggNOG; ENOG410XRR7; LUCA.
DR   HOGENOM; HOG000192435; -.
DR   HOVERGEN; HBG005407; -.
DR   InParanoid; P27797; -.
DR   KO; K08057; -.
DR   OMA; IANPEYV; -.
DR   OrthoDB; EOG77126Z; -.
DR   PhylomeDB; P27797; -.
DR   TreeFam; TF338438; -.
DR   Reactome; R-HSA-1236974; ER-Phagosome pathway.
DR   Reactome; R-HSA-168316; Assembly of Viral Components at the Budding Site.
DR   Reactome; R-HSA-3000480; Scavenging by Class A Receptors.
DR   Reactome; R-HSA-3000484; Scavenging by Class F Receptors.
DR   Reactome; R-HSA-381183; ATF6-alpha activates chaperone genes.
DR   Reactome; R-HSA-901042; Calnexin/calreticulin cycle.
DR   Reactome; R-HSA-983170; Antigen Presentation: Folding, assembly and peptide loading of class I MHC.
DR   ChiTaRS; CALR; human.
DR   EvolutionaryTrace; P27797; -.
DR   GeneWiki; Calreticulin; -.
DR   GenomeRNAi; 811; -.
DR   NextBio; 3292; -.
DR   PRO; PR:P27797; -.
DR   Proteomes; UP000005640; Chromosome 19.
DR   Bgee; P27797; -.
DR   CleanEx; HS_CALR; -.
DR   ExpressionAtlas; P27797; baseline and differential.
DR   Genevisible; P27797; HS.
DR   GO; GO:0001669; C:acrosomal vesicle; IEA:Ensembl.
DR   GO; GO:0009986; C:cell surface; TAS:BHF-UCL.
DR   GO; GO:0005737; C:cytoplasm; IDA:BHF-UCL.
DR   GO; GO:0005829; C:cytosol; IDA:UniProtKB.
DR   GO; GO:0071682; C:endocytic vesicle lumen; TAS:Reactome.
DR   GO; GO:0005783; C:endoplasmic reticulum; TAS:UniProtKB.
DR   GO; GO:0005788; C:endoplasmic reticulum lumen; IDA:UniProtKB.
DR   GO; GO:0009897; C:external side of plasma membrane; IEA:Ensembl.
DR   GO; GO:0070062; C:extracellular exosome; IDA:UniProtKB.
DR   GO; GO:0005576; C:extracellular region; TAS:Reactome.
DR   GO; GO:0005615; C:extracellular space; IDA:UniProtKB.
DR   GO; GO:0005925; C:focal adhesion; IDA:UniProtKB.
DR   GO; GO:0005794; C:Golgi apparatus; IEA:Ensembl.
DR   GO; GO:0071556; C:integral component of lumenal side of endoplasmic reticulum membrane; TAS:Reactome.
DR   GO; GO:0005622; C:intracellular; TAS:UniProtKB.
DR   GO; GO:0016020; C:membrane; IDA:UniProtKB.
DR   GO; GO:0042824; C:MHC class I peptide loading complex; ISS:BHF-UCL.
DR   GO; GO:0005634; C:nucleus; IDA:UniProtKB.
DR   GO; GO:0048471; C:perinuclear region of cytoplasm; IDA:BHF-UCL.
DR   GO; GO:0005844; C:polysome; ISS:BHF-UCL.
DR   GO; GO:0005578; C:proteinaceous extracellular matrix; IEA:UniProtKB-SubCell.
DR   GO; GO:0033018; C:sarcoplasmic reticulum lumen; IEA:UniProtKB-SubCell.
DR   GO; GO:0005790; C:smooth endoplasmic reticulum; IEA:Ensembl.
DR   GO; GO:0050681; F:androgen receptor binding; IDA:BHF-UCL.
DR   GO; GO:0005509; F:calcium ion binding; ISS:UniProtKB.
DR   GO; GO:0030246; F:carbohydrate binding; TAS:BHF-UCL.
DR   GO; GO:0051087; F:chaperone binding; TAS:BHF-UCL.
DR   GO; GO:0001849; F:complement component C1q binding; TAS:BHF-UCL.
DR   GO; GO:0003677; F:DNA binding; NAS:UniProtKB.
DR   GO; GO:0001948; F:glycoprotein binding; IPI:UniProtKB.
DR   GO; GO:0042562; F:hormone binding; IEA:Ensembl.
DR   GO; GO:0005178; F:integrin binding; IPI:BHF-UCL.
DR   GO; GO:0005506; F:iron ion binding; IEA:Ensembl.
DR   GO; GO:0003729; F:mRNA binding; IDA:BHF-UCL.
DR   GO; GO:0042277; F:peptide binding; IEA:Ensembl.
DR   GO; GO:0044822; F:poly(A) RNA binding; IDA:UniProtKB.
DR   GO; GO:0044183; F:protein binding involved in protein folding; TAS:BHF-UCL.
DR   GO; GO:0031625; F:ubiquitin protein ligase binding; IPI:UniProtKB.
DR   GO; GO:0051082; F:unfolded protein binding; TAS:BHF-UCL.
DR   GO; GO:0008270; F:zinc ion binding; TAS:BHF-UCL.
DR   GO; GO:0042590; P:antigen processing and presentation of exogenous peptide antigen via MHC class I; TAS:Reactome.
DR   GO; GO:0002479; P:antigen processing and presentation of exogenous peptide antigen via MHC class I, TAP-dependent; TAS:Reactome.
DR   GO; GO:0002474; P:antigen processing and presentation of peptide antigen via MHC class I; TAS:Reactome.
DR   GO; GO:0036500; P:ATF6-mediated unfolded protein response; TAS:Reactome.
DR   GO; GO:0055007; P:cardiac muscle cell differentiation; IEA:Ensembl.
DR   GO; GO:0007050; P:cell cycle arrest; IGI:BHF-UCL.
DR   GO; GO:0006874; P:cellular calcium ion homeostasis; TAS:UniProtKB.
DR   GO; GO:0044267; P:cellular protein metabolic process; TAS:Reactome.
DR   GO; GO:0071285; P:cellular response to lithium ion; IEA:Ensembl.
DR   GO; GO:0090398; P:cellular senescence; IGI:BHF-UCL.
DR   GO; GO:0061077; P:chaperone-mediated protein folding; IEA:Ensembl.
DR   GO; GO:0030866; P:cortical actin cytoskeleton organization; IEA:Ensembl.
DR   GO; GO:0030968; P:endoplasmic reticulum unfolded protein response; TAS:Reactome.
DR   GO; GO:0042921; P:glucocorticoid receptor signaling pathway; TAS:BHF-UCL.
DR   GO; GO:0033144; P:negative regulation of intracellular steroid hormone receptor signaling pathway; IDA:BHF-UCL.
DR   GO; GO:0045665; P:negative regulation of neuron differentiation; IDA:BHF-UCL.
DR   GO; GO:0048387; P:negative regulation of retinoic acid receptor signaling pathway; IDA:BHF-UCL.
DR   GO; GO:0000122; P:negative regulation of transcription from RNA polymerase II promoter; IDA:BHF-UCL.
DR   GO; GO:0045892; P:negative regulation of transcription, DNA-templated; IDA:BHF-UCL.
DR   GO; GO:0017148; P:negative regulation of translation; ISS:BHF-UCL.
DR   GO; GO:0002502; P:peptide antigen assembly with MHC class I protein complex; ISS:BHF-UCL.
DR   GO; GO:0045787; P:positive regulation of cell cycle; IGI:BHF-UCL.
DR   GO; GO:0008284; P:positive regulation of cell proliferation; IGI:BHF-UCL.
DR   GO; GO:2000510; P:positive regulation of dendritic cell chemotaxis; IMP:UniProtKB.
DR   GO; GO:0045740; P:positive regulation of DNA replication; IGI:BHF-UCL.
DR   GO; GO:0010628; P:positive regulation of gene expression; IEA:Ensembl.
DR   GO; GO:0050766; P:positive regulation of phagocytosis; ISS:BHF-UCL.
DR   GO; GO:1900026; P:positive regulation of substrate adhesion-dependent cell spreading; IMP:UniProtKB.
DR   GO; GO:0043687; P:post-translational protein modification; TAS:Reactome.
DR   GO; GO:0006611; P:protein export from nucleus; IDA:UniProtKB.
DR   GO; GO:0006457; P:protein folding; TAS:BHF-UCL.
DR   GO; GO:0034975; P:protein folding in endoplasmic reticulum; TAS:ParkinsonsUK-UCL.
DR   GO; GO:0034504; P:protein localization to nucleus; IDA:UniProtKB.
DR   GO; GO:0022417; P:protein maturation by protein folding; TAS:BHF-UCL.
DR   GO; GO:0018279; P:protein N-linked glycosylation via asparagine; TAS:Reactome.
DR   GO; GO:0050821; P:protein stabilization; ISS:UniProtKB.
DR   GO; GO:0006898; P:receptor-mediated endocytosis; TAS:Reactome.
DR   GO; GO:0042981; P:regulation of apoptotic process; TAS:UniProtKB.
DR   GO; GO:0040020; P:regulation of meiotic nuclear division; IEA:Ensembl.
DR   GO; GO:0006355; P:regulation of transcription, DNA-templated; TAS:ProtInc.
DR   GO; GO:0042493; P:response to drug; IEA:Ensembl.
DR   GO; GO:0032355; P:response to estradiol; IEA:Ensembl.
DR   GO; GO:0033574; P:response to testosterone; IEA:Ensembl.
DR   GO; GO:0051208; P:sequestering of calcium ion; TAS:BHF-UCL.
DR   GO; GO:0007283; P:spermatogenesis; IEA:Ensembl.
DR   Gene3D; 2.60.120.200; -; 2.
DR   InterPro; IPR001580; Calret/calnex.
DR   InterPro; IPR018124; Calret/calnex_CS.
DR   InterPro; IPR009169; Calreticulin.
DR   InterPro; IPR009033; Calreticulin/calnexin_P_dom.
DR   InterPro; IPR013320; ConA-like_dom.
DR   PANTHER; PTHR11073; PTHR11073; 1.
DR   Pfam; PF00262; Calreticulin; 2.
DR   PIRSF; PIRSF002356; Calreticulin; 1.
DR   PRINTS; PR00626; CALRETICULIN.
DR   SUPFAM; SSF49899; SSF49899; 1.
DR   SUPFAM; SSF63887; SSF63887; 1.
DR   PROSITE; PS00803; CALRETICULIN_1; 1.
DR   PROSITE; PS00804; CALRETICULIN_2; 1.
DR   PROSITE; PS00805; CALRETICULIN_REPEAT; 3.
DR   PROSITE; PS00014; ER_TARGET; 1.
PE   1: Evidence at protein level;
KW   3D-structure; Acetylation; Calcium; Chaperone; Complete proteome;
KW   Cytoplasm; Direct protein sequencing; Disulfide bond;
KW   Endoplasmic reticulum; Extracellular matrix; Glycoprotein; Lectin;
KW   Metal-binding; Reference proteome; Repeat; Sarcoplasmic reticulum;
KW   Secreted; Signal; Zinc.
FT   SIGNAL        1     17       {ECO:0000244|PubMed:25944712,
FT                                ECO:0000269|PubMed:1286669,
FT                                ECO:0000269|PubMed:1911778,
FT                                ECO:0000269|PubMed:2400400,
FT                                ECO:0000269|PubMed:3260607,
FT                                ECO:0000269|PubMed:7841019,
FT                                ECO:0000269|PubMed:8418194,
FT                                ECO:0000269|PubMed:9150948}.
FT   CHAIN        18    417       Calreticulin.
FT                                /FTId=PRO_0000004173.
FT   REPEAT      191    202       1-1.
FT   REPEAT      210    221       1-2.
FT   REPEAT      227    238       1-3.
FT   REPEAT      244    255       1-4.
FT   REPEAT      259    269       2-1.
FT   REPEAT      273    283       2-2.
FT   REPEAT      287    297       2-3.
FT   REGION       18    197       N-domain.
FT   REGION      191    255       4 X approximate repeats.
FT   REGION      198    308       P-domain.
FT   REGION      237    270       Interaction with PPIB. {ECO:0000250}.
FT   REGION      259    297       3 X approximate repeats.
FT   REGION      309    417       C-domain.
FT   MOTIF       414    417       Prevents secretion from ER.
FT   COMPBIAS    351    408       Asp/Glu/Lys-rich.
FT   METAL        26     26       Calcium; via carbonyl oxygen.
FT   METAL        62     62       Calcium; via carbonyl oxygen.
FT   METAL        64     64       Calcium; via carbonyl oxygen.
FT   METAL       328    328       Calcium.
FT   BINDING     109    109       Carbohydrate. {ECO:0000250}.
FT   BINDING     111    111       Carbohydrate. {ECO:0000250}.
FT   BINDING     128    128       Carbohydrate. {ECO:0000250}.
FT   BINDING     135    135       Carbohydrate. {ECO:0000250}.
FT   BINDING     317    317       Carbohydrate. {ECO:0000250}.
FT   MOD_RES      48     48       N6-acetyllysine.
FT                                {ECO:0000244|PubMed:19608861}.
FT   MOD_RES     159    159       N6-acetyllysine.
FT                                {ECO:0000244|PubMed:19608861}.
FT   MOD_RES     209    209       N6-acetyllysine.
FT                                {ECO:0000244|PubMed:19608861}.
FT   CARBOHYD    344    344       N-linked (GlcNAc...).
FT                                {ECO:0000269|PubMed:19159218}.
FT   DISULFID    105    137       {ECO:0000269|PubMed:11322874,
FT                                ECO:0000269|PubMed:21423620}.
FT   STRAND       20     25       {ECO:0000244|PDB:3POW}.
FT   HELIX        30     35       {ECO:0000244|PDB:3POW}.
FT   STRAND       37     39       {ECO:0000244|PDB:3POW}.
FT   STRAND       42     44       {ECO:0000244|PDB:3POW}.
FT   STRAND       49     52       {ECO:0000244|PDB:3POW}.
FT   TURN         60     63       {ECO:0000244|PDB:3POW}.
FT   STRAND       65     68       {ECO:0000244|PDB:3POW}.
FT   STRAND       70     84       {ECO:0000244|PDB:3POW}.
FT   STRAND       91     98       {ECO:0000244|PDB:3POW}.
FT   STRAND      104    107       {ECO:0000244|PDB:3POW}.
FT   STRAND      110    113       {ECO:0000244|PDB:3POW}.
FT   HELIX       119    121       {ECO:0000244|PDB:3POW}.
FT   STRAND      129    138       {ECO:0000244|PDB:3POW}.
FT   STRAND      142    150       {ECO:0000244|PDB:3POW}.
FT   STRAND      153    156       {ECO:0000244|PDB:3POW}.
FT   STRAND      166    176       {ECO:0000244|PDB:3POW}.
FT   STRAND      180    186       {ECO:0000244|PDB:3POW}.
FT   STRAND      189    195       {ECO:0000244|PDB:3POW}.
FT   HELIX       196    199       {ECO:0000244|PDB:3POW}.
FT   STRAND      201    203       {ECO:0000244|PDB:3DOW}.
FT   STRAND      311    322       {ECO:0000244|PDB:3POW}.
FT   STRAND      326    334       {ECO:0000244|PDB:3POW}.
FT   HELIX       336    345       {ECO:0000244|PDB:3POW}.
FT   HELIX       347    366       {ECO:0000244|PDB:3POW}.
SQ   SEQUENCE   417 AA;  48142 MW;  BC37C3C0F1054FB2 CRC64;
     MLLSVPLLLG LLGLAVAEPA VYFKEQFLDG DGWTSRWIES KHKSDFGKFV LSSGKFYGDE
     EKDKGLQTSQ DARFYALSAS FEPFSNKGQT LVVQFTVKHE QNIDCGGGYV KLFPNSLDQT
     DMHGDSEYNI MFGPDICGPG TKKVHVIFNY KGKNVLINKD IRCKDDEFTH LYTLIVRPDN
     TYEVKIDNSQ VESGSLEDDW DFLPPKKIKD PDASKPEDWD ERAKIDDPTD SKPEDWDKPE
     HIPDPDAKKP EDWDEEMDGE WEPPVIQNPE YKGEWKPRQI DNPDYKGTWI HPEIDNPEYS
     PDPSIYAYDN FGVLGLDLWQ VKSGTIFDNF LITNDEAYAE EFGNETWGVT KAAEKQMKDK
     QDEEQRLKEE EEDKKRKEEE EAEDKEDDED KDEDEEDEED KEEDEEEDVP GQAKDEL
```

|  |
| --- |
| **Mascot:** http://www.matrixscience.com/ |
