# Supplementary material for: Proteomic profiling identifies the inorganic pyrophosphatase (PPA1) protein as a potential biomarker of metastasis in laryngeal squamous cell carcinoma
Source: Amino Acids. 2016 Mar 7;48:1469–76. doi: 10.1007/s00726-016-2201-8 (PMC4875942; doi:10.1007/s00726-016-2201-8)
Supplement: Supplementary file 6 — Supplementary material 6 (HTML 10 kb) [file 726_2016_2201_MOESM6_ESM.html]

Mascot Search Results: CH60\_HUMAN


# MASCOT Search Results

## Protein View: CH60\_HUMAN

### 60 kDa heat shock protein, mitochondrial OS=Homo sapiens GN=HSPD1 PE=1 SV=2

|  |  |
| --- | --- |
| Database: | SwissProt |
| Score: | 177 |
| Expect: | 4e-14 |
| Nominal mass (Mr): | 61187 |
| Calculated pI: | 5.70 |
| Taxonomy: | Homo sapiens |

Sequence similarity is available as an NCBI BLAST search of CH60\_HUMAN against nr.

### Search parameters

|  |  |
| --- | --- |
| MS data file: | `peaklist.xml` |
| Enzyme: | Trypsin: cuts C-term side of KR unless next residue is P. |
| Fixed modifications: | Carbamidomethyl (C) |
| Variable modifications: | Oxidation (M) |
|  |  |
| --- | --- |
| Mass values searched: | 45 |
| Mass values matched: | 28 |

### Protein sequence coverage: 49%

Matched peptides shown in ***bold red***.

|  |  |  |  |  |  |
| --- | --- | --- | --- | --- | --- |
| `1` | `MLRLPTVFRQ` | `MRPVSRVLAP` | `HLTRAYAKDV` | `KFGADARALM` | `LQGVDLLADA` |
| `51` | `VAVTMGPKGR` | `TVIIEQSWGS` | `PKVTKDGVTV` | `AKSIDLKDKY` | `KNIGAKLVQD` |
| `101` | `VANNTNEEAG` | `DGTTTATVLA` | `RSIAKEGFEK` | `ISKGANPVEI` | `RRGVMLAVDA` |
| `151` | `VIAELKKQSK` | `PVTTPEEIAQ` | `VATISANGDK` | `EIGNIISDAM` | `KKVGRKGVIT` |
| `201` | `VKDGKTLNDE` | `LEIIEGMKFD` | `RGYISPYFIN` | `TSKGQKCEFQ` | `DAYVLLSEKK` |
| `251` | `ISSIQSIVPA` | `LEIANAHRKP` | `LVIIAEDVDG` | `EALSTLVLNR` | `LKVGLQVVAV` |
| `301` | `KAPGFGDNRK` | `NQLKDMAIAT` | `GGAVFGEEGL` | `TLNLEDVQPH` | `DLGKVGEVIV` |
| `351` | `TKDDAMLLKG` | `KGDKAQIEKR` | `IQEIIEQLDV` | `TTSEYEKEKL` | `NERLAKLSDG` |
| `401` | `VAVLKVGGTS` | `DVEVNEKKDR` | `VTDALNATRA` | `AVEEGIVLGG` | `GCALLRCIPA` |
| `451` | `LDSLTPANED` | `QKIGIEIIKR` | `TLKIPAMTIA` | `KNAGVEGSLI` | `VEKIMQSSSE` |
| `501` | `VGYDAMAGDF` | `VNMVEKGIID` | `PTKVVRTALL` | `DAAGVASLLT` | `TAEVVVTEIP` |
| `551` | `KEEKDPGMGA` | `MGGMGGGMGG` | `GMF` |  |  |

Unformatted sequence string: 573 residues (for pasting into other applications).

Residue Number

Increasing Mass

Decreasing Mass

| Start | – | End | Observed | Mr(expt) | Mr(calc) | Delta | M | Peptide |
| --- | --- | --- | --- | --- | --- | --- | --- | --- |
| 38 | – | 58 | 2113.4873 | 2112.4800 | 2112.1323 | 0.3478 | 0 | R.ALMLQGVDLLADAVAVTMGPK.G |
| 38 | – | 58 | 2129.4893 | 2128.4820 | 2128.1272 | 0.3548 | 0 | R.ALMLQGVDLLADAVAVTMGPK.G + Oxidation (M) |
| 61 | – | 72 | 1344.9128 | 1343.9056 | 1343.7085 | 0.1971 | 0 | R.TVIIEQSWGSPK.V |
| 134 | – | 141 | 855.2727 | 854.2654 | 854.4610 | -0.1956 | 0 | K.GANPVEIR.R |
| 134 | – | 141 | 855.7238 | 854.7165 | 854.4610 | 0.2555 | 0 | K.GANPVEIR.R |
| 142 | – | 156 | 1585.1211 | 1584.1138 | 1583.9069 | 0.2069 | 1 | R.RGVMLAVDAVIAELK.K |
| 143 | – | 156 | 1429.0083 | 1428.0010 | 1427.8058 | 0.1953 | 0 | R.GVMLAVDAVIAELK.K |
| 143 | – | 156 | 1444.9933 | 1443.9860 | 1443.8007 | 0.1853 | 0 | R.GVMLAVDAVIAELK.K + Oxidation (M) |
| 143 | – | 157 | 1557.1228 | 1556.1155 | 1555.9007 | 0.2148 | 1 | R.GVMLAVDAVIAELKK.Q |
| 181 | – | 191 | 1190.7831 | 1189.7758 | 1189.6012 | 0.1746 | 0 | K.EIGNIISDAMK.K |
| 206 | – | 218 | 1504.9569 | 1503.9496 | 1503.7490 | 0.2006 | 0 | K.TLNDELEIIEGMK.F |
| 206 | – | 218 | 1520.9293 | 1519.9220 | 1519.7439 | 0.1781 | 0 | K.TLNDELEIIEGMK.F + Oxidation (M) |
| 237 | – | 249 | 1601.9767 | 1600.9694 | 1600.7443 | 0.2251 | 0 | K.CEFQDAYVLLSEK.K |
| 250 | – | 268 | 2047.4734 | 2046.4661 | 2046.1585 | 0.3076 | 1 | K.KISSIQSIVPALEIANAHR.K |
| 251 | – | 268 | 1919.4113 | 1918.4040 | 1918.0636 | 0.3404 | 0 | K.ISSIQSIVPALEIANAHR.K |
| 269 | – | 290 | 2365.8289 | 2364.8216 | 2364.3264 | 0.4952 | 0 | R.KPLVIIAEDVDGEALSTLVLNR.L |
| 293 | – | 301 | 912.7294 | 911.7221 | 911.5804 | 0.1417 | 0 | K.VGLQVVAVK.A |
| 302 | – | 310 | 961.6763 | 960.6691 | 960.4777 | 0.1913 | 1 | K.APGFGDNRK.N |
| 345 | – | 352 | 844.7040 | 843.6967 | 843.5066 | 0.1901 | 0 | K.VGEVIVTK.D |
| 370 | – | 387 | 2194.4832 | 2193.4759 | 2193.1165 | 0.3594 | 1 | K.RIQEIIEQLDVTTSEYEK.E |
| 371 | – | 387 | 2038.3423 | 2037.3350 | 2037.0153 | 0.3197 | 0 | R.IQEIIEQLDVTTSEYEK.E |
| 421 | – | 429 | 960.7183 | 959.7110 | 959.5036 | 0.2074 | 0 | R.VTDALNATR.A |
| 430 | – | 446 | 1685.2053 | 1684.1980 | 1683.8978 | 0.3003 | 0 | R.AAVEEGIVLGGGCALLR.C |
| 447 | – | 462 | 1772.1251 | 1771.1178 | 1770.8458 | 0.2721 | 0 | R.CIPALDSLTPANEDQK.I |
| 482 | – | 493 | 1215.8148 | 1214.8075 | 1214.6507 | 0.1569 | 0 | K.NAGVEGSLIVEK.I |
| 494 | – | 516 | 2508.5571 | 2507.5499 | 2507.1018 | 0.4480 | 0 | K.IMQSSSEVGYDAMAGDFVNMVEK.G |
| 494 | – | 516 | 2524.5427 | 2523.5354 | 2523.0968 | 0.4387 | 0 | K.IMQSSSEVGYDAMAGDFVNMVEK.G + Oxidation (M) |
| 527 | – | 551 | 2482.8242 | 2481.8169 | 2481.3942 | 0.4228 | 0 | R.TALLDAAGVASLLTTAEVVVTEIPK.E |

`No match to: 869.7404, 870.7339, 1340.8613, 1358.9407, 1376.9199, 1518.9652, 1699.2378, 1755.0948, 1933.4244, 2042.3521, 2052.3704, 2379.7856, 2385.6650, 2560.8022, 2574.7981, 2869.0955, 3098.2422`

---

```
AC   P10809; B2R5M6; B7Z712; Q38L19; Q9UCR6;
DT   01-JUL-1989, integrated into UniProtKB/Swiss-Prot.
DT   01-AUG-1990, sequence version 2.
DT   09-DEC-2015, entry version 195.
DE   RecName: Full=60 kDa heat shock protein, mitochondrial;
DE   AltName: Full=60 kDa chaperonin;
DE   AltName: Full=Chaperonin 60;
DE            Short=CPN60;
DE   AltName: Full=Heat shock protein 60;
DE            Short=HSP-60;
DE            Short=Hsp60;
DE   AltName: Full=HuCHA60;
DE   AltName: Full=Mitochondrial matrix protein P1;
DE   AltName: Full=P60 lymphocyte protein;
DE   Flags: Precursor;
GN   Name=HSPD1; Synonyms=HSP60;
OS   Homo sapiens (Human).
OC   Eukaryota; Metazoa; Chordata; Craniata; Vertebrata; Euteleostomi;
OC   Mammalia; Eutheria; Euarchontoglires; Primates; Haplorrhini;
OC   Catarrhini; Hominidae; Homo.
OX   NCBI_TaxID=9606;
RN   [1]
RP   NUCLEOTIDE SEQUENCE [MRNA] (ISOFORM 1).
RX   PubMed=2568584;
RA   Jindal S., Dudani A.K., Singh B., Harley C.B., Gupta R.S.;
RT   "Primary structure of a human mitochondrial protein homologous to the
RT   bacterial and plant chaperonins and to the 65-kilodalton mycobacterial
RT   antigen.";
RL   Mol. Cell. Biol. 9:2279-2283(1989).
RN   [2]
RP   NUCLEOTIDE SEQUENCE [MRNA] (ISOFORM 1).
RX   PubMed=1980192; DOI=10.1089/dna.1990.9.545;
RA   Venner T.J., Singh B., Gupta R.S.;
RT   "Nucleotide sequences and novel structural features of human and
RT   Chinese hamster hsp60 (chaperonin) gene families.";
RL   DNA Cell Biol. 9:545-552(1990).
RN   [3]
RP   NUCLEOTIDE SEQUENCE [GENOMIC DNA].
RX   PubMed=12483302; DOI=10.1007/s00439-002-0837-9;
RA   Hansen J.J., Bross P., Westergaard M., Nielsen M.N., Eiberg H.,
RA   Boerglum A.D., Mogensen J., Kristiansen K., Bolund L., Gregersen N.;
RT   "Genomic structure of the human mitochondrial chaperonin genes: HSP60
RT   and HSP10 are localised head to head on chromosome 2 separated by a
RT   bidirectional promoter.";
RL   Hum. Genet. 112:71-77(2003).
RN   [4]
RP   NUCLEOTIDE SEQUENCE [GENOMIC DNA].
RA   Tan J., Ong R., Hibberd M.L., Seielstad M.;
RT   "Genetic variation in immune response genes.";
RL   Submitted (SEP-2005) to the EMBL/GenBank/DDBJ databases.
RN   [5]
RP   NUCLEOTIDE SEQUENCE [LARGE SCALE MRNA] (ISOFORMS 1 AND 2).
RC   TISSUE=Adrenal gland, and Spleen;
RX   PubMed=14702039; DOI=10.1038/ng1285;
RA   Ota T., Suzuki Y., Nishikawa T., Otsuki T., Sugiyama T., Irie R.,
RA   Wakamatsu A., Hayashi K., Sato H., Nagai K., Kimura K., Makita H.,
RA   Sekine M., Obayashi M., Nishi T., Shibahara T., Tanaka T., Ishii S.,
RA   Yamamoto J., Saito K., Kawai Y., Isono Y., Nakamura Y., Nagahari K.,
RA   Murakami K., Yasuda T., Iwayanagi T., Wagatsuma M., Shiratori A.,
RA   Sudo H., Hosoiri T., Kaku Y., Kodaira H., Kondo H., Sugawara M.,
RA   Takahashi M., Kanda K., Yokoi T., Furuya T., Kikkawa E., Omura Y.,
RA   Abe K., Kamihara K., Katsuta N., Sato K., Tanikawa M., Yamazaki M.,
RA   Ninomiya K., Ishibashi T., Yamashita H., Murakawa K., Fujimori K.,
RA   Tanai H., Kimata M., Watanabe M., Hiraoka S., Chiba Y., Ishida S.,
RA   Ono Y., Takiguchi S., Watanabe S., Yosida M., Hotuta T., Kusano J.,
RA   Kanehori K., Takahashi-Fujii A., Hara H., Tanase T.-O., Nomura Y.,
RA   Togiya S., Komai F., Hara R., Takeuchi K., Arita M., Imose N.,
RA   Musashino K., Yuuki H., Oshima A., Sasaki N., Aotsuka S.,
RA   Yoshikawa Y., Matsunawa H., Ichihara T., Shiohata N., Sano S.,
RA   Moriya S., Momiyama H., Satoh N., Takami S., Terashima Y., Suzuki O.,
RA   Nakagawa S., Senoh A., Mizoguchi H., Goto Y., Shimizu F., Wakebe H.,
RA   Hishigaki H., Watanabe T., Sugiyama A., Takemoto M., Kawakami B.,
RA   Yamazaki M., Watanabe K., Kumagai A., Itakura S., Fukuzumi Y.,
RA   Fujimori Y., Komiyama M., Tashiro H., Tanigami A., Fujiwara T.,
RA   Ono T., Yamada K., Fujii Y., Ozaki K., Hirao M., Ohmori Y.,
RA   Kawabata A., Hikiji T., Kobatake N., Inagaki H., Ikema Y., Okamoto S.,
RA   Okitani R., Kawakami T., Noguchi S., Itoh T., Shigeta K., Senba T.,
RA   Matsumura K., Nakajima Y., Mizuno T., Morinaga M., Sasaki M.,
RA   Togashi T., Oyama M., Hata H., Watanabe M., Komatsu T.,
RA   Mizushima-Sugano J., Satoh T., Shirai Y., Takahashi Y., Nakagawa K.,
RA   Okumura K., Nagase T., Nomura N., Kikuchi H., Masuho Y., Yamashita R.,
RA   Nakai K., Yada T., Nakamura Y., Ohara O., Isogai T., Sugano S.;
RT   "Complete sequencing and characterization of 21,243 full-length human
RT   cDNAs.";
RL   Nat. Genet. 36:40-45(2004).
RN   [6]
RP   NUCLEOTIDE SEQUENCE [LARGE SCALE GENOMIC DNA].
RX   PubMed=15815621; DOI=10.1038/nature03466;
RA   Hillier L.W., Graves T.A., Fulton R.S., Fulton L.A., Pepin K.H.,
RA   Minx P., Wagner-McPherson C., Layman D., Wylie K., Sekhon M.,
RA   Becker M.C., Fewell G.A., Delehaunty K.D., Miner T.L., Nash W.E.,
RA   Kremitzki C., Oddy L., Du H., Sun H., Bradshaw-Cordum H., Ali J.,
RA   Carter J., Cordes M., Harris A., Isak A., van Brunt A., Nguyen C.,
RA   Du F., Courtney L., Kalicki J., Ozersky P., Abbott S., Armstrong J.,
RA   Belter E.A., Caruso L., Cedroni M., Cotton M., Davidson T., Desai A.,
RA   Elliott G., Erb T., Fronick C., Gaige T., Haakenson W., Haglund K.,
RA   Holmes A., Harkins R., Kim K., Kruchowski S.S., Strong C.M.,
RA   Grewal N., Goyea E., Hou S., Levy A., Martinka S., Mead K.,
RA   McLellan M.D., Meyer R., Randall-Maher J., Tomlinson C.,
RA   Dauphin-Kohlberg S., Kozlowicz-Reilly A., Shah N.,
RA   Swearengen-Shahid S., Snider J., Strong J.T., Thompson J., Yoakum M.,
RA   Leonard S., Pearman C., Trani L., Radionenko M., Waligorski J.E.,
RA   Wang C., Rock S.M., Tin-Wollam A.-M., Maupin R., Latreille P.,
RA   Wendl M.C., Yang S.-P., Pohl C., Wallis J.W., Spieth J., Bieri T.A.,
RA   Berkowicz N., Nelson J.O., Osborne J., Ding L., Meyer R., Sabo A.,
RA   Shotland Y., Sinha P., Wohldmann P.E., Cook L.L., Hickenbotham M.T.,
RA   Eldred J., Williams D., Jones T.A., She X., Ciccarelli F.D.,
RA   Izaurralde E., Taylor J., Schmutz J., Myers R.M., Cox D.R., Huang X.,
RA   McPherson J.D., Mardis E.R., Clifton S.W., Warren W.C.,
RA   Chinwalla A.T., Eddy S.R., Marra M.A., Ovcharenko I., Furey T.S.,
RA   Miller W., Eichler E.E., Bork P., Suyama M., Torrents D.,
RA   Waterston R.H., Wilson R.K.;
RT   "Generation and annotation of the DNA sequences of human chromosomes 2
RT   and 4.";
RL   Nature 434:724-731(2005).
RN   [7]
RP   NUCLEOTIDE SEQUENCE [LARGE SCALE MRNA] (ISOFORM 1).
RC   TISSUE=Lung, Skin, and Uterus;
RX   PubMed=15489334; DOI=10.1101/gr.2596504;
RG   The MGC Project Team;
RT   "The status, quality, and expansion of the NIH full-length cDNA
RT   project: the Mammalian Gene Collection (MGC).";
RL   Genome Res. 14:2121-2127(2004).
RN   [8]
RP   PROTEIN SEQUENCE OF 27-573.
RX   PubMed=2907406;
RA   Waldinger D., Eckerskorn C., Lottspeich F., Cleve H.;
RT   "Amino-acid sequence homology of a polymorphic cellular protein from
RT   human lymphocytes and the chaperonins from Escherichia coli (groEL)
RT   and chloroplasts (Rubisco-binding protein).";
RL   Biol. Chem. Hoppe-Seyler 369:1185-1189(1988).
RN   [9]
RP   PROTEIN SEQUENCE OF 27-55.
RC   TISSUE=Colon carcinoma;
RX   PubMed=2079031; DOI=10.1002/elps.1150111019;
RA   Ward L.D., Hong J., Whitehead R.H., Simpson R.J.;
RT   "Development of a database of amino acid sequences for human colon
RT   carcinoma proteins separated by two-dimensional polyacrylamide gel
RT   electrophoresis.";
RL   Electrophoresis 11:883-891(1990).
RN   [10]
RP   PROTEIN SEQUENCE OF 27-55, AND INTERACTION WITH HTLV-1 P40TAX.
RX   PubMed=1731090;
RA   Nagata K., Ide Y., Takagi T., Ohtani K., Aoshima M., Tozawa H.,
RA   Nakamura M., Sugamura K.;
RT   "Complex formation of human T-cell leukemia virus type I p40tax
RT   transactivator with cellular polypeptides.";
RL   J. Virol. 66:1040-1049(1992).
RN   [11]
RP   PROTEIN SEQUENCE OF 27-50.
RC   TISSUE=Mammary carcinoma;
RX   PubMed=9150946; DOI=10.1002/elps.1150180342;
RA   Rasmussen R.K., Ji H., Eddes J.S., Moritz R.L., Reid G.E.,
RA   Simpson R.J., Dorow D.S.;
RT   "Two-dimensional electrophoretic analysis of human breast carcinoma
RT   proteins: mapping of proteins that bind to the SH3 domain of mixed
RT   lineage kinase MLK2.";
RL   Electrophoresis 18:588-598(1997).
RN   [12]
RP   PROTEIN SEQUENCE OF 27-46.
RC   TISSUE=Heart;
RX   PubMed=7895732; DOI=10.1002/elps.11501501209;
RA   Corbett J.M., Wheeler C.H., Baker C.S., Yacoub M.H., Dunn M.J.;
RT   "The human myocardial two-dimensional gel protein database: update
RT   1994.";
RL   Electrophoresis 15:1459-1465(1994).
RN   [13]
RP   PROTEIN SEQUENCE OF 27-37.
RC   TISSUE=Platelet;
RX   PubMed=12665801; DOI=10.1038/nbt810;
RA   Gevaert K., Goethals M., Martens L., Van Damme J., Staes A.,
RA   Thomas G.R., Vandekerckhove J.;
RT   "Exploring proteomes and analyzing protein processing by mass
RT   spectrometric identification of sorted N-terminal peptides.";
RL   Nat. Biotechnol. 21:566-569(2003).
RN   [14]
RP   PROTEIN SEQUENCE OF 27-35.
RC   TISSUE=Liver;
RX   PubMed=1286669; DOI=10.1002/elps.11501301201;
RA   Hochstrasser D.F., Frutiger S., Paquet N., Bairoch A., Ravier F.,
RA   Pasquali C., Sanchez J.-C., Tissot J.-D., Bjellqvist B., Vargas R.,
RA   Appel R.D., Hughes G.J.;
RT   "Human liver protein map: a reference database established by
RT   microsequencing and gel comparison.";
RL   Electrophoresis 13:992-1001(1992).
RN   [15]
RP   PROTEIN SEQUENCE OF 61-72, AND IDENTIFICATION BY MASS SPECTROMETRY.
RC   TISSUE=B-cell lymphoma;
RA   Bienvenut W.V.;
RL   Submitted (OCT-2004) to UniProtKB.
RN   [16]
RP   PROTEIN SEQUENCE OF 61-72; 206-218; 237-249; 251-290; 430-446 AND
RP   463-469, AND IDENTIFICATION BY MASS SPECTROMETRY.
RC   TISSUE=Brain, Cajal-Retzius cell, and Fetal brain cortex;
RA   Lubec G., Vishwanath V., Chen W.-Q., Sun Y.;
RL   Submitted (DEC-2008) to UniProtKB.
RN   [17]
RP   PROTEIN SEQUENCE OF 97-121; 251-268 AND 430-446.
RC   TISSUE=Adipocyte;
RX   PubMed=15242332; DOI=10.1042/BJ20040647;
RA   Aboulaich N., Vainonen J.P., Stralfors P., Vener A.V.;
RT   "Vectorial proteomics reveal targeting, phosphorylation and specific
RT   fragmentation of polymerase I and transcript release factor (PTRF) at
RT   the surface of caveolae in human adipocytes.";
RL   Biochem. J. 383:237-248(2004).
RN   [18]
RP   PARTIAL PROTEIN SEQUENCE.
RC   TISSUE=Colon carcinoma;
RX   PubMed=9150948; DOI=10.1002/elps.1150180344;
RA   Ji H., Reid G.E., Moritz R.L., Eddes J.S., Burgess A.W., Simpson R.J.;
RT   "A two-dimensional gel database of human colon carcinoma proteins.";
RL   Electrophoresis 18:605-613(1997).
RN   [19]
RP   MITOCHONDRIAL IMPORT.
RX   PubMed=1972619; DOI=10.1016/0006-291X(90)90344-M;
RA   Singh B., Patel H.V., Ridley R.G., Freeman K.B., Gupta R.S.;
RT   "Mitochondrial import of the human chaperonin (HSP60) protein.";
RL   Biochem. Biophys. Res. Commun. 169:391-396(1990).
RN   [20]
RP   INTERACTION WITH HBV PROTEIN X.
RX   PubMed=15120623; DOI=10.1016/j.bbrc.2004.04.046;
RA   Tanaka Y., Kanai F., Kawakami T., Tateishi K., Ijichi H., Kawabe T.,
RA   Arakawa Y., Kawakami T., Nishimura T., Shirakata Y., Koike K.,
RA   Omata M.;
RT   "Interaction of the hepatitis B virus X protein (HBx) with heat shock
RT   protein 60 enhances HBx-mediated apoptosis.";
RL   Biochem. Biophys. Res. Commun. 318:461-469(2004).
RN   [21]
RP   IDENTIFICATION BY MASS SPECTROMETRY [LARGE SCALE ANALYSIS].
RX   PubMed=15592455; DOI=10.1038/nbt1046;
RA   Rush J., Moritz A., Lee K.A., Guo A., Goss V.L., Spek E.J., Zhang H.,
RA   Zha X.-M., Polakiewicz R.D., Comb M.J.;
RT   "Immunoaffinity profiling of tyrosine phosphorylation in cancer
RT   cells.";
RL   Nat. Biotechnol. 23:94-101(2005).
RN   [22]
RP   PHOSPHORYLATION [LARGE SCALE ANALYSIS] AT SER-70, AND IDENTIFICATION
RP   BY MASS SPECTROMETRY [LARGE SCALE ANALYSIS].
RC   TISSUE=Cervix carcinoma;
RX   PubMed=16964243; DOI=10.1038/nbt1240;
RA   Beausoleil S.A., Villen J., Gerber S.A., Rush J., Gygi S.P.;
RT   "A probability-based approach for high-throughput protein
RT   phosphorylation analysis and site localization.";
RL   Nat. Biotechnol. 24:1285-1292(2006).
RN   [23]
RP   PHOSPHORYLATION [LARGE SCALE ANALYSIS] AT SER-70, AND IDENTIFICATION
RP   BY MASS SPECTROMETRY [LARGE SCALE ANALYSIS].
RC   TISSUE=Cervix carcinoma;
RX   PubMed=18669648; DOI=10.1073/pnas.0805139105;
RA   Dephoure N., Zhou C., Villen J., Beausoleil S.A., Bakalarski C.E.,
RA   Elledge S.J., Gygi S.P.;
RT   "A quantitative atlas of mitotic phosphorylation.";
RL   Proc. Natl. Acad. Sci. U.S.A. 105:10762-10767(2008).
RN   [24]
RP   IDENTIFICATION BY MASS SPECTROMETRY [LARGE SCALE ANALYSIS].
RC   TISSUE=Leukemic T-cell;
RX   PubMed=19690332; DOI=10.1126/scisignal.2000007;
RA   Mayya V., Lundgren D.H., Hwang S.-I., Rezaul K., Wu L., Eng J.K.,
RA   Rodionov V., Han D.K.;
RT   "Quantitative phosphoproteomic analysis of T cell receptor signaling
RT   reveals system-wide modulation of protein-protein interactions.";
RL   Sci. Signal. 2:RA46-RA46(2009).
RN   [25]
RP   ACETYLATION [LARGE SCALE ANALYSIS] AT LYS-82; LYS-125; LYS-130;
RP   LYS-202; LYS-218; LYS-269; LYS-352; LYS-359; LYS-396 AND LYS-469, AND
RP   IDENTIFICATION BY MASS SPECTROMETRY [LARGE SCALE ANALYSIS].
RX   PubMed=19608861; DOI=10.1126/science.1175371;
RA   Choudhary C., Kumar C., Gnad F., Nielsen M.L., Rehman M.,
RA   Walther T.C., Olsen J.V., Mann M.;
RT   "Lysine acetylation targets protein complexes and co-regulates major
RT   cellular functions.";
RL   Science 325:834-840(2009).
RN   [26]
RP   PHOSPHORYLATION [LARGE SCALE ANALYSIS] AT SER-70, AND IDENTIFICATION
RP   BY MASS SPECTROMETRY [LARGE SCALE ANALYSIS].
RC   TISSUE=Cervix carcinoma;
RX   PubMed=20068231; DOI=10.1126/scisignal.2000475;
RA   Olsen J.V., Vermeulen M., Santamaria A., Kumar C., Miller M.L.,
RA   Jensen L.J., Gnad F., Cox J., Jensen T.S., Nigg E.A., Brunak S.,
RA   Mann M.;
RT   "Quantitative phosphoproteomics reveals widespread full
RT   phosphorylation site occupancy during mitosis.";
RL   Sci. Signal. 3:RA3-RA3(2010).
RN   [27]
RP   IDENTIFICATION BY MASS SPECTROMETRY [LARGE SCALE ANALYSIS].
RX   PubMed=21269460; DOI=10.1186/1752-0509-5-17;
RA   Burkard T.R., Planyavsky M., Kaupe I., Breitwieser F.P.,
RA   Buerckstuemmer T., Bennett K.L., Superti-Furga G., Colinge J.;
RT   "Initial characterization of the human central proteome.";
RL   BMC Syst. Biol. 5:17-17(2011).
RN   [28]
RP   MALONYLATION AT LYS-133.
RX   PubMed=21908771; DOI=10.1074/mcp.M111.012658;
RA   Peng C., Lu Z., Xie Z., Cheng Z., Chen Y., Tan M., Luo H., Zhang Y.,
RA   He W., Yang K., Zwaans B.M., Tishkoff D., Ho L., Lombard D., He T.C.,
RA   Dai J., Verdin E., Ye Y., Zhao Y.;
RT   "The first identification of lysine malonylation substrates and its
RT   regulatory enzyme.";
RL   Mol. Cell. Proteomics 10:M111.012658.01-M111.012658.12(2011).
RN   [29]
RP   PHOSPHORYLATION [LARGE SCALE ANALYSIS] AT SER-70, AND IDENTIFICATION
RP   BY MASS SPECTROMETRY [LARGE SCALE ANALYSIS].
RX   PubMed=21406692; DOI=10.1126/scisignal.2001570;
RA   Rigbolt K.T., Prokhorova T.A., Akimov V., Henningsen J.,
RA   Johansen P.T., Kratchmarova I., Kassem M., Mann M., Olsen J.V.,
RA   Blagoev B.;
RT   "System-wide temporal characterization of the proteome and
RT   phosphoproteome of human embryonic stem cell differentiation.";
RL   Sci. Signal. 4:RS3-RS3(2011).
RN   [30]
RP   IDENTIFICATION BY MASS SPECTROMETRY [LARGE SCALE ANALYSIS].
RX   PubMed=22905912; DOI=10.1021/pr300539b;
RA   Rosenow A., Noben J.P., Jocken J., Kallendrusch S.,
RA   Fischer-Posovszky P., Mariman E.C., Renes J.;
RT   "Resveratrol-induced changes of the human adipocyte secretion
RT   profile.";
RL   J. Proteome Res. 11:4733-4743(2012).
RN   [31]
RP   INTERACTION WITH ATAD3A.
RX   PubMed=22664726; DOI=10.1016/j.mito.2012.05.005;
RA   Merle N., Feraud O., Gilquin B., Hubstenberger A.,
RA   Kieffer-Jacquinot S., Assard N., Bennaceur-Griscelli A., Honnorat J.,
RA   Baudier J.;
RT   "ATAD3B is a human embryonic stem cell specific mitochondrial protein,
RT   re-expressed in cancer cells, that functions as dominant negative for
RT   the ubiquitous ATAD3A.";
RL   Mitochondrion 12:441-448(2012).
RN   [32]
RP   INTERACTION WITH METTL20 AND METTL21B.
RX   PubMed=23349634; DOI=10.1371/journal.pgen.1003210;
RA   Cloutier P., Lavallee-Adam M., Faubert D., Blanchette M., Coulombe B.;
RT   "A newly uncovered group of distantly related lysine
RT   methyltransferases preferentially interact with molecular chaperones
RT   to regulate their activity.";
RL   PLoS Genet. 9:E1003210-E1003210(2013).
RN   [33]
RP   PHOSPHORYLATION [LARGE SCALE ANALYSIS] AT SER-67 AND TYR-90, AND
RP   IDENTIFICATION BY MASS SPECTROMETRY [LARGE SCALE ANALYSIS].
RC   TISSUE=Liver;
RX   PubMed=24275569; DOI=10.1016/j.jprot.2013.11.014;
RA   Bian Y., Song C., Cheng K., Dong M., Wang F., Huang J., Sun D.,
RA   Wang L., Ye M., Zou H.;
RT   "An enzyme assisted RP-RPLC approach for in-depth analysis of human
RT   liver phosphoproteome.";
RL   J. Proteomics 96:253-262(2014).
RN   [34]
RP   IDENTIFICATION BY MASS SPECTROMETRY [LARGE SCALE ANALYSIS].
RX   PubMed=25944712; DOI=10.1002/pmic.201400617;
RA   Vaca Jacome A.S., Rabilloud T., Schaeffer-Reiss C., Rompais M.,
RA   Ayoub D., Lane L., Bairoch A., Van Dorsselaer A., Carapito C.;
RT   "N-terminome analysis of the human mitochondrial proteome.";
RL   Proteomics 15:2519-2524(2015).
RN   [35]
RP   VARIANT SPG13 ILE-98.
RX   PubMed=11898127; DOI=10.1086/339935;
RA   Hansen J.J., Durr A., Cournu-Rebeix I., Georgopoulos C., Ang D.,
RA   Nielsen M.N., Davoine C.-S., Brice A., Fontaine B., Gregersen N.,
RA   Bross P.;
RT   "Hereditary spastic paraplegia SPG13 is associated with a mutation in
RT   the gene encoding the mitochondrial chaperonin Hsp60.";
RL   Am. J. Hum. Genet. 70:1328-1332(2002).
RN   [36]
RP   VARIANT HLD4 GLY-29, AND CHARACTERIZATION OF VARIANT HLD4 GLY-29.
RX   PubMed=18571143; DOI=10.1016/j.ajhg.2008.05.016;
RA   Magen D., Georgopoulos C., Bross P., Ang D., Segev Y., Goldsher D.,
RA   Nemirovski A., Shahar E., Ravid S., Luder A., Heno B.,
RA   Gershoni-Baruch R., Skorecki K., Mandel H.;
RT   "Mitochondrial Hsp60 chaperonopathy causes an autosomal-recessive
RT   neurodegenerative disorder linked to brain hypomyelination and
RT   leukodystrophy.";
RL   Am. J. Hum. Genet. 83:30-42(2008).
CC   -!- FUNCTION: Implicated in mitochondrial protein import and
CC       macromolecular assembly. May facilitate the correct folding of
CC       imported proteins. May also prevent misfolding and promote the
CC       refolding and proper assembly of unfolded polypeptides generated
CC       under stress conditions in the mitochondrial matrix.
CC   -!- SUBUNIT: Interacts with HRAS (By similarity). Interacts with HBV
CC       protein X and HTLV-1 protein p40tax. Interacts with ATAD3A.
CC       Interacts with METTL20 and METTL21B. {ECO:0000250,
CC       ECO:0000269|PubMed:15120623, ECO:0000269|PubMed:1731090,
CC       ECO:0000269|PubMed:22664726, ECO:0000269|PubMed:23349634}.
CC   -!- INTERACTION:
CC       P38398:BRCA1; NbExp=2; IntAct=EBI-352528, EBI-349905;
CC       Q8NHQ1:CEP70; NbExp=3; IntAct=EBI-352528, EBI-739624;
CC       P49789:FHIT; NbExp=5; IntAct=EBI-352528, EBI-741760;
CC       Q15323:KRT31; NbExp=3; IntAct=EBI-352528, EBI-948001;
CC       Q6A162:KRT40; NbExp=3; IntAct=EBI-352528, EBI-10171697;
CC       P60410:KRTAP10-8; NbExp=3; IntAct=EBI-352528, EBI-10171774;
CC       P26371:KRTAP5-9; NbExp=3; IntAct=EBI-352528, EBI-3958099;
CC       Q9BRK4:LZTS2; NbExp=4; IntAct=EBI-352528, EBI-741037;
CC       Q9Y4C4:MFHAS1; NbExp=3; IntAct=EBI-352528, EBI-2864441;
CC       Q7Z3S9:NOTCH2NL; NbExp=3; IntAct=EBI-352528, EBI-945833;
CC       O76081:RGS20; NbExp=3; IntAct=EBI-352528, EBI-1052678;
CC       O76081-6:RGS20; NbExp=3; IntAct=EBI-352528, EBI-10178530;
CC       Q8N6K7:SAMD3; NbExp=3; IntAct=EBI-352528, EBI-748741;
CC       Q9JJY3:Smpd3 (xeno); NbExp=3; IntAct=EBI-352528, EBI-9817007;
CC       Q7Z6C6:TMCC2; NbExp=3; IntAct=EBI-352528, EBI-10177480;
CC   -!- SUBCELLULAR LOCATION: Mitochondrion matrix.
CC   -!- ALTERNATIVE PRODUCTS:
CC       Event=Alternative splicing; Named isoforms=2;
CC       Name=1;
CC         IsoId=P10809-1; Sequence=Displayed;
CC       Name=2;
CC         IsoId=P10809-2; Sequence=VSP_056144, VSP_056145;
CC         Note=No experimental confirmation available.;
CC   -!- DISEASE: Spastic paraplegia 13, autosomal dominant (SPG13)
CC       [MIM:605280]: A form of spastic paraplegia, a neurodegenerative
CC       disorder characterized by a slow, gradual, progressive weakness
CC       and spasticity of the lower limbs. Rate of progression and the
CC       severity of symptoms are quite variable. Initial symptoms may
CC       include difficulty with balance, weakness and stiffness in the
CC       legs, muscle spasms, and dragging the toes when walking. In some
CC       forms of the disorder, bladder symptoms (such as incontinence) may
CC       appear, or the weakness and stiffness may spread to other parts of
CC       the body. {ECO:0000269|PubMed:11898127}. Note=The disease is
CC       caused by mutations affecting the gene represented in this entry.
CC   -!- DISEASE: Leukodystrophy, hypomyelinating, 4 (HLD4) [MIM:612233]: A
CC       severe autosomal recessive hypomyelinating leukodystrophy.
CC       Clinically characterized by infantile-onset rotary nystagmus,
CC       progressive spastic paraplegia, neurologic regression, motor
CC       impairment, profound mental retardation. Death usually occurs
CC       within the first two decades of life.
CC       {ECO:0000269|PubMed:18571143}. Note=The disease is caused by
CC       mutations affecting the gene represented in this entry.
CC   -!- SIMILARITY: Belongs to the chaperonin (HSP60) family.
CC       {ECO:0000305}.
CC   -!- WEB RESOURCE: Name=Atlas of Genetics and Cytogenetics in Oncology
CC       and Haematology;
CC       URL="http://atlasgeneticsoncology.org/Genes/HSPD1ID40888ch2q33.html";
DR   EMBL; M22382; AAA60127.1; -; mRNA.
DR   EMBL; M34664; AAA36022.1; -; mRNA.
DR   EMBL; AJ250915; CAB75426.1; -; Genomic_DNA.
DR   EMBL; DQ217936; ABB01006.1; -; Genomic_DNA.
DR   EMBL; AK301276; BAH13448.1; -; mRNA.
DR   EMBL; AK312240; BAG35173.1; -; mRNA.
DR   EMBL; AC010746; -; NOT_ANNOTATED_CDS; Genomic_DNA.
DR   EMBL; AC020550; -; NOT_ANNOTATED_CDS; Genomic_DNA.
DR   EMBL; AC114809; -; NOT_ANNOTATED_CDS; Genomic_DNA.
DR   EMBL; BC002676; AAH02676.1; -; mRNA.
DR   EMBL; BC003030; AAH03030.1; -; mRNA.
DR   EMBL; BC067082; AAH67082.1; -; mRNA.
DR   EMBL; BC073746; AAH73746.1; -; mRNA.
DR   CCDS; CCDS33357.1; -. [P10809-1]
DR   PIR; A32800; A32800.
DR   RefSeq; NP_002147.2; NM_002156.4. [P10809-1]
DR   RefSeq; NP_955472.1; NM_199440.1. [P10809-1]
DR   RefSeq; XP_005246575.1; XM_005246518.2. [P10809-1]
DR   UniGene; Hs.595053; -.
DR   UniGene; Hs.727543; -.
DR   PDB; 4PJ1; X-ray; 3.15 A; A/B/C/D/E/F/G/H/I/J/K/L/M/N=27-556.
DR   PDBsum; 4PJ1; -.
DR   ProteinModelPortal; P10809; -.
DR   SMR; P10809; 27-550.
DR   BioGrid; 109561; 240.
DR   DIP; DIP-58N; -.
DR   IntAct; P10809; 105.
DR   MINT; MINT-1162735; -.
DR   STRING; 9606.ENSP00000340019; -.
DR   ChEMBL; CHEMBL4721; -.
DR   PhosphoSite; P10809; -.
DR   BioMuta; HSPD1; -.
DR   DMDM; 129379; -.
DR   DOSAC-COBS-2DPAGE; P10809; -.
DR   OGP; P10809; -.
DR   REPRODUCTION-2DPAGE; IPI00784154; -.
DR   REPRODUCTION-2DPAGE; P10809; -.
DR   SWISS-2DPAGE; P10809; -.
DR   UCD-2DPAGE; P10809; -.
DR   MaxQB; P10809; -.
DR   PaxDb; P10809; -.
DR   PRIDE; P10809; -.
DR   DNASU; 3329; -.
DR   Ensembl; ENST00000345042; ENSP00000340019; ENSG00000144381. [P10809-1]
DR   Ensembl; ENST00000388968; ENSP00000373620; ENSG00000144381. [P10809-1]
DR   GeneID; 3329; -.
DR   KEGG; hsa:3329; -.
DR   UCSC; uc002uui.3; human. [P10809-1]
DR   CTD; 3329; -.
DR   GeneCards; HSPD1; -.
DR   HGNC; HGNC:5261; HSPD1.
DR   HPA; CAB002775; -.
DR   HPA; HPA001523; -.
DR   HPA; HPA050025; -.
DR   MalaCards; HSPD1; -.
DR   MIM; 118190; gene.
DR   MIM; 605280; phenotype.
DR   MIM; 612233; phenotype.
DR   neXtProt; NX_P10809; -.
DR   Orphanet; 100994; Autosomal dominant spastic paraplegia type 13.
DR   Orphanet; 280288; Pelizaeus-Merzbacher-like disease due to HSPD1 mutation.
DR   PharmGKB; PA29527; -.
DR   eggNOG; KOG0356; Eukaryota.
DR   eggNOG; COG0459; LUCA.
DR   GeneTree; ENSGT00390000005727; -.
DR   HOGENOM; HOG000076290; -.
DR   HOVERGEN; HBG001982; -.
DR   InParanoid; P10809; -.
DR   KO; K04077; -.
DR   OMA; RSRIVKG; -.
DR   OrthoDB; EOG7HTHGJ; -.
DR   PhylomeDB; P10809; -.
DR   TreeFam; TF300475; -.
DR   Reactome; R-HSA-1268020; Mitochondrial protein import.
DR   ChiTaRS; HSPD1; human.
DR   GeneWiki; GroEL; -.
DR   GenomeRNAi; 3329; -.
DR   NextBio; 13188; -.
DR   PRO; PR:P10809; -.
DR   Proteomes; UP000005640; Chromosome 2.
DR   Bgee; P10809; -.
DR   CleanEx; HS_HSPD1; -.
DR   ExpressionAtlas; P10809; baseline and differential.
DR   Genevisible; P10809; HS.
DR   GO; GO:0009986; C:cell surface; IDA:UniProtKB.
DR   GO; GO:0005905; C:coated pit; IDA:BHF-UCL.
DR   GO; GO:0030135; C:coated vesicle; IDA:BHF-UCL.
DR   GO; GO:0005737; C:cytoplasm; IDA:UniProtKB.
DR   GO; GO:0005829; C:cytosol; IDA:UniProtKB.
DR   GO; GO:0005769; C:early endosome; IDA:BHF-UCL.
DR   GO; GO:0070062; C:extracellular exosome; IDA:UniProtKB.
DR   GO; GO:0005615; C:extracellular space; IDA:BHF-UCL.
DR   GO; GO:0005794; C:Golgi apparatus; IEA:Ensembl.
DR   GO; GO:0046696; C:lipopolysaccharide receptor complex; IDA:BHF-UCL.
DR   GO; GO:0016020; C:membrane; IDA:UniProtKB.
DR   GO; GO:0045121; C:membrane raft; IEA:Ensembl.
DR   GO; GO:0030061; C:mitochondrial crista; IEA:Ensembl.
DR   GO; GO:0005743; C:mitochondrial inner membrane; ISS:BHF-UCL.
DR   GO; GO:0005759; C:mitochondrial matrix; TAS:BHF-UCL.
DR   GO; GO:0005739; C:mitochondrion; IDA:UniProtKB.
DR   GO; GO:0043209; C:myelin sheath; IEA:Ensembl.
DR   GO; GO:0005886; C:plasma membrane; IEA:Ensembl.
DR   GO; GO:0043234; C:protein complex; IDA:UniProtKB.
DR   GO; GO:0005791; C:rough endoplasmic reticulum; IEA:Ensembl.
DR   GO; GO:0030141; C:secretory granule; ISS:BHF-UCL.
DR   GO; GO:0042588; C:zymogen granule; IEA:Ensembl.
DR   GO; GO:0005524; F:ATP binding; IEA:UniProtKB-KW.
DR   GO; GO:0016887; F:ATPase activity; ISS:BHF-UCL.
DR   GO; GO:0051087; F:chaperone binding; IPI:UniProtKB.
DR   GO; GO:0003688; F:DNA replication origin binding; ISS:BHF-UCL.
DR   GO; GO:0003725; F:double-stranded RNA binding; IDA:MGI.
DR   GO; GO:0001530; F:lipopolysaccharide binding; IDA:BHF-UCL.
DR   GO; GO:0002039; F:p53 binding; IPI:UniProtKB.
DR   GO; GO:0044822; F:poly(A) RNA binding; IDA:UniProtKB.
DR   GO; GO:0003697; F:single-stranded DNA binding; ISS:BHF-UCL.
DR   GO; GO:0031625; F:ubiquitin protein ligase binding; IPI:ParkinsonsUK-UCL.
DR   GO; GO:0051082; F:unfolded protein binding; IC:UniProtKB.
DR   GO; GO:0006458; P:'de novo' protein folding; ISS:BHF-UCL.
DR   GO; GO:0006919; P:activation of cysteine-type endopeptidase activity involved in apoptotic process; IDA:BHF-UCL.
DR   GO; GO:0042113; P:B cell activation; IDA:BHF-UCL.
DR   GO; GO:0002368; P:B cell cytokine production; IDA:BHF-UCL.
DR   GO; GO:0042100; P:B cell proliferation; IDA:BHF-UCL.
DR   GO; GO:0051085; P:chaperone mediated protein folding requiring cofactor; IEA:Ensembl.
DR   GO; GO:0051131; P:chaperone-mediated protein complex assembly; ISS:BHF-UCL.
DR   GO; GO:0002236; P:detection of misfolded protein; IEA:Ensembl.
DR   GO; GO:0048291; P:isotype switching to IgG isotypes; IDA:BHF-UCL.
DR   GO; GO:0002755; P:MyD88-dependent toll-like receptor signaling pathway; IDA:BHF-UCL.
DR   GO; GO:0043066; P:negative regulation of apoptotic process; IMP:UniProtKB.
DR   GO; GO:0043524; P:negative regulation of neuron apoptotic process; IEA:Ensembl.
DR   GO; GO:0043065; P:positive regulation of apoptotic process; IMP:BHF-UCL.
DR   GO; GO:0050729; P:positive regulation of inflammatory response; IEA:Ensembl.
DR   GO; GO:0032727; P:positive regulation of interferon-alpha production; IDA:BHF-UCL.
DR   GO; GO:0032729; P:positive regulation of interferon-gamma production; IDA:BHF-UCL.
DR   GO; GO:0032733; P:positive regulation of interleukin-10 production; IDA:BHF-UCL.
DR   GO; GO:0032735; P:positive regulation of interleukin-12 production; IDA:BHF-UCL.
DR   GO; GO:0032755; P:positive regulation of interleukin-6 production; IDA:BHF-UCL.
DR   GO; GO:0043032; P:positive regulation of macrophage activation; IDA:BHF-UCL.
DR   GO; GO:0050870; P:positive regulation of T cell activation; IDA:BHF-UCL.
DR   GO; GO:0002842; P:positive regulation of T cell mediated immune response to tumor cell; IDA:BHF-UCL.
DR   GO; GO:0051604; P:protein maturation; ISS:BHF-UCL.
DR   GO; GO:0042026; P:protein refolding; IDA:UniProtKB.
DR   GO; GO:0050821; P:protein stabilization; IMP:UniProtKB.
DR   GO; GO:0033198; P:response to ATP; IEA:Ensembl.
DR   GO; GO:0042220; P:response to cocaine; IEA:Ensembl.
DR   GO; GO:0009409; P:response to cold; ISS:AgBase.
DR   GO; GO:0042493; P:response to drug; IEA:Ensembl.
DR   GO; GO:0043627; P:response to estrogen; IEA:Ensembl.
DR   GO; GO:0009408; P:response to heat; IEA:Ensembl.
DR   GO; GO:0042542; P:response to hydrogen peroxide; IEA:Ensembl.
DR   GO; GO:0001666; P:response to hypoxia; IEA:Ensembl.
DR   GO; GO:0032496; P:response to lipopolysaccharide; IEA:Ensembl.
DR   GO; GO:0006986; P:response to unfolded protein; IDA:BHF-UCL.
DR   GO; GO:0042110; P:T cell activation; IDA:MGI.
DR   GO; GO:0016032; P:viral process; IEA:UniProtKB-KW.
DR   Gene3D; 1.10.560.10; -; 2.
DR   Gene3D; 3.50.7.10; -; 1.
DR   HAMAP; MF_00600; CH60; 1.
DR   InterPro; IPR018370; Chaperonin_Cpn60_CS.
DR   InterPro; IPR001844; Chaprnin_Cpn60.
DR   InterPro; IPR002423; Cpn60/TCP-1.
DR   InterPro; IPR027409; GroEL-like_apical_dom.
DR   InterPro; IPR027413; GROEL-like_equatorial.
DR   Pfam; PF00118; Cpn60_TCP1; 1.
DR   PRINTS; PR00298; CHAPERONIN60.
DR   SUPFAM; SSF52029; SSF52029; 1.
DR   TIGRFAMs; TIGR02348; GroEL; 1.
DR   PROSITE; PS00296; CHAPERONINS_CPN60; 1.
PE   1: Evidence at protein level;
KW   3D-structure; Acetylation; Alternative splicing; ATP-binding;
KW   Chaperone; Complete proteome; Direct protein sequencing;
KW   Disease mutation; Hereditary spastic paraplegia;
KW   Host-virus interaction; Leukodystrophy; Mitochondrion;
KW   Neurodegeneration; Nucleotide-binding; Phosphoprotein;
KW   Reference proteome; Transit peptide.
FT   TRANSIT       1     26       Mitochondrion.
FT                                {ECO:0000269|PubMed:12665801,
FT                                ECO:0000269|PubMed:1286669,
FT                                ECO:0000269|PubMed:1731090,
FT                                ECO:0000269|PubMed:2079031,
FT                                ECO:0000269|PubMed:2907406,
FT                                ECO:0000269|PubMed:7895732,
FT                                ECO:0000269|PubMed:9150946}.
FT   CHAIN        27    573       60 kDa heat shock protein, mitochondrial.
FT                                /FTId=PRO_0000005026.
FT   MOD_RES      31     31       N6-succinyllysine.
FT                                {ECO:0000250|UniProtKB:P63038}.
FT   MOD_RES      67     67       Phosphoserine.
FT                                {ECO:0000244|PubMed:24275569}.
FT   MOD_RES      70     70       Phosphoserine.
FT                                {ECO:0000244|PubMed:16964243,
FT                                ECO:0000244|PubMed:18669648,
FT                                ECO:0000244|PubMed:20068231,
FT                                ECO:0000244|PubMed:21406692}.
FT   MOD_RES      75     75       N6-acetyllysine.
FT                                {ECO:0000250|UniProtKB:P63038}.
FT   MOD_RES      82     82       N6-acetyllysine; alternate.
FT                                {ECO:0000244|PubMed:19608861}.
FT   MOD_RES      82     82       N6-succinyllysine; alternate.
FT                                {ECO:0000250|UniProtKB:P63038}.
FT   MOD_RES      87     87       N6-acetyllysine.
FT                                {ECO:0000250|UniProtKB:P63038}.
FT   MOD_RES      90     90       Phosphotyrosine.
FT                                {ECO:0000244|PubMed:24275569}.
FT   MOD_RES      91     91       N6-acetyllysine.
FT                                {ECO:0000250|UniProtKB:P63038}.
FT   MOD_RES     125    125       N6-acetyllysine; alternate.
FT                                {ECO:0000244|PubMed:19608861}.
FT   MOD_RES     125    125       N6-succinyllysine; alternate.
FT                                {ECO:0000250|UniProtKB:P63038}.
FT   MOD_RES     130    130       N6-acetyllysine.
FT                                {ECO:0000244|PubMed:19608861}.
FT   MOD_RES     133    133       N6-acetyllysine; alternate.
FT                                {ECO:0000250|UniProtKB:P63038}.
FT   MOD_RES     133    133       N6-malonyllysine; alternate.
FT                                {ECO:0000269|PubMed:21908771}.
FT   MOD_RES     133    133       N6-succinyllysine; alternate.
FT                                {ECO:0000250|UniProtKB:P63038}.
FT   MOD_RES     156    156       N6-acetyllysine.
FT                                {ECO:0000250|UniProtKB:P63038}.
FT   MOD_RES     191    191       N6-acetyllysine; alternate.
FT                                {ECO:0000250|UniProtKB:P63038}.
FT   MOD_RES     191    191       N6-succinyllysine; alternate.
FT                                {ECO:0000250|UniProtKB:P63038}.
FT   MOD_RES     202    202       N6-acetyllysine; alternate.
FT                                {ECO:0000244|PubMed:19608861}.
FT   MOD_RES     202    202       N6-succinyllysine; alternate.
FT                                {ECO:0000250|UniProtKB:P63038}.
FT   MOD_RES     205    205       N6-acetyllysine; alternate.
FT                                {ECO:0000250|UniProtKB:P63038}.
FT   MOD_RES     205    205       N6-succinyllysine; alternate.
FT                                {ECO:0000250|UniProtKB:P63038}.
FT   MOD_RES     218    218       N6-acetyllysine; alternate.
FT                                {ECO:0000244|PubMed:19608861}.
FT   MOD_RES     218    218       N6-succinyllysine; alternate.
FT                                {ECO:0000250|UniProtKB:P63038}.
FT   MOD_RES     236    236       N6-acetyllysine; alternate.
FT                                {ECO:0000250|UniProtKB:P63038}.
FT   MOD_RES     236    236       N6-succinyllysine; alternate.
FT                                {ECO:0000250|UniProtKB:P63038}.
FT   MOD_RES     249    249       N6-acetyllysine.
FT                                {ECO:0000250|UniProtKB:P63038}.
FT   MOD_RES     250    250       N6-acetyllysine; alternate.
FT                                {ECO:0000250|UniProtKB:P63038}.
FT   MOD_RES     250    250       N6-succinyllysine; alternate.
FT                                {ECO:0000250|UniProtKB:P63038}.
FT   MOD_RES     269    269       N6-acetyllysine.
FT                                {ECO:0000244|PubMed:19608861}.
FT   MOD_RES     292    292       N6-acetyllysine.
FT                                {ECO:0000250|UniProtKB:P63038}.
FT   MOD_RES     301    301       N6-succinyllysine.
FT                                {ECO:0000250|UniProtKB:P63038}.
FT   MOD_RES     314    314       N6-acetyllysine.
FT                                {ECO:0000250|UniProtKB:P63038}.
FT   MOD_RES     352    352       N6-acetyllysine; alternate.
FT                                {ECO:0000244|PubMed:19608861}.
FT   MOD_RES     352    352       N6-succinyllysine; alternate.
FT                                {ECO:0000250|UniProtKB:P63038}.
FT   MOD_RES     359    359       N6-acetyllysine.
FT                                {ECO:0000244|PubMed:19608861}.
FT   MOD_RES     389    389       N6-acetyllysine.
FT                                {ECO:0000250|UniProtKB:P63038}.
FT   MOD_RES     396    396       N6-acetyllysine; alternate.
FT                                {ECO:0000244|PubMed:19608861}.
FT   MOD_RES     396    396       N6-succinyllysine; alternate.
FT                                {ECO:0000250|UniProtKB:P63038}.
FT   MOD_RES     410    410       Phosphoserine.
FT                                {ECO:0000250|UniProtKB:P63038}.
FT   MOD_RES     469    469       N6-acetyllysine.
FT                                {ECO:0000244|PubMed:19608861}.
FT   MOD_RES     481    481       N6-acetyllysine; alternate.
FT                                {ECO:0000250|UniProtKB:P63038}.
FT   MOD_RES     481    481       N6-succinyllysine; alternate.
FT                                {ECO:0000250|UniProtKB:P63038}.
FT   VAR_SEQ     144    158       VMLAVDAVIAELKKQ -> RNVCCHHSVLNFSVL (in
FT                                isoform 2).
FT                                {ECO:0000303|PubMed:14702039}.
FT                                /FTId=VSP_056144.
FT   VAR_SEQ     159    573       Missing (in isoform 2).
FT                                {ECO:0000303|PubMed:14702039}.
FT                                /FTId=VSP_056145.
FT   VARIANT      29     29       D -> G (in HLD4; transfection with the
FT                                mutant protein impairs cell growth that
FT                                worsens with increasing temperature).
FT                                {ECO:0000269|PubMed:18571143}.
FT                                /FTId=VAR_054785.
FT   VARIANT      98     98       V -> I (in SPG13).
FT                                {ECO:0000269|PubMed:11898127}.
FT                                /FTId=VAR_026748.
FT   CONFLICT     67     67       S -> G (in Ref. 2; AAA36022).
FT                                {ECO:0000305}.
FT   CONFLICT    111    111       D -> N (in Ref. 5; BAG35173).
FT                                {ECO:0000305}.
FT   CONFLICT    177    177       N -> S (in Ref. 5; BAG35173).
FT                                {ECO:0000305}.
FT   CONFLICT    202    202       K -> KAS (in Ref. 4; ABB01006).
FT                                {ECO:0000305}.
FT   CONFLICT    260    260       A -> T (in Ref. 5; BAG35173).
FT                                {ECO:0000305}.
FT   STRAND       27     32       {ECO:0000244|PDB:4PJ1}.
FT   HELIX        34     51       {ECO:0000244|PDB:4PJ1}.
FT   HELIX        52     54       {ECO:0000244|PDB:4PJ1}.
FT   STRAND       61     64       {ECO:0000244|PDB:4PJ1}.
FT   STRAND       67     70       {ECO:0000244|PDB:4PJ1}.
FT   STRAND       72     74       {ECO:0000244|PDB:4PJ1}.
FT   HELIX        77     83       {ECO:0000244|PDB:4PJ1}.
FT   HELIX        89    108       {ECO:0000244|PDB:4PJ1}.
FT   HELIX       113    130       {ECO:0000244|PDB:4PJ1}.
FT   HELIX       137    158       {ECO:0000244|PDB:4PJ1}.
FT   HELIX       165    175       {ECO:0000244|PDB:4PJ1}.
FT   TURN        176    178       {ECO:0000244|PDB:4PJ1}.
FT   HELIX       180    193       {ECO:0000244|PDB:4PJ1}.
FT   STRAND      197    203       {ECO:0000244|PDB:4PJ1}.
FT   STRAND      205    208       {ECO:0000244|PDB:4PJ1}.
FT   STRAND      210    214       {ECO:0000244|PDB:4PJ1}.
FT   STRAND      216    220       {ECO:0000244|PDB:4PJ1}.
FT   HELIX       226    228       {ECO:0000244|PDB:4PJ1}.
FT   STRAND      232    235       {ECO:0000244|PDB:4PJ1}.
FT   STRAND      237    249       {ECO:0000244|PDB:4PJ1}.
FT   HELIX       254    266       {ECO:0000244|PDB:4PJ1}.
FT   STRAND      271    276       {ECO:0000244|PDB:4PJ1}.
FT   HELIX       280    292       {ECO:0000244|PDB:4PJ1}.
FT   STRAND      297    301       {ECO:0000244|PDB:4PJ1}.
FT   STRAND      305    307       {ECO:0000244|PDB:4PJ1}.
FT   HELIX       308    320       {ECO:0000244|PDB:4PJ1}.
FT   STRAND      324    326       {ECO:0000244|PDB:4PJ1}.
FT   STRAND      328    330       {ECO:0000244|PDB:4PJ1}.
FT   HELIX       339    341       {ECO:0000244|PDB:4PJ1}.
FT   STRAND      344    350       {ECO:0000244|PDB:4PJ1}.
FT   STRAND      355    360       {ECO:0000244|PDB:4PJ1}.
FT   HELIX       364    378       {ECO:0000244|PDB:4PJ1}.
FT   HELIX       387    396       {ECO:0000244|PDB:4PJ1}.
FT   TURN        397    399       {ECO:0000244|PDB:4PJ1}.
FT   STRAND      401    406       {ECO:0000244|PDB:4PJ1}.
FT   HELIX       411    433       {ECO:0000244|PDB:4PJ1}.
FT   STRAND      436    438       {ECO:0000244|PDB:4PJ1}.
FT   TURN        439    441       {ECO:0000244|PDB:4PJ1}.
FT   HELIX       442    445       {ECO:0000244|PDB:4PJ1}.
FT   HELIX       448    452       {ECO:0000244|PDB:4PJ1}.
FT   HELIX       459    471       {ECO:0000244|PDB:4PJ1}.
FT   HELIX       474    483       {ECO:0000244|PDB:4PJ1}.
FT   HELIX       487    496       {ECO:0000244|PDB:4PJ1}.
FT   STRAND      501    504       {ECO:0000244|PDB:4PJ1}.
FT   TURN        505    508       {ECO:0000244|PDB:4PJ1}.
FT   STRAND      509    512       {ECO:0000244|PDB:4PJ1}.
FT   HELIX       513    516       {ECO:0000244|PDB:4PJ1}.
FT   STRAND      519    521       {ECO:0000244|PDB:4PJ1}.
FT   HELIX       522    539       {ECO:0000244|PDB:4PJ1}.
FT   STRAND      542    548       {ECO:0000244|PDB:4PJ1}.
SQ   SEQUENCE   573 AA;  61055 MW;  E51E1BAD9615899C CRC64;
     MLRLPTVFRQ MRPVSRVLAP HLTRAYAKDV KFGADARALM LQGVDLLADA VAVTMGPKGR
     TVIIEQSWGS PKVTKDGVTV AKSIDLKDKY KNIGAKLVQD VANNTNEEAG DGTTTATVLA
     RSIAKEGFEK ISKGANPVEI RRGVMLAVDA VIAELKKQSK PVTTPEEIAQ VATISANGDK
     EIGNIISDAM KKVGRKGVIT VKDGKTLNDE LEIIEGMKFD RGYISPYFIN TSKGQKCEFQ
     DAYVLLSEKK ISSIQSIVPA LEIANAHRKP LVIIAEDVDG EALSTLVLNR LKVGLQVVAV
     KAPGFGDNRK NQLKDMAIAT GGAVFGEEGL TLNLEDVQPH DLGKVGEVIV TKDDAMLLKG
     KGDKAQIEKR IQEIIEQLDV TTSEYEKEKL NERLAKLSDG VAVLKVGGTS DVEVNEKKDR
     VTDALNATRA AVEEGIVLGG GCALLRCIPA LDSLTPANED QKIGIEIIKR TLKIPAMTIA
     KNAGVEGSLI VEKIMQSSSE VGYDAMAGDF VNMVEKGIID PTKVVRTALL DAAGVASLLT
     TAEVVVTEIP KEEKDPGMGA MGGMGGGMGG GMF
```

|  |
| --- |
| **Mascot:** http://www.matrixscience.com/ |
