# Supplementary material for: Proteomic profiling identifies the inorganic pyrophosphatase (PPA1) protein as a potential biomarker of metastasis in laryngeal squamous cell carcinoma
Source: Amino Acids. 2016 Mar 7;48:1469–76. doi: 10.1007/s00726-016-2201-8 (PMC4875942; doi:10.1007/s00726-016-2201-8)
Supplement: Supplementary file 7 — Supplementary material 7 (HTML 19 kb) [file 726_2016_2201_MOESM7_ESM.html]

Mascot Search Results: GRP75\_HUMAN


# MASCOT Search Results

## Protein View: GRP75\_HUMAN

### Stress-70 protein, mitochondrial OS=Homo sapiens GN=HSPA9 PE=1 SV=2

|  |  |
| --- | --- |
| Database: | SwissProt |
| Score: | 170 |
| Expect: | 2e-13 |
| Nominal mass (Mr): | 73920 |
| Calculated pI: | 5.87 |
| Taxonomy: | Homo sapiens |

Sequence similarity is available as an NCBI BLAST search of GRP75\_HUMAN against nr.

### Search parameters

|  |  |
| --- | --- |
| MS data file: | `peaklist.xml` |
| Enzyme: | Trypsin: cuts C-term side of KR unless next residue is P. |
| Fixed modifications: | Carbamidomethyl (C) |
| Variable modifications: | Oxidation (M) |
|  |  |
| --- | --- |
| Mass values searched: | 36 |
| Mass values matched: | 23 |

### Protein sequence coverage: 41%

Matched peptides shown in ***bold red***.

|  |  |  |  |  |  |
| --- | --- | --- | --- | --- | --- |
| `1` | `MISASRAAAA` | `RLVGAAASRG` | `PTAARHQDSW` | `NGLSHEAFRL` | `VSRRDYASEA` |
| `51` | `IKGAVVGIDL` | `GTTNSCVAVM` | `EGKQAKVLEN` | `AEGARTTPSV` | `VAFTADGERL` |
| `101` | `VGMPAKRQAV` | `TNPNNTFYAT` | `KRLIGRRYDD` | `PEVQKDIKNV` | `PFKIVRASNG` |
| `151` | `DAWVEAHGKL` | `YSPSQIGAFV` | `LMKMKETAEN` | `YLGHTAKNAV` | `ITVPAYFNDS` |
| `201` | `QRQATKDAGQ` | `ISGLNVLRVI` | `NEPTAAALAY` | `GLDKSEDKVI` | `AVYDLGGGTF` |
| `251` | `DISILEIQKG` | `VFEVKSTNGD` | `TFLGGEDFDQ` | `ALLRHIVKEF` | `KRETGVDLTK` |
| `301` | `DNMALQRVRE` | `AAEKAKCELS` | `SSVQTDINLP` | `YLTMDSSGPK` | `HLNMKLTRAQ` |
| `351` | `FEGIVTDLIR` | `RTIAPCQKAM` | `QDAEVSKSDI` | `GEVILVGGMT` | `RMPKVQQTVQ` |
| `401` | `DLFGRAPSKA` | `VNPDEAVAIG` | `AAIQGGVLAG` | `DVTDVLLLDV` | `TPLSLGIETL` |
| `451` | `GGVFTKLINR` | `NTTIPTKKSQ` | `VFSTAADGQT` | `QVEIKVCQGE` | `REMAGDNKLL` |
| `501` | `GQFTLIGIPP` | `APRGVPQIEV` | `TFDIDANGIV` | `HVSAKDKGTG` | `REQQIVIQSS` |
| `551` | `GGLSKDDIEN` | `MVKNAEKYAE` | `EDRRKKERVE` | `AVNMAEGIIH` | `DTETKMEEFK` |
| `601` | `DQLPADECNK` | `LKEEISKMRE` | `LLARKDSETG` | `ENIRQAASSL` | `QQASLKLFEM` |
| `651` | `AYKKMASERE` | `GSGSSGTGEQ` | `KEDQKEEKQ` |  |  |

Unformatted sequence string: 679 residues (for pasting into other applications).

Residue Number

Increasing Mass

Decreasing Mass

| Start | – | End | Observed | Mr(expt) | Mr(calc) | Delta | M | Peptide |
| --- | --- | --- | --- | --- | --- | --- | --- | --- |
| 53 | – | 73 | 2077.9685 | 2076.9612 | 2077.0184 | -0.0572 | 0 | K.GAVVGIDLGTTNSCVAVMEGK.Q |
| 77 | – | 85 | 958.4549 | 957.4476 | 957.4879 | -0.0403 | 0 | K.VLENAEGAR.T |
| 86 | – | 99 | 1450.6970 | 1449.6897 | 1449.7100 | -0.0202 | 0 | R.TTPSVVAFTADGER.L |
| 108 | – | 121 | 1568.7001 | 1567.6928 | 1567.7631 | -0.0703 | 0 | R.QAVTNPNNTFYATK.R |
| 127 | – | 135 | 1149.4944 | 1148.4871 | 1148.5462 | -0.0591 | 1 | R.RYDDPEVQK.D |
| 147 | – | 159 | 1341.5417 | 1340.5345 | 1340.6109 | -0.0764 | 0 | R.ASNGDAWVEAHGK.L |
| 176 | – | 187 | 1333.5786 | 1332.5713 | 1332.6310 | -0.0596 | 0 | K.ETAENYLGHTAK.N |
| 188 | – | 202 | 1694.8324 | 1693.8251 | 1693.8424 | -0.0173 | 0 | K.NAVITVPAYFNDSQR.Q |
| 207 | – | 218 | 1242.6472 | 1241.6399 | 1241.6728 | -0.0328 | 0 | K.DAGQISGLNVLR.V |
| 219 | – | 234 | 1645.8171 | 1644.8099 | 1644.8723 | -0.0624 | 0 | R.VINEPTAAALAYGLDK.S |
| 266 | – | 284 | 2055.9102 | 2054.9029 | 2054.9545 | -0.0516 | 0 | K.STNGDTFLGGEDFDQALLR.H |
| 301 | – | 307 | 847.4125 | 846.4053 | 846.4018 | 0.0035 | 0 | K.DNMALQR.V |
| 349 | – | 360 | 1361.7157 | 1360.7084 | 1360.7351 | -0.0266 | 0 | R.AQFEGIVTDLIR.R |
| 378 | – | 391 | 1446.7437 | 1445.7364 | 1445.7548 | -0.0184 | 0 | K.SDIGEVILVGGMTR.M |
| 378 | – | 391 | 1462.7279 | 1461.7206 | 1461.7497 | -0.0291 | 0 | K.SDIGEVILVGGMTR.M + Oxidation (M) |
| 395 | – | 405 | 1290.6454 | 1289.6381 | 1289.6728 | -0.0347 | 0 | K.VQQTVQDLFGR.A |
| 469 | – | 485 | 1808.8569 | 1807.8497 | 1807.8952 | -0.0456 | 0 | K.SQVFSTAADGQTQVEIK.V |
| 499 | – | 513 | 1592.9404 | 1591.9332 | 1591.9450 | -0.0118 | 0 | K.LLGQFTLIGIPPAPR.G |
| 514 | – | 535 | 2309.1912 | 2308.1839 | 2308.2063 | -0.0224 | 0 | R.GVPQIEVTFDIDANGIVHVSAK.D |
| 568 | – | 574 | 938.3549 | 937.3476 | 937.4253 | -0.0777 | 1 | K.YAEEDRR.K |
| 577 | – | 595 | 2142.0332 | 2141.0259 | 2141.0423 | -0.0163 | 1 | K.ERVEAVNMAEGIIHDTETK.M |
| 579 | – | 595 | 1856.8746 | 1855.8674 | 1855.8986 | -0.0312 | 0 | R.VEAVNMAEGIIHDTETK.M |
| 647 | – | 653 | 901.3527 | 900.3454 | 900.4415 | -0.0961 | 0 | K.LFEMAYK.K |

`No match to: 855.0013, 861.0300, 870.5005, 876.9905, 1034.5808, 1065.9507, 1163.5244, 1411.0421, 2070.9385, 2239.1042, 2273.1382, 2310.1802, 2643.1611`

---

```
AC   Q9UC56;
DT   01-FEB-1995, integrated into UniProtKB/Swiss-Prot.
DT   27-MAY-2002, sequence version 2.
DT   09-DEC-2015, entry version 173.
DE   RecName: Full=Stress-70 protein, mitochondrial;
DE   AltName: Full=75 kDa glucose-regulated protein;
DE            Short=GRP-75;
DE   AltName: Full=Heat shock 70 kDa protein 9;
DE   AltName: Full=Mortalin;
DE            Short=MOT;
DE   AltName: Full=Peptide-binding protein 74;
DE            Short=PBP74;
DE   Flags: Precursor;
GN   Name=HSPA9; Synonyms=GRP75, HSPA9B, mt-HSP70;
OS   Homo sapiens (Human).
OC   Eukaryota; Metazoa; Chordata; Craniata; Vertebrata; Euteleostomi;
OC   Mammalia; Eutheria; Euarchontoglires; Primates; Haplorrhini;
OC   Catarrhini; Hominidae; Homo.
OX   NCBI_TaxID=9606;
RN   [1]
RP   NUCLEOTIDE SEQUENCE [MRNA].
RC   TISSUE=B-cell;
RX   PubMed=7684501;
RA   Domanico S.Z., Denagel D.C., Dahlseid J.N., Green J.M., Pierce S.K.;
RT   "Cloning of the gene encoding peptide-binding protein 74 shows that it
RT   is a new member of the heat shock protein 70 family.";
RL   Mol. Cell. Biol. 13:3598-3610(1993).
RN   [2]
RP   NUCLEOTIDE SEQUENCE [MRNA].
RX   PubMed=7829505; DOI=10.1074/jbc.270.4.1705;
RA   Bhattacharyya T., Karnezis A.N., Murphy S.P., Hoang T., Freeman B.C.,
RA   Phillips B., Morimoto R.I.;
RT   "Cloning and subcellular localization of human mitochondrial hsp70.";
RL   J. Biol. Chem. 270:1705-1710(1995).
RN   [3]
RP   NUCLEOTIDE SEQUENCE [LARGE SCALE MRNA].
RC   TISSUE=Teratocarcinoma;
RX   PubMed=14702039; DOI=10.1038/ng1285;
RA   Ota T., Suzuki Y., Nishikawa T., Otsuki T., Sugiyama T., Irie R.,
RA   Wakamatsu A., Hayashi K., Sato H., Nagai K., Kimura K., Makita H.,
RA   Sekine M., Obayashi M., Nishi T., Shibahara T., Tanaka T., Ishii S.,
RA   Yamamoto J., Saito K., Kawai Y., Isono Y., Nakamura Y., Nagahari K.,
RA   Murakami K., Yasuda T., Iwayanagi T., Wagatsuma M., Shiratori A.,
RA   Sudo H., Hosoiri T., Kaku Y., Kodaira H., Kondo H., Sugawara M.,
RA   Takahashi M., Kanda K., Yokoi T., Furuya T., Kikkawa E., Omura Y.,
RA   Abe K., Kamihara K., Katsuta N., Sato K., Tanikawa M., Yamazaki M.,
RA   Ninomiya K., Ishibashi T., Yamashita H., Murakawa K., Fujimori K.,
RA   Tanai H., Kimata M., Watanabe M., Hiraoka S., Chiba Y., Ishida S.,
RA   Ono Y., Takiguchi S., Watanabe S., Yosida M., Hotuta T., Kusano J.,
RA   Kanehori K., Takahashi-Fujii A., Hara H., Tanase T.-O., Nomura Y.,
RA   Togiya S., Komai F., Hara R., Takeuchi K., Arita M., Imose N.,
RA   Musashino K., Yuuki H., Oshima A., Sasaki N., Aotsuka S.,
RA   Yoshikawa Y., Matsunawa H., Ichihara T., Shiohata N., Sano S.,
RA   Moriya S., Momiyama H., Satoh N., Takami S., Terashima Y., Suzuki O.,
RA   Nakagawa S., Senoh A., Mizoguchi H., Goto Y., Shimizu F., Wakebe H.,
RA   Hishigaki H., Watanabe T., Sugiyama A., Takemoto M., Kawakami B.,
RA   Yamazaki M., Watanabe K., Kumagai A., Itakura S., Fukuzumi Y.,
RA   Fujimori Y., Komiyama M., Tashiro H., Tanigami A., Fujiwara T.,
RA   Ono T., Yamada K., Fujii Y., Ozaki K., Hirao M., Ohmori Y.,
RA   Kawabata A., Hikiji T., Kobatake N., Inagaki H., Ikema Y., Okamoto S.,
RA   Okitani R., Kawakami T., Noguchi S., Itoh T., Shigeta K., Senba T.,
RA   Matsumura K., Nakajima Y., Mizuno T., Morinaga M., Sasaki M.,
RA   Togashi T., Oyama M., Hata H., Watanabe M., Komatsu T.,
RA   Mizushima-Sugano J., Satoh T., Shirai Y., Takahashi Y., Nakagawa K.,
RA   Okumura K., Nagase T., Nomura N., Kikuchi H., Masuho Y., Yamashita R.,
RA   Nakai K., Yada T., Nakamura Y., Ohara O., Isogai T., Sugano S.;
RT   "Complete sequencing and characterization of 21,243 full-length human
RT   cDNAs.";
RL   Nat. Genet. 36:40-45(2004).
RN   [4]
RP   NUCLEOTIDE SEQUENCE [LARGE SCALE MRNA].
RC   TISSUE=Liver;
RA   Suzuki Y., Sugano S., Totoki Y., Toyoda A., Takeda T., Sakaki Y.,
RA   Tanaka A., Yokoyama S.;
RL   Submitted (APR-2005) to the EMBL/GenBank/DDBJ databases.
RN   [5]
RP   NUCLEOTIDE SEQUENCE [GENOMIC DNA], AND VARIANT TYR-184.
RG   NIEHS SNPs program;
RL   Submitted (MAY-2006) to the EMBL/GenBank/DDBJ databases.
RN   [6]
RP   NUCLEOTIDE SEQUENCE [LARGE SCALE GENOMIC DNA].
RA   Mural R.J., Istrail S., Sutton G.G., Florea L., Halpern A.L.,
RA   Mobarry C.M., Lippert R., Walenz B., Shatkay H., Dew I., Miller J.R.,
RA   Flanigan M.J., Edwards N.J., Bolanos R., Fasulo D., Halldorsson B.V.,
RA   Hannenhalli S., Turner R., Yooseph S., Lu F., Nusskern D.R.,
RA   Shue B.C., Zheng X.H., Zhong F., Delcher A.L., Huson D.H.,
RA   Kravitz S.A., Mouchard L., Reinert K., Remington K.A., Clark A.G.,
RA   Waterman M.S., Eichler E.E., Adams M.D., Hunkapiller M.W., Myers E.W.,
RA   Venter J.C.;
RL   Submitted (SEP-2005) to the EMBL/GenBank/DDBJ databases.
RN   [7]
RP   NUCLEOTIDE SEQUENCE [LARGE SCALE MRNA], AND VARIANT ARG-74.
RC   TISSUE=Muscle;
RX   PubMed=15489334; DOI=10.1101/gr.2596504;
RG   The MGC Project Team;
RT   "The status, quality, and expansion of the NIH full-length cDNA
RT   project: the Mammalian Gene Collection (MGC).";
RL   Genome Res. 14:2121-2127(2004).
RN   [8]
RP   PROTEIN SEQUENCE OF 47-68.
RC   TISSUE=Colon carcinoma;
RX   PubMed=9150948; DOI=10.1002/elps.1150180344;
RA   Ji H., Reid G.E., Moritz R.L., Eddes J.S., Burgess A.W., Simpson R.J.;
RT   "A two-dimensional gel database of human colon carcinoma proteins.";
RL   Electrophoresis 18:605-613(1997).
RN   [9]
RP   PROTEIN SEQUENCE OF 47-66.
RC   TISSUE=Mammary gland;
RX   PubMed=7498169; DOI=10.1002/elps.11501601202;
RA   Giometti C.S., Tollaksen S.L., Chubb C., Williams C., Huberman E.;
RT   "Analysis of proteins from human breast epithelial cells using two-
RT   dimensional gel electrophoresis.";
RL   Electrophoresis 16:1215-1224(1995).
RN   [10]
RP   PROTEIN SEQUENCE OF 47-56.
RC   TISSUE=Liver;
RX   PubMed=1286669; DOI=10.1002/elps.11501301201;
RA   Hochstrasser D.F., Frutiger S., Paquet N., Bairoch A., Ravier F.,
RA   Pasquali C., Sanchez J.-C., Tissot J.-D., Bjellqvist B., Vargas R.,
RA   Appel R.D., Hughes G.J.;
RT   "Human liver protein map: a reference database established by
RT   microsequencing and gel comparison.";
RL   Electrophoresis 13:992-1001(1992).
RN   [11]
RP   SEQUENCE REVISION.
RC   TISSUE=Liver;
RX   PubMed=8313870; DOI=10.1002/elps.11501401181;
RA   Hughes G.J., Frutiger S., Paquet N., Pasquali C., Sanchez J.-C.,
RA   Tissot J.-D., Bairoch A., Appel R.D., Hochstrasser D.F.;
RT   "Human liver protein map: update 1993.";
RL   Electrophoresis 14:1216-1222(1993).
RN   [12]
RP   PROTEIN SEQUENCE OF 86-99; 108-121; 160-173; 188-202; 207-234;
RP   349-360; 378-391; 395-405; 469-485; 499-513 AND 542-555, AND
RP   IDENTIFICATION BY MASS SPECTROMETRY.
RC   TISSUE=Brain, Cajal-Retzius cell, and Fetal brain cortex;
RA   Lubec G., Afjehi-Sadat L., Chen W.-Q., Sun Y.;
RL   Submitted (DEC-2008) to UniProtKB.
RN   [13]
RP   INTERACTION WITH FXN.
RX   PubMed=17331979; DOI=10.1093/hmg/ddm038;
RA   Shan Y., Napoli E., Cortopassi G.;
RT   "Mitochondrial frataxin interacts with ISD11 of the NFS1/ISCU complex
RT   and multiple mitochondrial chaperones.";
RL   Hum. Mol. Genet. 16:929-941(2007).
RN   [14]
RP   ACETYLATION [LARGE SCALE ANALYSIS] AT LYS-135; LYS-138; LYS-143;
RP   LYS-234; LYS-288; LYS-300; LYS-567 AND LYS-646, AND IDENTIFICATION BY
RP   MASS SPECTROMETRY [LARGE SCALE ANALYSIS].
RX   PubMed=19608861; DOI=10.1126/science.1175371;
RA   Choudhary C., Kumar C., Gnad F., Nielsen M.L., Rehman M.,
RA   Walther T.C., Olsen J.V., Mann M.;
RT   "Lysine acetylation targets protein complexes and co-regulates major
RT   cellular functions.";
RL   Science 325:834-840(2009).
RN   [15]
RP   INTERACTION WITH HSCB.
RX   PubMed=20668094; DOI=10.1093/hmg/ddq301;
RA   Uhrigshardt H., Singh A., Kovtunovych G., Ghosh M., Rouault T.A.;
RT   "Characterization of the human HSC20, an unusual DnaJ type III
RT   protein, involved in iron-sulfur cluster biogenesis.";
RL   Hum. Mol. Genet. 19:3816-3834(2010).
RN   [16]
RP   IDENTIFICATION BY MASS SPECTROMETRY [LARGE SCALE ANALYSIS].
RX   PubMed=21269460; DOI=10.1186/1752-0509-5-17;
RA   Burkard T.R., Planyavsky M., Kaupe I., Breitwieser F.P.,
RA   Buerckstuemmer T., Bennett K.L., Superti-Furga G., Colinge J.;
RT   "Initial characterization of the human central proteome.";
RL   BMC Syst. Biol. 5:17-17(2011).
RN   [17]
RP   MALONYLATION AT LYS-206.
RX   PubMed=21908771; DOI=10.1074/mcp.M111.012658;
RA   Peng C., Lu Z., Xie Z., Cheng Z., Chen Y., Tan M., Luo H., Zhang Y.,
RA   He W., Yang K., Zwaans B.M., Tishkoff D., Ho L., Lombard D., He T.C.,
RA   Dai J., Verdin E., Ye Y., Zhao Y.;
RT   "The first identification of lysine malonylation substrates and its
RT   regulatory enzyme.";
RL   Mol. Cell. Proteomics 10:M111.012658.01-M111.012658.12(2011).
RN   [18]
RP   IDENTIFICATION IN THE MINOS/MITOS COMPLEX.
RX   PubMed=22114354; DOI=10.1091/mbc.E11-09-0774;
RA   Alkhaja A.K., Jans D.C., Nikolov M., Vukotic M., Lytovchenko O.,
RA   Ludewig F., Schliebs W., Riedel D., Urlaub H., Jakobs S., Deckers M.;
RT   "MINOS1 is a conserved component of mitofilin complexes and required
RT   for mitochondrial function and cristae organization.";
RL   Mol. Biol. Cell 23:247-257(2012).
RN   [19]
RP   SUBCELLULAR LOCATION [LARGE SCALE ANALYSIS].
RX   PubMed=22002106; DOI=10.1074/mcp.M111.013680;
RA   Ahmad Y., Boisvert F.M., Lundberg E., Uhlen M., Lamond A.I.;
RT   "Systematic analysis of protein pools, isoforms, and modifications
RT   affecting turnover and subcellular localization.";
RL   Mol. Cell. Proteomics 11:M111.013680.01-M111.013680.15(2012).
RN   [20]
RP   FUNCTION, AND INTERACTION WITH TESPA.
RX   PubMed=23501103; DOI=10.1016/j.bbrc.2013.02.099;
RA   Matsuzaki H., Fujimoto T., Tanaka M., Shirasawa S.;
RT   "Tespa1 is a novel component of mitochondria-associated endoplasmic
RT   reticulum membranes and affects mitochondrial calcium flux.";
RL   Biochem. Biophys. Res. Commun. 433:322-326(2013).
RN   [21]
RP   INTERACTION WITH DNLZ.
RX   PubMed=23462535; DOI=10.1016/j.ijbiomac.2013.02.009;
RA   Dores-Silva P.R., Minari K., Ramos C.H., Barbosa L.R., Borges J.C.;
RT   "Structural and stability studies of the human mtHsp70-escort protein
RT   1: An essential mortalin co-chaperone.";
RL   Int. J. Biol. Macromol. 56:140-148(2013).
RN   [22]
RP   IDENTIFICATION BY MASS SPECTROMETRY [LARGE SCALE ANALYSIS].
RC   TISSUE=Liver;
RX   PubMed=24275569; DOI=10.1016/j.jprot.2013.11.014;
RA   Bian Y., Song C., Cheng K., Dong M., Wang F., Huang J., Sun D.,
RA   Wang L., Ye M., Zou H.;
RT   "An enzyme assisted RP-RPLC approach for in-depth analysis of human
RT   liver phosphoproteome.";
RL   J. Proteomics 96:253-262(2014).
RN   [23]
RP   IDENTIFICATION BY MASS SPECTROMETRY [LARGE SCALE ANALYSIS].
RX   PubMed=25944712; DOI=10.1002/pmic.201400617;
RA   Vaca Jacome A.S., Rabilloud T., Schaeffer-Reiss C., Rompais M.,
RA   Ayoub D., Lane L., Bairoch A., Van Dorsselaer A., Carapito C.;
RT   "N-terminome analysis of the human mitochondrial proteome.";
RL   Proteomics 15:2519-2524(2015).
CC   -!- FUNCTION: Implicated in the control of cell proliferation and
CC       cellular aging. May also act as a chaperone.
CC       {ECO:0000269|PubMed:23501103}.
CC   -!- SUBUNIT: Interacts with FXN. Interacts with HSCB. Associates with
CC       the mitochondrial contact site and cristae organizing system
CC       (MICOS) complex, composed of at least MINOS1/MIC10, CHCHD3/MIC19,
CC       CHCHD6/MIC25, APOOL/MIC27, IMMT/MIC60, APOO/MIC23/MIC26 and
CC       QIL1/MIC13. This complex was also known under the names MINOS or
CC       MitOS complex. The MICOS complex associates with mitochondrial
CC       outer membrane proteins SAMM50, MTX1, MTX2 and DNAJC11,
CC       mitochondrial inner membrane protein TMEM11 and with HSPA9.
CC       Interacts with DNLZ, the interaction is required to prevent self-
CC       aggregation. Interacts with TESPA1. {ECO:0000269|PubMed:17331979,
CC       ECO:0000269|PubMed:20668094, ECO:0000269|PubMed:22114354,
CC       ECO:0000269|PubMed:23462535, ECO:0000269|PubMed:23501103}.
CC   -!- INTERACTION:
CC       P00533:EGFR; NbExp=4; IntAct=EBI-354932, EBI-297353;
CC       Q8WX92:NELFB; NbExp=2; IntAct=EBI-354932, EBI-347721;
CC       P04637:TP53; NbExp=6; IntAct=EBI-354932, EBI-366083;
CC       O15350:TP73; NbExp=11; IntAct=EBI-354932, EBI-389606;
CC       A4D2J0:YKT6; NbExp=3; IntAct=EBI-354932, EBI-10173443;
CC   -!- SUBCELLULAR LOCATION: Mitochondrion {ECO:0000269|PubMed:22002106}.
CC       Nucleus, nucleolus {ECO:0000269|PubMed:22002106}.
CC   -!- SIMILARITY: Belongs to the heat shock protein 70 family.
CC       {ECO:0000305}.
CC   -!- WEB RESOURCE: Name=NIEHS-SNPs;
CC       URL="http://egp.gs.washington.edu/data/hspa9b/";
DR   EMBL; L11066; -; NOT_ANNOTATED_CDS; mRNA.
DR   EMBL; L15189; AAA67526.1; -; mRNA.
DR   EMBL; AK315177; BAG37618.1; -; mRNA.
DR   EMBL; AK222758; BAD96478.1; -; mRNA.
DR   EMBL; DQ531046; ABF50973.1; -; Genomic_DNA.
DR   EMBL; CH471062; EAW62129.1; -; Genomic_DNA.
DR   EMBL; BC000478; AAH00478.1; -; mRNA.
DR   EMBL; BC024034; AAH24034.1; -; mRNA.
DR   CCDS; CCDS4208.1; -.
DR   PIR; B48127; B48127.
DR   RefSeq; NP_004125.3; NM_004134.6.
DR   UniGene; Hs.184233; -.
DR   PDB; 3N8E; X-ray; 2.80 A; A/B=439-597.
DR   PDB; 4KBO; X-ray; 2.80 A; A=52-431.
DR   PDBsum; 3N8E; -.
DR   PDBsum; 4KBO; -.
DR   ProteinModelPortal; P38646; -.
DR   SMR; P38646; 54-651.
DR   BioGrid; 109545; 193.
DR   IntAct; P38646; 51.
DR   MINT; MINT-1143092; -.
DR   STRING; 9606.ENSP00000297185; -.
DR   PhosphoSite; P38646; -.
DR   BioMuta; HSPA9; -.
DR   DMDM; 21264428; -.
DR   DOSAC-COBS-2DPAGE; P38646; -.
DR   OGP; P38646; -.
DR   REPRODUCTION-2DPAGE; IPI00007765; -.
DR   SWISS-2DPAGE; P38646; -.
DR   UCD-2DPAGE; P38646; -.
DR   MaxQB; P38646; -.
DR   PaxDb; P38646; -.
DR   PRIDE; P38646; -.
DR   DNASU; 3313; -.
DR   Ensembl; ENST00000297185; ENSP00000297185; ENSG00000113013.
DR   GeneID; 3313; -.
DR   KEGG; hsa:3313; -.
DR   UCSC; uc003ldf.3; human.
DR   CTD; 3313; -.
DR   GeneCards; HSPA9; -.
DR   HGNC; HGNC:5244; HSPA9.
DR   HPA; CAB005219; -.
DR   HPA; HPA000898; -.
DR   MIM; 600548; gene.
DR   neXtProt; NX_P38646; -.
DR   PharmGKB; PA162391712; -.
DR   eggNOG; KOG0102; Eukaryota.
DR   eggNOG; COG0443; LUCA.
DR   GeneTree; ENSGT00820000127001; -.
DR   HOVERGEN; HBG051845; -.
DR   InParanoid; P38646; -.
DR   KO; K04043; -.
DR   OMA; EKMAPPQ; -.
DR   OrthoDB; EOG715Q3K; -.
DR   PhylomeDB; P38646; -.
DR   TreeFam; TF105046; -.
DR   Reactome; R-HSA-1268020; Mitochondrial protein import.
DR   Reactome; R-HSA-3371453; Regulation of HSF1-mediated heat shock response.
DR   ChiTaRS; HSPA9; human.
DR   EvolutionaryTrace; P38646; -.
DR   GeneWiki; HSPA9; -.
DR   GenomeRNAi; 3313; -.
DR   NextBio; 13142; -.
DR   PRO; PR:P38646; -.
DR   Proteomes; UP000005640; Chromosome 5.
DR   Bgee; P38646; -.
DR   ExpressionAtlas; P38646; baseline and differential.
DR   Genevisible; P38646; HS.
DR   GO; GO:0005737; C:cytoplasm; TAS:ProtInc.
DR   GO; GO:0070062; C:extracellular exosome; IDA:UniProtKB.
DR   GO; GO:0005925; C:focal adhesion; IDA:UniProtKB.
DR   GO; GO:0042645; C:mitochondrial nucleoid; IDA:BHF-UCL.
DR   GO; GO:0005739; C:mitochondrion; TAS:UniProtKB.
DR   GO; GO:0043209; C:myelin sheath; IEA:Ensembl.
DR   GO; GO:0005730; C:nucleolus; IEA:UniProtKB-SubCell.
DR   GO; GO:0005524; F:ATP binding; IEA:UniProtKB-KW.
DR   GO; GO:0044822; F:poly(A) RNA binding; IDA:UniProtKB.
DR   GO; GO:0031625; F:ubiquitin protein ligase binding; IPI:ParkinsonsUK-UCL.
DR   GO; GO:0051082; F:unfolded protein binding; TAS:UniProtKB.
DR   GO; GO:0044267; P:cellular protein metabolic process; TAS:Reactome.
DR   GO; GO:0071347; P:cellular response to interleukin-1; IEA:Ensembl.
DR   GO; GO:0043066; P:negative regulation of apoptotic process; TAS:UniProtKB.
DR   GO; GO:0006611; P:protein export from nucleus; IEA:Ensembl.
DR   GO; GO:0006457; P:protein folding; IEA:InterPro.
DR   GO; GO:0006626; P:protein targeting to mitochondrion; TAS:Reactome.
DR   GO; GO:0009636; P:response to toxic substance; IEA:Ensembl.
DR   Gene3D; 1.20.1270.10; -; 1.
DR   Gene3D; 2.60.34.10; -; 1.
DR   HAMAP; MF_00332; DnaK; 1.
DR   InterPro; IPR012725; Chaperone_DnaK.
DR   InterPro; IPR018181; Heat_shock_70_CS.
DR   InterPro; IPR029048; HSP70_C.
DR   InterPro; IPR029047; HSP70_peptide-bd.
DR   InterPro; IPR013126; Hsp_70_fam.
DR   Pfam; PF00012; HSP70; 1.
DR   PRINTS; PR00301; HEATSHOCK70.
DR   SUPFAM; SSF100920; SSF100920; 1.
DR   TIGRFAMs; TIGR02350; prok_dnaK; 1.
DR   PROSITE; PS00297; HSP70_1; 1.
DR   PROSITE; PS00329; HSP70_2; 1.
DR   PROSITE; PS01036; HSP70_3; 1.
PE   1: Evidence at protein level;
KW   3D-structure; Acetylation; ATP-binding; Chaperone; Complete proteome;
KW   Direct protein sequencing; Mitochondrion; Nucleotide-binding; Nucleus;
KW   Polymorphism; Reference proteome; Transit peptide.
FT   TRANSIT       1     46       Mitochondrion.
FT                                {ECO:0000269|PubMed:1286669,
FT                                ECO:0000269|PubMed:7498169,
FT                                ECO:0000269|PubMed:9150948}.
FT   CHAIN        47    679       Stress-70 protein, mitochondrial.
FT                                /FTId=PRO_0000013563.
FT   MOD_RES      76     76       N6-acetyllysine.
FT                                {ECO:0000250|UniProtKB:P38647}.
FT   MOD_RES     135    135       N6-acetyllysine; alternate.
FT                                {ECO:0000244|PubMed:19608861}.
FT   MOD_RES     135    135       N6-succinyllysine; alternate.
FT                                {ECO:0000250|UniProtKB:P38647}.
FT   MOD_RES     138    138       N6-acetyllysine; alternate.
FT                                {ECO:0000244|PubMed:19608861}.
FT   MOD_RES     138    138       N6-succinyllysine; alternate.
FT                                {ECO:0000250|UniProtKB:P38647}.
FT   MOD_RES     143    143       N6-acetyllysine.
FT                                {ECO:0000244|PubMed:19608861}.
FT   MOD_RES     206    206       N6-acetyllysine; alternate.
FT                                {ECO:0000250|UniProtKB:P38647}.
FT   MOD_RES     206    206       N6-malonyllysine; alternate.
FT                                {ECO:0000269|PubMed:21908771}.
FT   MOD_RES     206    206       N6-succinyllysine; alternate.
FT                                {ECO:0000250|UniProtKB:P38647}.
FT   MOD_RES     234    234       N6-acetyllysine.
FT                                {ECO:0000244|PubMed:19608861}.
FT   MOD_RES     288    288       N6-acetyllysine.
FT                                {ECO:0000244|PubMed:19608861}.
FT   MOD_RES     300    300       N6-acetyllysine; alternate.
FT                                {ECO:0000244|PubMed:19608861}.
FT   MOD_RES     300    300       N6-succinyllysine; alternate.
FT                                {ECO:0000250|UniProtKB:P38647}.
FT   MOD_RES     368    368       N6-succinyllysine.
FT                                {ECO:0000250|UniProtKB:P38647}.
FT   MOD_RES     394    394       N6-succinyllysine.
FT                                {ECO:0000250|UniProtKB:P38647}.
FT   MOD_RES     567    567       N6-acetyllysine; alternate.
FT                                {ECO:0000244|PubMed:19608861}.
FT   MOD_RES     567    567       N6-succinyllysine; alternate.
FT                                {ECO:0000250|UniProtKB:P38647}.
FT   MOD_RES     600    600       N6-acetyllysine; alternate.
FT                                {ECO:0000250|UniProtKB:P38647}.
FT   MOD_RES     600    600       N6-succinyllysine; alternate.
FT                                {ECO:0000250|UniProtKB:P38647}.
FT   MOD_RES     610    610       N6-succinyllysine.
FT                                {ECO:0000250|UniProtKB:P38647}.
FT   MOD_RES     612    612       N6-acetyllysine.
FT                                {ECO:0000250|UniProtKB:P38647}.
FT   MOD_RES     646    646       N6-acetyllysine; alternate.
FT                                {ECO:0000244|PubMed:19608861}.
FT   MOD_RES     646    646       N6-succinyllysine; alternate.
FT                                {ECO:0000250|UniProtKB:P38647}.
FT   VARIANT      74     74       Q -> R (in dbSNP:rs17856004).
FT                                {ECO:0000269|PubMed:15489334}.
FT                                /FTId=VAR_046482.
FT   VARIANT     127    127       R -> G (in dbSNP:rs35091799).
FT                                /FTId=VAR_049622.
FT   VARIANT     184    184       H -> Y. {ECO:0000269|Ref.5}.
FT                                /FTId=VAR_046483.
FT   VARIANT     225    225       A -> G (in dbSNP:rs34558740).
FT                                /FTId=VAR_049623.
FT   CONFLICT     48     48       S -> P (in Ref. 9; AA sequence).
FT                                {ECO:0000305}.
FT   CONFLICT     66     66       C -> S (in Ref. 9; AA sequence).
FT                                {ECO:0000305}.
FT   CONFLICT    176    176       E -> V (in Ref. 3; BAG37618).
FT                                {ECO:0000305}.
FT   CONFLICT    184    184       H -> R (in Ref. 7; AAH00478/AAH24034).
FT                                {ECO:0000305}.
FT   CONFLICT    249    249       T -> A (in Ref. 4; BAD96478).
FT                                {ECO:0000305}.
FT   CONFLICT    385    385       L -> P (in Ref. 4; BAD96478).
FT                                {ECO:0000305}.
FT   CONFLICT    540    540       G -> R (in Ref. 2; AAA67526).
FT                                {ECO:0000305}.
FT   STRAND       56     59       {ECO:0000244|PDB:4KBO}.
FT   STRAND       62     71       {ECO:0000244|PDB:4KBO}.
FT   STRAND       74     77       {ECO:0000244|PDB:4KBO}.
FT   STRAND       91     93       {ECO:0000244|PDB:4KBO}.
FT   STRAND       99    102       {ECO:0000244|PDB:4KBO}.
FT   HELIX       103    106       {ECO:0000244|PDB:4KBO}.
FT   TURN        107    111       {ECO:0000244|PDB:4KBO}.
FT   HELIX       113    115       {ECO:0000244|PDB:4KBO}.
FT   HELIX       120    122       {ECO:0000244|PDB:4KBO}.
FT   TURN        123    125       {ECO:0000244|PDB:4KBO}.
FT   HELIX       131    139       {ECO:0000244|PDB:4KBO}.
FT   STRAND      141    146       {ECO:0000244|PDB:4KBO}.
FT   STRAND      148    156       {ECO:0000244|PDB:4KBO}.
FT   STRAND      159    161       {ECO:0000244|PDB:4KBO}.
FT   HELIX       163    182       {ECO:0000244|PDB:4KBO}.
FT   STRAND      188    193       {ECO:0000244|PDB:4KBO}.
FT   HELIX       199    211       {ECO:0000244|PDB:4KBO}.
FT   STRAND      215    221       {ECO:0000244|PDB:4KBO}.
FT   HELIX       222    229       {ECO:0000244|PDB:4KBO}.
FT   HELIX       232    234       {ECO:0000244|PDB:4KBO}.
FT   STRAND      236    245       {ECO:0000244|PDB:4KBO}.
FT   STRAND      250    258       {ECO:0000244|PDB:4KBO}.
FT   STRAND      261    270       {ECO:0000244|PDB:4KBO}.
FT   HELIX       275    294       {ECO:0000244|PDB:4KBO}.
FT   HELIX       302    318       {ECO:0000244|PDB:4KBO}.
FT   TURN        319    321       {ECO:0000244|PDB:4KBO}.
FT   STRAND      323    333       {ECO:0000244|PDB:4KBO}.
FT   STRAND      340    347       {ECO:0000244|PDB:4KBO}.
FT   HELIX       348    354       {ECO:0000244|PDB:4KBO}.
FT   HELIX       356    360       {ECO:0000244|PDB:4KBO}.
FT   HELIX       363    372       {ECO:0000244|PDB:4KBO}.
FT   TURN        377    379       {ECO:0000244|PDB:4KBO}.
FT   STRAND      382    387       {ECO:0000244|PDB:4KBO}.
FT   HELIX       388    391       {ECO:0000244|PDB:4KBO}.
FT   HELIX       393    403       {ECO:0000244|PDB:4KBO}.
FT   TURN        413    415       {ECO:0000244|PDB:4KBO}.
FT   HELIX       416    428       {ECO:0000244|PDB:4KBO}.
FT   STRAND      445    448       {ECO:0000244|PDB:3N8E}.
FT   STRAND      452    458       {ECO:0000244|PDB:3N8E}.
FT   STRAND      463    472       {ECO:0000244|PDB:3N8E}.
FT   STRAND      482    490       {ECO:0000244|PDB:3N8E}.
FT   HELIX       494    496       {ECO:0000244|PDB:3N8E}.
FT   STRAND      497    505       {ECO:0000244|PDB:3N8E}.
FT   STRAND      518    524       {ECO:0000244|PDB:3N8E}.
FT   STRAND      530    536       {ECO:0000244|PDB:3N8E}.
FT   TURN        537    539       {ECO:0000244|PDB:3N8E}.
FT   STRAND      542    548       {ECO:0000244|PDB:3N8E}.
FT   HELIX       555    567       {ECO:0000244|PDB:3N8E}.
FT   HELIX       569    590       {ECO:0000244|PDB:3N8E}.
SQ   SEQUENCE   679 AA;  73680 MW;  90969A8D06757753 CRC64;
     MISASRAAAA RLVGAAASRG PTAARHQDSW NGLSHEAFRL VSRRDYASEA IKGAVVGIDL
     GTTNSCVAVM EGKQAKVLEN AEGARTTPSV VAFTADGERL VGMPAKRQAV TNPNNTFYAT
     KRLIGRRYDD PEVQKDIKNV PFKIVRASNG DAWVEAHGKL YSPSQIGAFV LMKMKETAEN
     YLGHTAKNAV ITVPAYFNDS QRQATKDAGQ ISGLNVLRVI NEPTAAALAY GLDKSEDKVI
     AVYDLGGGTF DISILEIQKG VFEVKSTNGD TFLGGEDFDQ ALLRHIVKEF KRETGVDLTK
     DNMALQRVRE AAEKAKCELS SSVQTDINLP YLTMDSSGPK HLNMKLTRAQ FEGIVTDLIR
     RTIAPCQKAM QDAEVSKSDI GEVILVGGMT RMPKVQQTVQ DLFGRAPSKA VNPDEAVAIG
     AAIQGGVLAG DVTDVLLLDV TPLSLGIETL GGVFTKLINR NTTIPTKKSQ VFSTAADGQT
     QVEIKVCQGE REMAGDNKLL GQFTLIGIPP APRGVPQIEV TFDIDANGIV HVSAKDKGTG
     REQQIVIQSS GGLSKDDIEN MVKNAEKYAE EDRRKKERVE AVNMAEGIIH DTETKMEEFK
     DQLPADECNK LKEEISKMRE LLARKDSETG ENIRQAASSL QQASLKLFEM AYKKMASERE
     GSGSSGTGEQ KEDQKEEKQ
```

|  |
| --- |
| **Mascot:** http://www.matrixscience.com/ |
