# Supplementary material for: Proteomic profiling identifies the inorganic pyrophosphatase (PPA1) protein as a potential biomarker of metastasis in laryngeal squamous cell carcinoma
Source: Amino Acids. 2016 Mar 7;48:1469–76. doi: 10.1007/s00726-016-2201-8 (PMC4875942; doi:10.1007/s00726-016-2201-8)
Supplement: Supplementary file 8 — Supplementary material 8 (HTML 10 kb) [file 726_2016_2201_MOESM8_ESM.html]

Mascot Search Results: PDIA3\_HUMAN


# MASCOT Search Results

## Protein View: PDIA3\_HUMAN

### Protein disulfide-isomerase A3 OS=Homo sapiens GN=PDIA3 PE=1 SV=4

|  |  |
| --- | --- |
| Database: | SwissProt |
| Score: | 85 |
| Expect: | 6.5e-05 |
| Nominal mass (Mr): | 57146 |
| Calculated pI: | 5.98 |
| Taxonomy: | Homo sapiens |

Sequence similarity is available as an NCBI BLAST search of PDIA3\_HUMAN against nr.

### Search parameters

|  |  |
| --- | --- |
| MS data file: | `peaklist.xml` |
| Enzyme: | Trypsin: cuts C-term side of KR unless next residue is P. |
| Fixed modifications: | Carbamidomethyl (C) |
| Variable modifications: | Oxidation (M) |
|  |  |
| --- | --- |
| Mass values searched: | 27 |
| Mass values matched: | 12 |

### Protein sequence coverage: 32%

Matched peptides shown in ***bold red***.

|  |  |  |  |  |  |
| --- | --- | --- | --- | --- | --- |
| `1` | `MRLRRLALFP` | `GVALLLAAAR` | `LAAASDVLEL` | `TDDNFESRIS` | `DTGSAGLMLV` |
| `51` | `EFFAPWCGHC` | `KRLAPEYEAA` | `ATRLKGIVPL` | `AKVDCTANTN` | `TCNKYGVSGY` |
| `101` | `PTLKIFRDGE` | `EAGAYDGPRT` | `ADGIVSHLKK` | `QAGPASVPLR` | `TEEEFKKFIS` |
| `151` | `DKDASIVGFF` | `DDSFSEAHSE` | `FLKAASNLRD` | `NYRFAHTNVE` | `SLVNEYDDNG` |
| `201` | `EGIILFRPSH` | `LTNKFEDKTV` | `AYTEQKMTSG` | `KIKKFIQENI` | `FGICPHMTED` |
| `251` | `NKDLIQGKDL` | `LIAYYDVDYE` | `KNAKGSNYWR` | `NRVMMVAKKF` | `LDAGHKLNFA` |
| `301` | `VASRKTFSHE` | `LSDFGLESTA` | `GEIPVVAIRT` | `AKGEKFVMQE` | `EFSRDGKALE` |
| `351` | `RFLQDYFDGN` | `LKRYLKSEPI` | `PESNDGPVKV` | `VVAENFDEIV` | `NNENKDVLIE` |
| `401` | `FYAPWCGHCK` | `NLEPKYKELG` | `EKLSKDPNIV` | `IAKMDATAND` | `VPSPYEVRGF` |
| `451` | `PTIYFSPANK` | `KLNPKKYEGG` | `RELSDFISYL` | `QREATNPPVI` | `QEEKPKKKKK` |
| `501` | `AQEDL` |  |  |  |  |

Unformatted sequence string: 505 residues (for pasting into other applications).

Residue Number

Increasing Mass

Decreasing Mass

| Start | – | End | Observed | Mr(expt) | Mr(calc) | Delta | M | Peptide |
| --- | --- | --- | --- | --- | --- | --- | --- | --- |
| 63 | – | 73 | 1191.6581 | 1190.6508 | 1190.5931 | 0.0577 | 0 | R.LAPEYEAAATR.L |
| 108 | – | 119 | 1236.5645 | 1235.5572 | 1235.5054 | 0.0517 | 0 | R.DGEEAGAYDGPR.T |
| 153 | – | 173 | 2348.2407 | 2347.2334 | 2347.0645 | 0.1690 | 0 | K.DASIVGFFDDSFSEAHSEFLK.A |
| 259 | – | 271 | 1619.8237 | 1618.8165 | 1618.7766 | 0.0398 | 0 | K.DLLIAYYDVDYEK.N |
| 297 | – | 304 | 877.2624 | 876.2551 | 876.4817 | -0.2266 | 0 | K.LNFAVASR.K |
| 306 | – | 329 | 2575.5000 | 2574.4927 | 2574.2966 | 0.1961 | 0 | K.TFSHELSDFGLESTAGEIPVVAIR.T |
| 336 | – | 344 | 1172.6238 | 1171.6165 | 1171.5332 | 0.0833 | 0 | K.FVMQEEFSR.D |
| 352 | – | 362 | 1359.6969 | 1358.6896 | 1358.6507 | 0.0390 | 0 | R.FLQDYFDGNLK.R |
| 380 | – | 395 | 1832.9661 | 1831.9588 | 1831.8952 | 0.0636 | 0 | K.VVVAENFDEIVNNENK.D |
| 434 | – | 448 | 1664.8173 | 1663.8100 | 1663.7512 | 0.0588 | 0 | K.MDATANDVPSPYEVR.G |
| 449 | – | 460 | 1341.7059 | 1340.6987 | 1340.6765 | 0.0222 | 0 | R.GFPTIYFSPANK.K |
| 472 | – | 482 | 1370.7614 | 1369.7541 | 1369.6878 | 0.0663 | 0 | R.ELSDFISYLQR.E |

`No match to: 825.4205, 839.2819, 841.3239, 845.3185, 855.2766, 861.2904, 893.2322, 1066.2281, 1474.7582, 1639.8208, 2239.2571, 2371.2629, 2397.3223, 2590.5425, 2752.5425`

---

```
AC   P30101; Q13453; Q14255; Q8IYF8; Q9UMU7;
DT   01-APR-1993, integrated into UniProtKB/Swiss-Prot.
DT   01-NOV-1997, sequence version 4.
DT   09-DEC-2015, entry version 189.
DE   RecName: Full=Protein disulfide-isomerase A3;
DE            EC=5.3.4.1;
DE   AltName: Full=58 kDa glucose-regulated protein;
DE   AltName: Full=58 kDa microsomal protein;
DE            Short=p58;
DE   AltName: Full=Disulfide isomerase ER-60;
DE   AltName: Full=Endoplasmic reticulum resident protein 57;
DE            Short=ER protein 57;
DE            Short=ERp57;
DE   AltName: Full=Endoplasmic reticulum resident protein 60;
DE            Short=ER protein 60;
DE            Short=ERp60;
DE   Flags: Precursor;
GN   Name=PDIA3; Synonyms=ERP57, ERP60, GRP58;
OS   Homo sapiens (Human).
OC   Eukaryota; Metazoa; Chordata; Craniata; Vertebrata; Euteleostomi;
OC   Mammalia; Eutheria; Euarchontoglires; Primates; Haplorrhini;
OC   Catarrhini; Hominidae; Homo.
OX   NCBI_TaxID=9606;
RN   [1]
RP   NUCLEOTIDE SEQUENCE [MRNA].
RX   PubMed=7945384; DOI=10.1006/bbrc.1994.2469;
RA   Hirano N., Shibasaki F., Katoh H., Sakai R., Tanaka T., Nishida J.,
RA   Yazaki Y., Takenawa T., Hirai H.;
RT   "Molecular cloning and characterization of a cDNA for bovine
RT   phospholipase C-alpha: proposal of redesignation of phospholipase C-
RT   alpha.";
RL   Biochem. Biophys. Res. Commun. 204:375-382(1994).
RN   [2]
RP   NUCLEOTIDE SEQUENCE [MRNA], PROTEIN SEQUENCE OF 25-36 AND 130-144, AND
RP   CATALYTIC ACTIVITY.
RC   TISSUE=Liver;
RX   PubMed=7487104; DOI=10.1006/abbi.1995.0060;
RA   Bourdi M., Demady D., Martin J.L., Jabbour S.K., Martin B.M.,
RA   George J.W., Pohl L.R.;
RT   "cDNA cloning and baculovirus expression of the human liver
RT   endoplasmic reticulum P58: characterization as a protein disulfide
RT   isomerase isoform, but not as a protease or a carnitine
RT   acyltransferase.";
RL   Arch. Biochem. Biophys. 323:397-403(1995).
RN   [3]
RP   NUCLEOTIDE SEQUENCE [MRNA].
RC   TISSUE=Heart;
RX   PubMed=8687406;
RA   Koivunen P., Helaakoski T., Annunen P., Veijola J., Raeisaenen S.,
RA   Pihlajaniemi T., Kivirikko K.I.;
RT   "ERp60 does not substitute for protein disulphide isomerase as the
RT   beta-subunit of prolyl 4-hydroxylase.";
RL   Biochem. J. 316:599-605(1996).
RN   [4]
RP   NUCLEOTIDE SEQUENCE [MRNA].
RC   TISSUE=Placenta;
RX   PubMed=8624847; DOI=10.1016/1357-2725(95)00120-4;
RA   Charnock-Jones D.S., Day K., Smith S.K.;
RT   "Cloning, expression and genomic organization of human placental
RT   protein disulfide isomerase (previously identified as phospholipase C
RT   alpha).";
RL   Int. J. Biochem. Cell Biol. 28:81-89(1996).
RN   [5]
RP   NUCLEOTIDE SEQUENCE [GENOMIC DNA].
RX   PubMed=9205111; DOI=10.1006/geno.1997.4750;
RA   Koivunen P., Horelli-Kuitunen N., Helaakoski T., Karvonen P.,
RA   Jaakkola M., Palotie A., Kivirikko K.I.;
RT   "Structures of the human gene for the protein disulfide isomerase-
RT   related polypeptide ERp60 and a processed gene and assignment of these
RT   genes to 15q15 and 1q21.";
RL   Genomics 42:397-404(1997).
RN   [6]
RP   NUCLEOTIDE SEQUENCE [MRNA], PROTEIN SEQUENCE OF 25-34 AND 504-505, AND
RP   MUTAGENESIS OF CYS-57; CYS-60; CYS-406 AND CYS-409.
RC   TISSUE=Liver epithelium;
RX   PubMed=9399589; DOI=10.1093/oxfordjournals.jbchem.a021830;
RA   Urade R., Oda T., Ito H., Moriyama T., Utsumi S., Kito M.;
RT   "Functions of characteristic Cys-Gly-His-Cys (CGHC) and Gln-Glu-Asp-
RT   Leu (QEDL) motifs of microsomal ER-60 protease.";
RL   J. Biochem. 122:834-842(1997).
RN   [7]
RP   NUCLEOTIDE SEQUENCE [LARGE SCALE MRNA].
RC   TISSUE=Liver, Lung, and Lymph;
RX   PubMed=15489334; DOI=10.1101/gr.2596504;
RG   The MGC Project Team;
RT   "The status, quality, and expansion of the NIH full-length cDNA
RT   project: the Mammalian Gene Collection (MGC).";
RL   Genome Res. 14:2121-2127(2004).
RN   [8]
RP   PROTEIN SEQUENCE OF 25-54; 62-75 AND 95-104.
RC   TISSUE=Colon carcinoma;
RX   PubMed=9150948; DOI=10.1002/elps.1150180344;
RA   Ji H., Reid G.E., Moritz R.L., Eddes J.S., Burgess A.W., Simpson R.J.;
RT   "A two-dimensional gel database of human colon carcinoma proteins.";
RL   Electrophoresis 18:605-613(1997).
RN   [9]
RP   PROTEIN SEQUENCE OF 25-38.
RC   TISSUE=Platelet;
RX   PubMed=12665801; DOI=10.1038/nbt810;
RA   Gevaert K., Goethals M., Martens L., Van Damme J., Staes A.,
RA   Thomas G.R., Vandekerckhove J.;
RT   "Exploring proteomes and analyzing protein processing by mass
RT   spectrometric identification of sorted N-terminal peptides.";
RL   Nat. Biotechnol. 21:566-569(2003).
RN   [10]
RP   PROTEIN SEQUENCE OF 25-33.
RC   TISSUE=Liver;
RX   PubMed=1286669; DOI=10.1002/elps.11501301201;
RA   Hochstrasser D.F., Frutiger S., Paquet N., Bairoch A., Ravier F.,
RA   Pasquali C., Sanchez J.-C., Tissot J.-D., Bjellqvist B., Vargas R.,
RA   Appel R.D., Hughes G.J.;
RT   "Human liver protein map: a reference database established by
RT   microsequencing and gel comparison.";
RL   Electrophoresis 13:992-1001(1992).
RN   [11]
RP   PROTEIN SEQUENCE OF 26-42.
RC   TISSUE=Mammary carcinoma;
RX   PubMed=9150946; DOI=10.1002/elps.1150180342;
RA   Rasmussen R.K., Ji H., Eddes J.S., Moritz R.L., Reid G.E.,
RA   Simpson R.J., Dorow D.S.;
RT   "Two-dimensional electrophoretic analysis of human breast carcinoma
RT   proteins: mapping of proteins that bind to the SH3 domain of mixed
RT   lineage kinase MLK2.";
RL   Electrophoresis 18:588-598(1997).
RN   [12]
RP   PROTEIN SEQUENCE OF 63-73; 95-104; 108-129; 131-140; 259-271; 297-304;
RP   306-329; 336-344; 352-362; 367-397; 434-460 AND 472-482, AND
RP   IDENTIFICATION BY MASS SPECTROMETRY.
RC   TISSUE=Brain, Cajal-Retzius cell, and Fetal brain cortex;
RA   Lubec G., Vishwanath V., Chen W.-Q., Sun Y.;
RL   Submitted (DEC-2008) to UniProtKB.
RN   [13]
RP   PROTEIN SEQUENCE OF 95-104 AND 472-479.
RC   TISSUE=Keratinocyte;
RX   PubMed=1286667; DOI=10.1002/elps.11501301199;
RA   Rasmussen H.H., van Damme J., Puype M., Gesser B., Celis J.E.,
RA   Vandekerckhove J.;
RT   "Microsequences of 145 proteins recorded in the two-dimensional gel
RT   protein database of normal human epidermal keratinocytes.";
RL   Electrophoresis 13:960-969(1992).
RN   [14]
RP   MASS SPECTROMETRY.
RC   TISSUE=Mammary cancer;
RX   PubMed=11840567;
RX   DOI=10.1002/1615-9861(200202)2:2<212::AID-PROT212>3.0.CO;2-H;
RA   Harris R.A., Yang A., Stein R.C., Lucy K., Brusten L., Herath A.,
RA   Parekh R., Waterfield M.D., O'Hare M.J., Neville M.A., Page M.J.,
RA   Zvelebil M.J.;
RT   "Cluster analysis of an extensive human breast cancer cell line
RT   protein expression map database.";
RL   Proteomics 2:212-223(2002).
RN   [15]
RP   SUBCELLULAR LOCATION [LARGE SCALE ANALYSIS].
RC   TISSUE=Melanoma;
RX   PubMed=12643545; DOI=10.1021/pr025562r;
RA   Basrur V., Yang F., Kushimoto T., Higashimoto Y., Yasumoto K.,
RA   Valencia J., Muller J., Vieira W.D., Watabe H., Shabanowitz J.,
RA   Hearing V.J., Hunt D.F., Appella E.;
RT   "Proteomic analysis of early melanosomes: identification of novel
RT   melanosomal proteins.";
RL   J. Proteome Res. 2:69-79(2003).
RN   [16]
RP   INTERACTION WITH ERP27.
RX   PubMed=16940051; DOI=10.1074/jbc.M604314200;
RA   Alanen H.I., Williamson R.A., Howard M.J., Hatahet F.S., Salo K.E.H.,
RA   Kauppila A., Kellokumpu S., Ruddock L.W.;
RT   "ERp27, a new non-catalytic endoplasmic reticulum-located human
RT   protein disulfide isomerase family member, interacts with ERp57.";
RL   J. Biol. Chem. 281:33727-33738(2006).
RN   [17]
RP   SUBCELLULAR LOCATION [LARGE SCALE ANALYSIS].
RC   TISSUE=Melanoma;
RX   PubMed=17081065; DOI=10.1021/pr060363j;
RA   Chi A., Valencia J.C., Hu Z.-Z., Watabe H., Yamaguchi H.,
RA   Mangini N.J., Huang H., Canfield V.A., Cheng K.C., Yang F., Abe R.,
RA   Yamagishi S., Shabanowitz J., Hearing V.J., Wu C., Appella E.,
RA   Hunt D.F.;
RT   "Proteomic and bioinformatic characterization of the biogenesis and
RT   function of melanosomes.";
RL   J. Proteome Res. 5:3135-3144(2006).
RN   [18]
RP   IDENTIFICATION BY MASS SPECTROMETRY [LARGE SCALE ANALYSIS].
RC   TISSUE=Leukemic T-cell;
RX   PubMed=19690332; DOI=10.1126/scisignal.2000007;
RA   Mayya V., Lundgren D.H., Hwang S.-I., Rezaul K., Wu L., Eng J.K.,
RA   Rodionov V., Han D.K.;
RT   "Quantitative phosphoproteomic analysis of T cell receptor signaling
RT   reveals system-wide modulation of protein-protein interactions.";
RL   Sci. Signal. 2:RA46-RA46(2009).
RN   [19]
RP   TISSUE SPECIFICITY.
RX   PubMed=20400973; DOI=10.1038/aja.2010.19;
RA   Kameshwari D.B., Bhande S., Sundaram C.S., Kota V., Siva A.B.,
RA   Shivaji S.;
RT   "Glucose-regulated protein precursor (GRP78) and tumor rejection
RT   antigen (GP96) are unique to hamster caput epididymal spermatozoa.";
RL   Asian J. Androl. 12:344-355(2010).
RN   [20]
RP   IDENTIFICATION BY MASS SPECTROMETRY [LARGE SCALE ANALYSIS].
RX   PubMed=21269460; DOI=10.1186/1752-0509-5-17;
RA   Burkard T.R., Planyavsky M., Kaupe I., Breitwieser F.P.,
RA   Buerckstuemmer T., Bennett K.L., Superti-Furga G., Colinge J.;
RT   "Initial characterization of the human central proteome.";
RL   BMC Syst. Biol. 5:17-17(2011).
RN   [21]
RP   IDENTIFICATION BY MASS SPECTROMETRY [LARGE SCALE ANALYSIS].
RX   PubMed=22905912; DOI=10.1021/pr300539b;
RA   Rosenow A., Noben J.P., Jocken J., Kallendrusch S.,
RA   Fischer-Posovszky P., Mariman E.C., Renes J.;
RT   "Resveratrol-induced changes of the human adipocyte secretion
RT   profile.";
RL   J. Proteome Res. 11:4733-4743(2012).
RN   [22]
RP   INTERACTION WITH SERPINA1 AND SERPINA2, AND SUBCELLULAR LOCATION.
RX   PubMed=23826168; DOI=10.1371/journal.pone.0066889;
RA   Marques P.I., Ferreira Z., Martins M., Figueiredo J., Silva D.I.,
RA   Castro P., Morales-Hojas R., Simoes-Correia J., Seixas S.;
RT   "SERPINA2 is a novel gene with a divergent function from SERPINA1.";
RL   PLoS ONE 8:E66889-E66889(2013).
RN   [23]
RP   PHOSPHORYLATION [LARGE SCALE ANALYSIS] AT THR-319, AND IDENTIFICATION
RP   BY MASS SPECTROMETRY [LARGE SCALE ANALYSIS].
RC   TISSUE=Liver;
RX   PubMed=24275569; DOI=10.1016/j.jprot.2013.11.014;
RA   Bian Y., Song C., Cheng K., Dong M., Wang F., Huang J., Sun D.,
RA   Wang L., Ye M., Zou H.;
RT   "An enzyme assisted RP-RPLC approach for in-depth analysis of human
RT   liver phosphoproteome.";
RL   J. Proteomics 96:253-262(2014).
RN   [24]
RP   CLEAVAGE OF SIGNAL PEPTIDE [LARGE SCALE ANALYSIS] AFTER ALA-24, AND
RP   IDENTIFICATION BY MASS SPECTROMETRY [LARGE SCALE ANALYSIS].
RX   PubMed=25944712; DOI=10.1002/pmic.201400617;
RA   Vaca Jacome A.S., Rabilloud T., Schaeffer-Reiss C., Rompais M.,
RA   Ayoub D., Lane L., Bairoch A., Van Dorsselaer A., Carapito C.;
RT   "N-terminome analysis of the human mitochondrial proteome.";
RL   Proteomics 15:2519-2524(2015).
RN   [25]
RP   STRUCTURE BY NMR OF 25-137.
RX   PubMed=16258833; DOI=10.1007/s10858-005-2720-1;
RA   Silvennoinen L., Koivunen P., Myllyharju J., Kilpelaeinen I.,
RA   Permi P.;
RT   "NMR assignment of the N-terminal domain a of the glycoprotein
RT   chaperone ERp57.";
RL   J. Biomol. NMR 33:136-136(2005).
RN   [26]
RP   STRUCTURE BY NMR OF 357-485.
RG   RIKEN structural genomics initiative (RSGI);
RT   "The solution structure of the second thioredoxin domain of human
RT   protein disulfide-isomerase A3.";
RL   Submitted (OCT-2006) to the PDB data bank.
RN   [27]
RP   X-RAY CRYSTALLOGRAPHY (2.0 ANGSTROMS) OF 134-376, AND INTERACTION WITH
RP   CANX.
RX   PubMed=16905107; DOI=10.1016/j.str.2006.06.019;
RA   Kozlov G., Maattanen P., Schrag J.D., Pollock S., Cygler M., Nagar B.,
RA   Thomas D.Y., Gehring K.;
RT   "Crystal structure of the bb' domains of the protein disulfide
RT   isomerase ERp57.";
RL   Structure 14:1331-1339(2006).
RN   [28]
RP   X-RAY CRYSTALLOGRAPHY (2.6 ANGSTROMS) OF 25-505 IN COMPLEX WITH TAPBP,
RP   SUBUNIT, INTERACTION WITH TAPBP, AND DISULFIDE BONDS.
RX   PubMed=19119025; DOI=10.1016/j.immuni.2008.10.018;
RA   Dong G., Wearsch P.A., Peaper D.R., Cresswell P., Reinisch K.M.;
RT   "Insights into MHC class I peptide loading from the structure of the
RT   tapasin-ERp57 thiol oxidoreductase heterodimer.";
RL   Immunity 30:21-32(2009).
CC   -!- CATALYTIC ACTIVITY: Catalyzes the rearrangement of -S-S- bonds in
CC       proteins. {ECO:0000269|PubMed:7487104}.
CC   -!- SUBUNIT: Subunit of the TAP complex, also known as the peptide
CC       loading complex (PLC). Can form disulfide-linked heterodimers with
CC       TAPBP. Interacts with ERP27 and CANX. Interacts with SERPINA2 and
CC       with the S and Z variants of SERPINA1.
CC       {ECO:0000269|PubMed:16905107, ECO:0000269|PubMed:16940051,
CC       ECO:0000269|PubMed:19119025, ECO:0000269|PubMed:23826168}.
CC   -!- INTERACTION:
CC       P05067:APP; NbExp=3; IntAct=EBI-979862, EBI-77613;
CC       P18418:Calr (xeno); NbExp=2; IntAct=EBI-979862, EBI-916742;
CC       P10909:CLU; NbExp=2; IntAct=EBI-979862, EBI-1104674;
CC       Q96HE7:ERO1L; NbExp=3; IntAct=EBI-979862, EBI-2564539;
CC       Q86YB8:ERO1LB; NbExp=2; IntAct=EBI-979862, EBI-2806988;
CC       Q13162:PRDX4; NbExp=2; IntAct=EBI-979862, EBI-2211957;
CC       Q13586:STIM1; NbExp=3; IntAct=EBI-979862, EBI-448878;
CC       Q03518:TAP1; NbExp=4; IntAct=EBI-979862, EBI-747259;
CC   -!- SUBCELLULAR LOCATION: Endoplasmic reticulum. Endoplasmic reticulum
CC       lumen {ECO:0000250}. Melanosome. Note=Identified by mass
CC       spectrometry in melanosome fractions from stage I to stage IV.
CC   -!- TISSUE SPECIFICITY: Detected in the flagellum and head region of
CC       spermatozoa (at protein level). {ECO:0000269|PubMed:20400973}.
CC   -!- MASS SPECTROMETRY: Mass=54265.22; Method=MALDI; Range=25-505;
CC       Evidence={ECO:0000269|PubMed:11840567};
CC   -!- SIMILARITY: Belongs to the protein disulfide isomerase family.
CC       {ECO:0000305}.
CC   -!- SIMILARITY: Contains 2 thioredoxin domains. {ECO:0000255|PROSITE-
CC       ProRule:PRU00691}.
CC   -!- CAUTION: Was originally thought to be a phosphatidylinositol 4,5-
CC       bisphosphate phosphodiesterase type I (phospholipase C-alpha).
CC       {ECO:0000305}.
DR   EMBL; D16234; BAA03759.1; -; mRNA.
DR   EMBL; U42068; AAC50331.1; -; mRNA.
DR   EMBL; Z49835; CAA89996.1; -; mRNA.
DR   EMBL; U75885; AAC51518.1; -; Genomic_DNA.
DR   EMBL; U75875; AAC51518.1; JOINED; Genomic_DNA.
DR   EMBL; U75876; AAC51518.1; JOINED; Genomic_DNA.
DR   EMBL; U75877; AAC51518.1; JOINED; Genomic_DNA.
DR   EMBL; U75878; AAC51518.1; JOINED; Genomic_DNA.
DR   EMBL; U75879; AAC51518.1; JOINED; Genomic_DNA.
DR   EMBL; U75880; AAC51518.1; JOINED; Genomic_DNA.
DR   EMBL; U75881; AAC51518.1; JOINED; Genomic_DNA.
DR   EMBL; U75882; AAC51518.1; JOINED; Genomic_DNA.
DR   EMBL; U75883; AAC51518.1; JOINED; Genomic_DNA.
DR   EMBL; U75884; AAC51518.1; JOINED; Genomic_DNA.
DR   EMBL; D83485; BAA11928.1; -; mRNA.
DR   EMBL; BC014433; AAH14433.1; -; mRNA.
DR   EMBL; BC036000; AAH36000.4; -; mRNA.
DR   EMBL; BC071878; AAH71878.1; -; mRNA.
DR   CCDS; CCDS10101.1; -.
DR   PIR; JC5704; JC5704.
DR   PIR; S55507; S55507.
DR   PIR; S63994; S63994.
DR   PIR; S68363; S68363.
DR   RefSeq; NP_005304.3; NM_005313.4.
DR   UniGene; Hs.591095; -.
DR   PDB; 2ALB; NMR; -; A=25-137.
DR   PDB; 2DMM; NMR; -; A=357-485.
DR   PDB; 2H8L; X-ray; 2.00 A; A/B/C=134-376.
DR   PDB; 3F8U; X-ray; 2.60 A; A/C=25-505.
DR   PDBsum; 2ALB; -.
DR   PDBsum; 2DMM; -.
DR   PDBsum; 2H8L; -.
DR   PDBsum; 3F8U; -.
DR   ProteinModelPortal; P30101; -.
DR   SMR; P30101; 25-493.
DR   BioGrid; 109180; 106.
DR   DIP; DIP-29132N; -.
DR   IntAct; P30101; 61.
DR   MINT; MINT-5000005; -.
DR   STRING; 9606.ENSP00000300289; -.
DR   PhosphoSite; P30101; -.
DR   BioMuta; PDIA3; -.
DR   DMDM; 2507461; -.
DR   DOSAC-COBS-2DPAGE; P30101; -.
DR   REPRODUCTION-2DPAGE; P30101; -.
DR   SWISS-2DPAGE; P30101; -.
DR   UCD-2DPAGE; P30101; -.
DR   PaxDb; P30101; -.
DR   PRIDE; P30101; -.
DR   DNASU; 2923; -.
DR   Ensembl; ENST00000300289; ENSP00000300289; ENSG00000167004.
DR   GeneID; 2923; -.
DR   KEGG; hsa:2923; -.
DR   UCSC; uc001zsu.3; human.
DR   CTD; 2923; -.
DR   GeneCards; PDIA3; -.
DR   HGNC; HGNC:4606; PDIA3.
DR   HPA; CAB011199; -.
DR   HPA; CAB015181; -.
DR   HPA; HPA002645; -.
DR   HPA; HPA003230; -.
DR   MIM; 602046; gene.
DR   neXtProt; NX_P30101; -.
DR   PharmGKB; PA29000; -.
DR   eggNOG; KOG0190; Eukaryota.
DR   eggNOG; COG0526; LUCA.
DR   HOGENOM; HOG000162459; -.
DR   HOVERGEN; HBG005920; -.
DR   InParanoid; P30101; -.
DR   KO; K08056; -.
DR   OMA; QINFAIA; -.
DR   OrthoDB; EOG7VHSX1; -.
DR   PhylomeDB; P30101; -.
DR   TreeFam; TF106382; -.
DR   BRENDA; 5.3.4.1; 2681.
DR   Reactome; R-HSA-1236974; ER-Phagosome pathway.
DR   Reactome; R-HSA-901042; Calnexin/calreticulin cycle.
DR   Reactome; R-HSA-983170; Antigen Presentation: Folding, assembly and peptide loading of class I MHC.
DR   ChiTaRS; PDIA3; human.
DR   EvolutionaryTrace; P30101; -.
DR   GenomeRNAi; 2923; -.
DR   NextBio; 11593; -.
DR   PRO; PR:P30101; -.
DR   Proteomes; UP000005640; Chromosome 15.
DR   Bgee; P30101; -.
DR   CleanEx; HS_PDIA3; -.
DR   ExpressionAtlas; P30101; baseline and differential.
DR   Genevisible; P30101; HS.
DR   GO; GO:0009986; C:cell surface; IDA:MGI.
DR   GO; GO:0005783; C:endoplasmic reticulum; IDA:UniProtKB.
DR   GO; GO:0005788; C:endoplasmic reticulum lumen; TAS:Reactome.
DR   GO; GO:0070062; C:extracellular exosome; IDA:UniProtKB.
DR   GO; GO:0005925; C:focal adhesion; IDA:UniProtKB.
DR   GO; GO:0042470; C:melanosome; IEA:UniProtKB-SubCell.
DR   GO; GO:0043209; C:myelin sheath; IEA:Ensembl.
DR   GO; GO:0005634; C:nucleus; IDA:UniProtKB.
DR   GO; GO:0005790; C:smooth endoplasmic reticulum; IEA:Ensembl.
DR   GO; GO:0004197; F:cysteine-type endopeptidase activity; TAS:ProtInc.
DR   GO; GO:0015036; F:disulfide oxidoreductase activity; TAS:ParkinsonsUK-UCL.
DR   GO; GO:0004629; F:phospholipase C activity; TAS:ProtInc.
DR   GO; GO:0044822; F:poly(A) RNA binding; IDA:UniProtKB.
DR   GO; GO:0003756; F:protein disulfide isomerase activity; TAS:ProtInc.
DR   GO; GO:0042590; P:antigen processing and presentation of exogenous peptide antigen via MHC class I; TAS:Reactome.
DR   GO; GO:0002479; P:antigen processing and presentation of exogenous peptide antigen via MHC class I, TAP-dependent; TAS:Reactome.
DR   GO; GO:0002474; P:antigen processing and presentation of peptide antigen via MHC class I; TAS:Reactome.
DR   GO; GO:0045454; P:cell redox homeostasis; IEA:InterPro.
DR   GO; GO:0044267; P:cellular protein metabolic process; TAS:Reactome.
DR   GO; GO:0055114; P:oxidation-reduction process; TAS:GOC.
DR   GO; GO:2001238; P:positive regulation of extrinsic apoptotic signaling pathway; IEA:Ensembl.
DR   GO; GO:0043687; P:post-translational protein modification; TAS:Reactome.
DR   GO; GO:0006457; P:protein folding; TAS:Reactome.
DR   GO; GO:0034975; P:protein folding in endoplasmic reticulum; TAS:ParkinsonsUK-UCL.
DR   GO; GO:0006606; P:protein import into nucleus; TAS:ProtInc.
DR   GO; GO:0018279; P:protein N-linked glycosylation via asparagine; TAS:Reactome.
DR   GO; GO:0006621; P:protein retention in ER lumen; TAS:ProtInc.
DR   GO; GO:0006508; P:proteolysis; TAS:GOC.
DR   GO; GO:0034976; P:response to endoplasmic reticulum stress; IBA:GO_Central.
DR   GO; GO:0007165; P:signal transduction; TAS:ProtInc.
DR   Gene3D; 3.40.30.10; -; 3.
DR   InterPro; IPR005788; Disulphide_isomerase.
DR   InterPro; IPR005792; Prot_disulphide_isomerase.
DR   InterPro; IPR012336; Thioredoxin-like_fold.
DR   InterPro; IPR017937; Thioredoxin_CS.
DR   InterPro; IPR013766; Thioredoxin_domain.
DR   Pfam; PF00085; Thioredoxin; 2.
DR   SUPFAM; SSF52833; SSF52833; 4.
DR   TIGRFAMs; TIGR01130; ER_PDI_fam; 1.
DR   TIGRFAMs; TIGR01126; pdi_dom; 2.
DR   PROSITE; PS00194; THIOREDOXIN_1; 2.
DR   PROSITE; PS51352; THIOREDOXIN_2; 2.
PE   1: Evidence at protein level;
KW   3D-structure; Acetylation; Complete proteome;
KW   Direct protein sequencing; Disulfide bond; Endoplasmic reticulum;
KW   Isomerase; Phosphoprotein; Polymorphism; Redox-active center;
KW   Reference proteome; Repeat; Signal.
FT   SIGNAL        1     24       {ECO:0000244|PubMed:25944712,
FT                                ECO:0000269|PubMed:12665801,
FT                                ECO:0000269|PubMed:1286669,
FT                                ECO:0000269|PubMed:7487104,
FT                                ECO:0000269|PubMed:9150948,
FT                                ECO:0000269|PubMed:9399589}.
FT   CHAIN        25    505       Protein disulfide-isomerase A3.
FT                                /FTId=PRO_0000034225.
FT   DOMAIN       25    133       Thioredoxin 1. {ECO:0000255|PROSITE-
FT                                ProRule:PRU00691}.
FT   DOMAIN      343    485       Thioredoxin 2. {ECO:0000255|PROSITE-
FT                                ProRule:PRU00691}.
FT   MOTIF       502    505       Prevents secretion from ER.
FT                                {ECO:0000250}.
FT   ACT_SITE     57     57       Nucleophile. {ECO:0000250}.
FT   ACT_SITE     60     60       Nucleophile. {ECO:0000250}.
FT   ACT_SITE    406    406       Nucleophile. {ECO:0000250}.
FT   ACT_SITE    409    409       Nucleophile. {ECO:0000250}.
FT   SITE         58     58       Contributes to redox potential value.
FT                                {ECO:0000250}.
FT   SITE         59     59       Contributes to redox potential value.
FT                                {ECO:0000250}.
FT   SITE        119    119       Lowers pKa of C-terminal Cys of first
FT                                active site. {ECO:0000250}.
FT   SITE        407    407       Contributes to redox potential value.
FT                                {ECO:0000250}.
FT   SITE        408    408       Contributes to redox potential value.
FT                                {ECO:0000250}.
FT   SITE        471    471       Lowers pKa of C-terminal Cys of second
FT                                active site. {ECO:0000250}.
FT   MOD_RES     129    129       N6-succinyllysine.
FT                                {ECO:0000250|UniProtKB:P27773}.
FT   MOD_RES     152    152       N6-acetyllysine.
FT                                {ECO:0000250|UniProtKB:P27773}.
FT   MOD_RES     218    218       N6-succinyllysine.
FT                                {ECO:0000250|UniProtKB:P27773}.
FT   MOD_RES     252    252       N6-acetyllysine.
FT                                {ECO:0000250|UniProtKB:P27773}.
FT   MOD_RES     319    319       Phosphothreonine.
FT                                {ECO:0000244|PubMed:24275569}.
FT   MOD_RES     362    362       N6-acetyllysine.
FT                                {ECO:0000250|UniProtKB:P27773}.
FT   MOD_RES     494    494       N6-acetyllysine.
FT                                {ECO:0000250|UniProtKB:P27773}.
FT   DISULFID     57     60       Redox-active. {ECO:0000255|PROSITE-
FT                                ProRule:PRU00691}.
FT   DISULFID     57     57       Interchain (with C-115 in TAPBP); in
FT                                linked form.
FT                                {ECO:0000269|PubMed:19119025}.
FT   DISULFID     85     92       {ECO:0000269|PubMed:19119025}.
FT   DISULFID    406    409       Redox-active. {ECO:0000255|PROSITE-
FT                                ProRule:PRU00691}.
FT   VARIANT     415    415       K -> R (in dbSNP:rs6413485).
FT                                /FTId=VAR_020027.
FT   MUTAGEN      57     57       C->A: No loss of activity. No loss of
FT                                activity; when associated with A-406.
FT                                {ECO:0000269|PubMed:9399589}.
FT   MUTAGEN      57     57       C->S: Activity changed to serine
FT                                protease. {ECO:0000269|PubMed:9399589}.
FT   MUTAGEN      60     60       C->S: Activity changed to serine
FT                                protease; when associated with S-409.
FT                                {ECO:0000269|PubMed:9399589}.
FT   MUTAGEN     406    406       C->A: No loss of activity. No loss of
FT                                activity; when associated with A-57.
FT                                {ECO:0000269|PubMed:9399589}.
FT   MUTAGEN     406    406       C->S: Activity changed to serine
FT                                protease. {ECO:0000269|PubMed:9399589}.
FT   MUTAGEN     409    409       C->S: Activity changed to serine
FT                                protease; when associated with S-60.
FT                                {ECO:0000269|PubMed:9399589}.
FT   CONFLICT     19     19       A -> G (in Ref. 6; BAA11928).
FT                                {ECO:0000305}.
FT   CONFLICT     22     22       A -> V (in Ref. 6; BAA11928).
FT                                {ECO:0000305}.
FT   CONFLICT    217    217       D -> Y (in Ref. 1; BAA03759).
FT                                {ECO:0000305}.
FT   CONFLICT    225    225       Q -> P (in Ref. 4; CAA89996).
FT                                {ECO:0000305}.
FT   CONFLICT    238    238       E -> G (in Ref. 4; CAA89996).
FT                                {ECO:0000305}.
FT   CONFLICT    272    272       N -> D (in Ref. 1; BAA03759 and 5;
FT                                AAC51518). {ECO:0000305}.
FT   CONFLICT    355    355       D -> G (in Ref. 1; BAA03759).
FT                                {ECO:0000305}.
FT   CONFLICT    358    358       D -> G (in Ref. 1; BAA03759).
FT                                {ECO:0000305}.
FT   CONFLICT    368    368       E -> D (in Ref. 1; BAA03759).
FT                                {ECO:0000305}.
FT   STRAND       27     29       {ECO:0000244|PDB:2ALB}.
FT   TURN         32     34       {ECO:0000244|PDB:3F8U}.
FT   HELIX        35     38       {ECO:0000244|PDB:3F8U}.
FT   HELIX        39     41       {ECO:0000244|PDB:2ALB}.
FT   STRAND       43     53       {ECO:0000244|PDB:3F8U}.
FT   HELIX        58     73       {ECO:0000244|PDB:3F8U}.
FT   TURN         74     77       {ECO:0000244|PDB:3F8U}.
FT   STRAND       80     84       {ECO:0000244|PDB:3F8U}.
FT   TURN         85     87       {ECO:0000244|PDB:3F8U}.
FT   HELIX        89     94       {ECO:0000244|PDB:3F8U}.
FT   STRAND       99    107       {ECO:0000244|PDB:3F8U}.
FT   STRAND      110    114       {ECO:0000244|PDB:3F8U}.
FT   HELIX       121    131       {ECO:0000244|PDB:3F8U}.
FT   STRAND      136    138       {ECO:0000244|PDB:2H8L}.
FT   HELIX       142    149       {ECO:0000244|PDB:2H8L}.
FT   STRAND      151    153       {ECO:0000244|PDB:2H8L}.
FT   STRAND      155    161       {ECO:0000244|PDB:2H8L}.
FT   HELIX       166    177       {ECO:0000244|PDB:2H8L}.
FT   TURN        178    181       {ECO:0000244|PDB:2H8L}.
FT   STRAND      182    187       {ECO:0000244|PDB:2H8L}.
FT   HELIX       190    196       {ECO:0000244|PDB:2H8L}.
FT   STRAND      198    206       {ECO:0000244|PDB:2H8L}.
FT   HELIX       209    211       {ECO:0000244|PDB:2H8L}.
FT   STRAND      218    221       {ECO:0000244|PDB:2H8L}.
FT   HELIX       229    239       {ECO:0000244|PDB:2H8L}.
FT   TURN        240    243       {ECO:0000244|PDB:3F8U}.
FT   TURN        249    251       {ECO:0000244|PDB:2H8L}.
FT   HELIX       252    255       {ECO:0000244|PDB:2H8L}.
FT   STRAND      256    265       {ECO:0000244|PDB:2H8L}.
FT   TURN        269    271       {ECO:0000244|PDB:2H8L}.
FT   HELIX       273    292       {ECO:0000244|PDB:2H8L}.
FT   STRAND      298    303       {ECO:0000244|PDB:2H8L}.
FT   TURN        304    307       {ECO:0000244|PDB:2H8L}.
FT   HELIX       308    311       {ECO:0000244|PDB:2H8L}.
FT   HELIX       312    314       {ECO:0000244|PDB:2H8L}.
FT   STRAND      325    329       {ECO:0000244|PDB:2H8L}.
FT   STRAND      331    333       {ECO:0000244|PDB:3F8U}.
FT   STRAND      335    337       {ECO:0000244|PDB:2H8L}.
FT   HELIX       347    358       {ECO:0000244|PDB:2H8L}.
FT   STRAND      376    381       {ECO:0000244|PDB:3F8U}.
FT   TURN        383    385       {ECO:0000244|PDB:3F8U}.
FT   HELIX       386    390       {ECO:0000244|PDB:3F8U}.
FT   STRAND      396    402       {ECO:0000244|PDB:3F8U}.
FT   HELIX       407    422       {ECO:0000244|PDB:3F8U}.
FT   TURN        423    425       {ECO:0000244|PDB:3F8U}.
FT   STRAND      427    435       {ECO:0000244|PDB:3F8U}.
FT   TURN        436    438       {ECO:0000244|PDB:2DMM}.
FT   STRAND      449    456       {ECO:0000244|PDB:3F8U}.
FT   HELIX       473    483       {ECO:0000244|PDB:3F8U}.
SQ   SEQUENCE   505 AA;  56782 MW;  529E5B6692D0D7E9 CRC64;
     MRLRRLALFP GVALLLAAAR LAAASDVLEL TDDNFESRIS DTGSAGLMLV EFFAPWCGHC
     KRLAPEYEAA ATRLKGIVPL AKVDCTANTN TCNKYGVSGY PTLKIFRDGE EAGAYDGPRT
     ADGIVSHLKK QAGPASVPLR TEEEFKKFIS DKDASIVGFF DDSFSEAHSE FLKAASNLRD
     NYRFAHTNVE SLVNEYDDNG EGIILFRPSH LTNKFEDKTV AYTEQKMTSG KIKKFIQENI
     FGICPHMTED NKDLIQGKDL LIAYYDVDYE KNAKGSNYWR NRVMMVAKKF LDAGHKLNFA
     VASRKTFSHE LSDFGLESTA GEIPVVAIRT AKGEKFVMQE EFSRDGKALE RFLQDYFDGN
     LKRYLKSEPI PESNDGPVKV VVAENFDEIV NNENKDVLIE FYAPWCGHCK NLEPKYKELG
     EKLSKDPNIV IAKMDATAND VPSPYEVRGF PTIYFSPANK KLNPKKYEGG RELSDFISYL
     QREATNPPVI QEEKPKKKKK AQEDL
```

|  |
| --- |
| **Mascot:** http://www.matrixscience.com/ |
