# Supplementary material for: Proteomic profiling identifies the inorganic pyrophosphatase (PPA1) protein as a potential biomarker of metastasis in laryngeal squamous cell carcinoma
Source: Amino Acids. 2016 Mar 7;48:1469–76. doi: 10.1007/s00726-016-2201-8 (PMC4875942; doi:10.1007/s00726-016-2201-8)
Supplement: Supplementary file 9 — Supplementary material 9 (HTML 10 kb) [file 726_2016_2201_MOESM9_ESM.html]

Mascot Search Results: IPYR\_HUMAN


# MASCOT Search Results

## Protein View: IPYR\_HUMAN

### Inorganic pyrophosphatase OS=Homo sapiens GN=PPA1 PE=1 SV=2

|  |  |
| --- | --- |
| Database: | SwissProt |
| Score: | 161 |
| Expect: | 1.6e-12 |
| Nominal mass (Mr): | 33095 |
| Calculated pI: | 5.54 |
| Taxonomy: | Homo sapiens |

Sequence similarity is available as an NCBI BLAST search of IPYR\_HUMAN against nr.

### Search parameters

|  |  |
| --- | --- |
| MS data file: | `peaklist.xml` |
| Enzyme: | Trypsin: cuts C-term side of KR unless next residue is P. |
| Fixed modifications: | Carbamidomethyl (C) |
| Variable modifications: | Oxidation (M) |
|  |  |
| --- | --- |
| Mass values searched: | 20 |
| Mass values matched: | 12 |

### Protein sequence coverage: 52%

Matched peptides shown in ***bold red***.

|  |  |  |  |  |  |
| --- | --- | --- | --- | --- | --- |
| `1` | `MSGFSTEERA` | `APFSLEYRVF` | `LKNEKGQYIS` | `PFHDIPIYAD` | `KDVFHMVVEV` |
| `51` | `PRWSNAKMEI` | `ATKDPLNPIK` | `QDVKKGKLRY` | `VANLFPYKGY` | `IWNYGAIPQT` |
| `101` | `WEDPGHNDKH` | `TGCCGDNDPI` | `DVCEIGSKVC` | `ARGEIIGVKV` | `LGILAMIDEG` |
| `151` | `ETDWKVIAIN` | `VDDPDAANYN` | `DINDVKRLKP` | `GYLEATVDWF` | `RRYKVPDGKP` |
| `201` | `ENEFAFNAEF` | `KDKDFAIDII` | `KSTHDHWKAL` | `VTKKTNGKGI` | `SCMNTTLSES` |
| `251` | `PFKCDPDAAR` | `AIVDALPPPC` | `ESACTVPTDV` | `DKWFHHQKN` |  |

Unformatted sequence string: 289 residues (for pasting into other applications).

Residue Number

Increasing Mass

Decreasing Mass

| Start | – | End | Observed | Mr(expt) | Mr(calc) | Delta | M | Peptide |
| --- | --- | --- | --- | --- | --- | --- | --- | --- |
| 26 | – | 41 | 1864.0424 | 1863.0351 | 1862.9203 | 0.1148 | 0 | K.GQYISPFHDIPIYADK.D |
| 42 | – | 52 | 1327.7828 | 1326.7756 | 1326.6755 | 0.1001 | 0 | K.DVFHMVVEVPR.W |
| 42 | – | 52 | 1343.7549 | 1342.7476 | 1342.6704 | 0.0772 | 0 | K.DVFHMVVEVPR.W + Oxidation (M) |
| 80 | – | 88 | 1114.6392 | 1113.6319 | 1113.5859 | 0.0460 | 0 | R.YVANLFPYK.G |
| 89 | – | 109 | 2461.2522 | 2460.2449 | 2460.1135 | 0.1315 | 0 | K.GYIWNYGAIPQTWEDPGHNDK.H |
| 110 | – | 128 | 2133.9922 | 2132.9849 | 2132.8562 | 0.1287 | 0 | K.HTGCCGDNDPIDVCEIGSK.V |
| 156 | – | 176 | 2288.2305 | 2287.2232 | 2287.0968 | 0.1264 | 0 | K.VIAINVDDPDAANYNDINDVK.R |
| 178 | – | 191 | 1695.0149 | 1694.0076 | 1693.8828 | 0.1248 | 0 | R.LKPGYLEATVDWFR.R |
| 195 | – | 211 | 1939.0413 | 1938.0340 | 1937.9159 | 0.1181 | 0 | K.VPDGKPENEFAFNAEFK.D |
| 212 | – | 221 | 1177.6986 | 1176.6913 | 1176.6390 | 0.0523 | 1 | K.DKDFAIDIIK.S |
| 222 | – | 228 | 910.4728 | 909.4656 | 909.4093 | 0.0562 | 0 | K.STHDHWK.A |
| 283 | – | 288 | 882.4840 | 881.4767 | 881.4297 | 0.0471 | 0 | K.WFHHQK.N |

`No match to: 823.5417, 855.1078, 870.6130, 954.4739, 966.5745, 1349.7058, 2239.2605, 2356.2471`

---

```
AC   Q15181; Q2M348; Q5SQT7; Q6P7P4; Q9UQJ5; Q9Y5B1;
DT   01-NOV-1997, integrated into UniProtKB/Swiss-Prot.
DT   30-MAY-2000, sequence version 2.
DT   09-DEC-2015, entry version 158.
DE   RecName: Full=Inorganic pyrophosphatase;
DE            EC=3.6.1.1;
DE   AltName: Full=Pyrophosphate phospho-hydrolase;
DE            Short=PPase;
GN   Name=PPA1; Synonyms=IOPPP, PP;
OS   Homo sapiens (Human).
OC   Eukaryota; Metazoa; Chordata; Craniata; Vertebrata; Euteleostomi;
OC   Mammalia; Eutheria; Euarchontoglires; Primates; Haplorrhini;
OC   Catarrhini; Hominidae; Homo.
OX   NCBI_TaxID=9606;
RN   [1]
RP   NUCLEOTIDE SEQUENCE [MRNA], AND TISSUE SPECIFICITY.
RC   TISSUE=Heart;
RX   PubMed=10542310; DOI=10.1016/S0167-4781(99)00175-X;
RA   Fairchild T.A., Patejunas G.;
RT   "Cloning and expression profile of human inorganic pyrophosphatase.";
RL   Biochim. Biophys. Acta 1447:133-136(1999).
RN   [2]
RP   NUCLEOTIDE SEQUENCE [MRNA].
RC   TISSUE=Brain;
RA   Saito T., Hattori A., Miyajima N.;
RT   "Putative inorganic pyrophosphatase.";
RL   Submitted (APR-1999) to the EMBL/GenBank/DDBJ databases.
RN   [3]
RP   NUCLEOTIDE SEQUENCE [MRNA].
RA   Kanni L., Johansson M., Karlsson A.;
RT   "Cloning of a human inorganic pyrophosphatase cDNA.";
RL   Submitted (DEC-1999) to the EMBL/GenBank/DDBJ databases.
RN   [4]
RP   NUCLEOTIDE SEQUENCE [MRNA].
RA   Dai F.Y., Yu L., Hu P.R., Xin Y.R., Xu Y.F., Zhao S.Y.;
RT   "Cloning and characterization of a novel human cDNA homology to bovine
RT   inorganic pyrophosphatase mRNA.";
RL   Submitted (JUL-2003) to the EMBL/GenBank/DDBJ databases.
RN   [5]
RP   NUCLEOTIDE SEQUENCE [LARGE SCALE MRNA].
RC   TISSUE=Adrenal gland;
RX   PubMed=10931946; DOI=10.1073/pnas.160270997;
RA   Hu R.-M., Han Z.-G., Song H.-D., Peng Y.-D., Huang Q.-H., Ren S.-X.,
RA   Gu Y.-J., Huang C.-H., Li Y.-B., Jiang C.-L., Fu G., Zhang Q.-H.,
RA   Gu B.-W., Dai M., Mao Y.-F., Gao G.-F., Rong R., Ye M., Zhou J.,
RA   Xu S.-H., Gu J., Shi J.-X., Jin W.-R., Zhang C.-K., Wu T.-M.,
RA   Huang G.-Y., Chen Z., Chen M.-D., Chen J.-L.;
RT   "Gene expression profiling in the human hypothalamus-pituitary-adrenal
RT   axis and full-length cDNA cloning.";
RL   Proc. Natl. Acad. Sci. U.S.A. 97:9543-9548(2000).
RN   [6]
RP   NUCLEOTIDE SEQUENCE [LARGE SCALE GENOMIC DNA].
RX   PubMed=15164054; DOI=10.1038/nature02462;
RA   Deloukas P., Earthrowl M.E., Grafham D.V., Rubenfield M., French L.,
RA   Steward C.A., Sims S.K., Jones M.C., Searle S., Scott C., Howe K.,
RA   Hunt S.E., Andrews T.D., Gilbert J.G.R., Swarbreck D., Ashurst J.L.,
RA   Taylor A., Battles J., Bird C.P., Ainscough R., Almeida J.P.,
RA   Ashwell R.I.S., Ambrose K.D., Babbage A.K., Bagguley C.L., Bailey J.,
RA   Banerjee R., Bates K., Beasley H., Bray-Allen S., Brown A.J.,
RA   Brown J.Y., Burford D.C., Burrill W., Burton J., Cahill P., Camire D.,
RA   Carter N.P., Chapman J.C., Clark S.Y., Clarke G., Clee C.M., Clegg S.,
RA   Corby N., Coulson A., Dhami P., Dutta I., Dunn M., Faulkner L.,
RA   Frankish A., Frankland J.A., Garner P., Garnett J., Gribble S.,
RA   Griffiths C., Grocock R., Gustafson E., Hammond S., Harley J.L.,
RA   Hart E., Heath P.D., Ho T.P., Hopkins B., Horne J., Howden P.J.,
RA   Huckle E., Hynds C., Johnson C., Johnson D., Kana A., Kay M.,
RA   Kimberley A.M., Kershaw J.K., Kokkinaki M., Laird G.K., Lawlor S.,
RA   Lee H.M., Leongamornlert D.A., Laird G., Lloyd C., Lloyd D.M.,
RA   Loveland J., Lovell J., McLaren S., McLay K.E., McMurray A.,
RA   Mashreghi-Mohammadi M., Matthews L., Milne S., Nickerson T.,
RA   Nguyen M., Overton-Larty E., Palmer S.A., Pearce A.V., Peck A.I.,
RA   Pelan S., Phillimore B., Porter K., Rice C.M., Rogosin A., Ross M.T.,
RA   Sarafidou T., Sehra H.K., Shownkeen R., Skuce C.D., Smith M.,
RA   Standring L., Sycamore N., Tester J., Thorpe A., Torcasso W.,
RA   Tracey A., Tromans A., Tsolas J., Wall M., Walsh J., Wang H.,
RA   Weinstock K., West A.P., Willey D.L., Whitehead S.L., Wilming L.,
RA   Wray P.W., Young L., Chen Y., Lovering R.C., Moschonas N.K.,
RA   Siebert R., Fechtel K., Bentley D., Durbin R.M., Hubbard T.,
RA   Doucette-Stamm L., Beck S., Smith D.R., Rogers J.;
RT   "The DNA sequence and comparative analysis of human chromosome 10.";
RL   Nature 429:375-381(2004).
RN   [7]
RP   NUCLEOTIDE SEQUENCE [LARGE SCALE MRNA].
RC   TISSUE=Brain, Lymph, and Uterus;
RX   PubMed=15489334; DOI=10.1101/gr.2596504;
RG   The MGC Project Team;
RT   "The status, quality, and expansion of the NIH full-length cDNA
RT   project: the Mammalian Gene Collection (MGC).";
RL   Genome Res. 14:2121-2127(2004).
RN   [8]
RP   NUCLEOTIDE SEQUENCE [MRNA] OF 5-286.
RA   Rumsfeld J., Ziegelbauer K., Spaltmann F.;
RT   "Cloning, expression, affinity purification and characterization of
RT   polyhistidine-tagged cytosolic Saccharomyces cerevisiae and human
RT   inorganic pyrophosphatases for differential screening of compounds for
RT   antifungal activity.";
RL   Submitted (NOV-1998) to the EMBL/GenBank/DDBJ databases.
RN   [9]
RP   PROTEIN SEQUENCE OF 10-18; 26-41; 58-70; 80-88; 110-128; 140-191;
RP   193-221 AND 239-253, AND IDENTIFICATION BY MASS SPECTROMETRY.
RC   TISSUE=Brain, Cajal-Retzius cell, and Fetal brain cortex;
RA   Lubec G., Afjehi-Sadat L., Chen W.-Q., Sun Y.;
RL   Submitted (DEC-2008) to UniProtKB.
RN   [10]
RP   NUCLEOTIDE SEQUENCE [MRNA] OF 83-196.
RA   Lacroix J., Vigneron M., Kedinger C.;
RT   "Partial sequence of the human inorganic pyrophosphatase.";
RL   Submitted (MAR-1995) to the EMBL/GenBank/DDBJ databases.
RN   [11]
RP   ACETYLATION [LARGE SCALE ANALYSIS] AT SER-2, CLEAVAGE OF INITIATOR
RP   METHIONINE [LARGE SCALE ANALYSIS], AND IDENTIFICATION BY MASS
RP   SPECTROMETRY [LARGE SCALE ANALYSIS].
RX   PubMed=19413330; DOI=10.1021/ac9004309;
RA   Gauci S., Helbig A.O., Slijper M., Krijgsveld J., Heck A.J.,
RA   Mohammed S.;
RT   "Lys-N and trypsin cover complementary parts of the phosphoproteome in
RT   a refined SCX-based approach.";
RL   Anal. Chem. 81:4493-4501(2009).
RN   [12]
RP   ACETYLATION [LARGE SCALE ANALYSIS] AT LYS-57 AND LYS-228, AND
RP   IDENTIFICATION BY MASS SPECTROMETRY [LARGE SCALE ANALYSIS].
RX   PubMed=19608861; DOI=10.1126/science.1175371;
RA   Choudhary C., Kumar C., Gnad F., Nielsen M.L., Rehman M.,
RA   Walther T.C., Olsen J.V., Mann M.;
RT   "Lysine acetylation targets protein complexes and co-regulates major
RT   cellular functions.";
RL   Science 325:834-840(2009).
RN   [13]
RP   PHOSPHORYLATION [LARGE SCALE ANALYSIS] AT SER-250, AND IDENTIFICATION
RP   BY MASS SPECTROMETRY [LARGE SCALE ANALYSIS].
RC   TISSUE=Cervix carcinoma;
RX   PubMed=20068231; DOI=10.1126/scisignal.2000475;
RA   Olsen J.V., Vermeulen M., Santamaria A., Kumar C., Miller M.L.,
RA   Jensen L.J., Gnad F., Cox J., Jensen T.S., Nigg E.A., Brunak S.,
RA   Mann M.;
RT   "Quantitative phosphoproteomics reveals widespread full
RT   phosphorylation site occupancy during mitosis.";
RL   Sci. Signal. 3:RA3-RA3(2010).
RN   [14]
RP   IDENTIFICATION BY MASS SPECTROMETRY [LARGE SCALE ANALYSIS].
RX   PubMed=21269460; DOI=10.1186/1752-0509-5-17;
RA   Burkard T.R., Planyavsky M., Kaupe I., Breitwieser F.P.,
RA   Buerckstuemmer T., Bennett K.L., Superti-Furga G., Colinge J.;
RT   "Initial characterization of the human central proteome.";
RL   BMC Syst. Biol. 5:17-17(2011).
RN   [15]
RP   ACETYLATION [LARGE SCALE ANALYSIS] AT SER-2, CLEAVAGE OF INITIATOR
RP   METHIONINE [LARGE SCALE ANALYSIS], AND IDENTIFICATION BY MASS
RP   SPECTROMETRY [LARGE SCALE ANALYSIS].
RX   PubMed=22223895; DOI=10.1074/mcp.M111.015131;
RA   Bienvenut W.V., Sumpton D., Martinez A., Lilla S., Espagne C.,
RA   Meinnel T., Giglione C.;
RT   "Comparative large-scale characterisation of plant vs. mammal proteins
RT   reveals similar and idiosyncratic N-alpha acetylation features.";
RL   Mol. Cell. Proteomics 11:M111.015131-M111.015131(2012).
RN   [16]
RP   ACETYLATION [LARGE SCALE ANALYSIS] AT SER-2, CLEAVAGE OF INITIATOR
RP   METHIONINE [LARGE SCALE ANALYSIS], AND IDENTIFICATION BY MASS
RP   SPECTROMETRY [LARGE SCALE ANALYSIS].
RX   PubMed=22814378; DOI=10.1073/pnas.1210303109;
RA   Van Damme P., Lasa M., Polevoda B., Gazquez C., Elosegui-Artola A.,
RA   Kim D.S., De Juan-Pardo E., Demeyer K., Hole K., Larrea E.,
RA   Timmerman E., Prieto J., Arnesen T., Sherman F., Gevaert K.,
RA   Aldabe R.;
RT   "N-terminal acetylome analyses and functional insights of the N-
RT   terminal acetyltransferase NatB.";
RL   Proc. Natl. Acad. Sci. U.S.A. 109:12449-12454(2012).
RN   [17]
RP   IDENTIFICATION BY MASS SPECTROMETRY [LARGE SCALE ANALYSIS].
RC   TISSUE=Liver;
RX   PubMed=24275569; DOI=10.1016/j.jprot.2013.11.014;
RA   Bian Y., Song C., Cheng K., Dong M., Wang F., Huang J., Sun D.,
RA   Wang L., Ye M., Zou H.;
RT   "An enzyme assisted RP-RPLC approach for in-depth analysis of human
RT   liver phosphoproteome.";
RL   J. Proteomics 96:253-262(2014).
RN   [18]
RP   VARIANT [LARGE SCALE ANALYSIS] ASN-57.
RX   PubMed=16959974; DOI=10.1126/science.1133427;
RA   Sjoeblom T., Jones S., Wood L.D., Parsons D.W., Lin J., Barber T.D.,
RA   Mandelker D., Leary R.J., Ptak J., Silliman N., Szabo S.,
RA   Buckhaults P., Farrell C., Meeh P., Markowitz S.D., Willis J.,
RA   Dawson D., Willson J.K.V., Gazdar A.F., Hartigan J., Wu L., Liu C.,
RA   Parmigiani G., Park B.H., Bachman K.E., Papadopoulos N.,
RA   Vogelstein B., Kinzler K.W., Velculescu V.E.;
RT   "The consensus coding sequences of human breast and colorectal
RT   cancers.";
RL   Science 314:268-274(2006).
CC   -!- CATALYTIC ACTIVITY: Diphosphate + H(2)O = 2 phosphate.
CC   -!- COFACTOR:
CC       Name=Mg(2+); Xref=ChEBI:CHEBI:18420; Evidence={ECO:0000250};
CC       Note=Binds 4 Mg(2+) ions per subunit. Other metal ions can support
CC       activity, but at a lower rate. Two Mg(2+) ions are required for
CC       the activation of the enzyme and are present before substrate
CC       binds, two additional Mg(2+) ions form complexes with substrate
CC       and product. {ECO:0000250};
CC   -!- SUBUNIT: Homodimer. {ECO:0000250}.
CC   -!- SUBCELLULAR LOCATION: Cytoplasm {ECO:0000250}.
CC   -!- TISSUE SPECIFICITY: Expressed ubiquitously.
CC       {ECO:0000269|PubMed:10542310}.
CC   -!- SIMILARITY: Belongs to the PPase family. {ECO:0000305}.
DR   EMBL; AF154065; AAD34643.1; -; mRNA.
DR   EMBL; AB026723; BAA84702.1; -; mRNA.
DR   EMBL; AF217186; AAG36780.1; -; mRNA.
DR   EMBL; AF092439; AAP97214.1; -; mRNA.
DR   EMBL; AF119665; AAF17222.1; -; mRNA.
DR   EMBL; AL731540; CAI13692.1; -; Genomic_DNA.
DR   EMBL; BC001022; AAH01022.3; -; mRNA.
DR   EMBL; BC061581; AAH61581.2; -; mRNA.
DR   EMBL; BC105034; AAI05035.1; -; mRNA.
DR   EMBL; BC105036; AAI05037.1; -; mRNA.
DR   EMBL; BC107882; AAI07883.1; -; mRNA.
DR   EMBL; AF108211; AAD24964.1; -; mRNA.
DR   EMBL; Z48605; CAA88494.1; -; mRNA.
DR   CCDS; CCDS7299.1; -.
DR   RefSeq; NP_066952.1; NM_021129.3.
DR   UniGene; Hs.437403; -.
DR   ProteinModelPortal; Q15181; -.
DR   SMR; Q15181; 4-284.
DR   BioGrid; 111460; 33.
DR   IntAct; Q15181; 9.
DR   MINT; MINT-3030968; -.
DR   STRING; 9606.ENSP00000362329; -.
DR   PhosphoSite; Q15181; -.
DR   BioMuta; PPA1; -.
DR   DMDM; 8247940; -.
DR   REPRODUCTION-2DPAGE; IPI00015018; -.
DR   PaxDb; Q15181; -.
DR   PeptideAtlas; Q15181; -.
DR   PRIDE; Q15181; -.
DR   DNASU; 5464; -.
DR   Ensembl; ENST00000373232; ENSP00000362329; ENSG00000180817.
DR   GeneID; 5464; -.
DR   KEGG; hsa:5464; -.
DR   UCSC; uc001jqv.1; human.
DR   CTD; 5464; -.
DR   GeneCards; PPA1; -.
DR   H-InvDB; HIX0032502; -.
DR   HGNC; HGNC:9226; PPA1.
DR   HPA; HPA019878; -.
DR   HPA; HPA020096; -.
DR   MIM; 179030; gene.
DR   neXtProt; NX_Q15181; -.
DR   PharmGKB; PA33550; -.
DR   eggNOG; KOG1626; Eukaryota.
DR   eggNOG; COG0221; LUCA.
DR   HOGENOM; HOG000195569; -.
DR   HOVERGEN; HBG000491; -.
DR   InParanoid; Q15181; -.
DR   KO; K01507; -.
DR   OMA; GHKDENT; -.
DR   OrthoDB; EOG7R2BKH; -.
DR   PhylomeDB; Q15181; -.
DR   TreeFam; TF300887; -.
DR   BRENDA; 3.6.1.1; 2681.
DR   Reactome; R-HSA-379716; Cytosolic tRNA aminoacylation.
DR   ChiTaRS; PPA1; human.
DR   GenomeRNAi; 5464; -.
DR   NextBio; 21148; -.
DR   PRO; PR:Q15181; -.
DR   Proteomes; UP000005640; Chromosome 10.
DR   Bgee; Q15181; -.
DR   CleanEx; HS_PPA1; -.
DR   ExpressionAtlas; Q15181; baseline and differential.
DR   Genevisible; Q15181; HS.
DR   GO; GO:0005737; C:cytoplasm; TAS:UniProtKB.
DR   GO; GO:0005829; C:cytosol; TAS:Reactome.
DR   GO; GO:0070062; C:extracellular exosome; IDA:UniProtKB.
DR   GO; GO:0004427; F:inorganic diphosphatase activity; EXP:Reactome.
DR   GO; GO:0000287; F:magnesium ion binding; IEA:InterPro.
DR   GO; GO:0071344; P:diphosphate metabolic process; TAS:Reactome.
DR   GO; GO:0010467; P:gene expression; TAS:Reactome.
DR   GO; GO:0006796; P:phosphate-containing compound metabolic process; TAS:ProtInc.
DR   GO; GO:0006418; P:tRNA aminoacylation for protein translation; TAS:Reactome.
DR   Gene3D; 3.90.80.10; -; 1.
DR   InterPro; IPR008162; Pyrophosphatase.
DR   PANTHER; PTHR10286; PTHR10286; 1.
DR   Pfam; PF00719; Pyrophosphatase; 1.
DR   SUPFAM; SSF50324; SSF50324; 1.
DR   PROSITE; PS00387; PPASE; 1.
PE   1: Evidence at protein level;
KW   Acetylation; Complete proteome; Cytoplasm; Direct protein sequencing;
KW   Hydrolase; Magnesium; Metal-binding; Phosphoprotein; Polymorphism;
KW   Reference proteome.
FT   INIT_MET      1      1       Removed. {ECO:0000244|PubMed:19413330,
FT                                ECO:0000244|PubMed:22223895,
FT                                ECO:0000244|PubMed:22814378}.
FT   CHAIN         2    289       Inorganic pyrophosphatase.
FT                                /FTId=PRO_0000137567.
FT   METAL       116    116       Magnesium 1. {ECO:0000250}.
FT   METAL       121    121       Magnesium 1. {ECO:0000250}.
FT   METAL       121    121       Magnesium 2. {ECO:0000250}.
FT   METAL       153    153       Magnesium 1. {ECO:0000250}.
FT   MOD_RES       2      2       N-acetylserine.
FT                                {ECO:0000244|PubMed:19413330,
FT                                ECO:0000244|PubMed:22223895,
FT                                ECO:0000244|PubMed:22814378}.
FT   MOD_RES      57     57       N6-acetyllysine.
FT                                {ECO:0000244|PubMed:19608861}.
FT   MOD_RES     228    228       N6-acetyllysine.
FT                                {ECO:0000244|PubMed:19608861}.
FT   MOD_RES     250    250       Phosphoserine.
FT                                {ECO:0000244|PubMed:20068231}.
FT   VARIANT      57     57       K -> N (in a breast cancer sample;
FT                                somatic mutation).
FT                                {ECO:0000269|PubMed:16959974}.
FT                                /FTId=VAR_036358.
FT   CONFLICT     12     12       P -> A (in Ref. 8; AAD24964).
FT                                {ECO:0000305}.
FT   CONFLICT     84     84       L -> I (in Ref. 10; CAA88494).
FT                                {ECO:0000305}.
FT   CONFLICT     96     97       AI -> TL (in Ref. 10; CAA88494).
FT                                {ECO:0000305}.
FT   CONFLICT    105    114       GHNDKHTGCC -> HEKDKSTNCF (in Ref. 10;
FT                                CAA88494). {ECO:0000305}.
FT   CONFLICT    129    140       VCARGEIIGVKV -> ILSCGEVIHVKI (in Ref. 10;
FT                                CAA88494). {ECO:0000305}.
FT   CONFLICT    146    146       M -> L (in Ref. 10; CAA88494).
FT                                {ECO:0000305}.
FT   CONFLICT    156    156       V -> L (in Ref. 10; CAA88494).
FT                                {ECO:0000305}.
FT   CONFLICT    161    162       VD -> AN (in Ref. 10; CAA88494).
FT                                {ECO:0000305}.
FT   CONFLICT    165    173       DAANYNDIN -> EASKFHDID (in Ref. 10;
FT                                CAA88494). {ECO:0000305}.
FT   CONFLICT    177    178       RL -> KF (in Ref. 10; CAA88494).
FT                                {ECO:0000305}.
FT   CONFLICT    187    188       VD -> LN (in Ref. 10; CAA88494).
FT                                {ECO:0000305}.
FT   CONFLICT    192    192       R -> L (in Ref. 10; CAA88494).
FT                                {ECO:0000305}.
SQ   SEQUENCE   289 AA;  32660 MW;  E3973C9E6F8CA5CD CRC64;
     MSGFSTEERA APFSLEYRVF LKNEKGQYIS PFHDIPIYAD KDVFHMVVEV PRWSNAKMEI
     ATKDPLNPIK QDVKKGKLRY VANLFPYKGY IWNYGAIPQT WEDPGHNDKH TGCCGDNDPI
     DVCEIGSKVC ARGEIIGVKV LGILAMIDEG ETDWKVIAIN VDDPDAANYN DINDVKRLKP
     GYLEATVDWF RRYKVPDGKP ENEFAFNAEF KDKDFAIDII KSTHDHWKAL VTKKTNGKGI
     SCMNTTLSES PFKCDPDAAR AIVDALPPPC ESACTVPTDV DKWFHHQKN
```

|  |
| --- |
| **Mascot:** http://www.matrixscience.com/ |
